# Supplementary material for: Extinction of eastern Sahul megafauna coincides with sustained environmental deterioration
Source: Nat Commun. 2020 May 18;11:2250. doi: 10.1038/s41467-020-15785-w (PMC7231803; doi:10.1038/s41467-020-15785-w)
Supplement: Supplementary file 1 — Supplementary Information [file 41467_2020_15785_MOESM1_ESM.pdf]

Supplementary Information

Extinction of eastern Sahul megafauna coincides with sustained environmental deterioration

Hocknull et al.

Contents

Extinction of eastern Sahul megafauna coincides with sustained environmental deterioration........... 1

List of Figures and Tables 3

Supplementary Note 1: Systematic identification of megafauna remains 6

CROCODYLIA Owen, 1842 8

Crocodylus sp. cf. *C. porosus* Schneider, 1801 10

Pallimnarchus de Vis, 1886 10

'*Quinkana*' sp. 11

Dromaius sp. cf. *D. novaehollandiae* 13

Varanus priscus (Owen, 1859) 13

Varanus sp. (large) 14

Macropodidae Gray, 1821 16

Notamacropus Dawson & Flannery 1985 16

Osphranter sp. cf. *O. rufus* (Desmarest, 1822) 17

Macropus Shaw, 1790 17

Protemnodon Owen, 1874 19

Sthenurinae Glauert, 1926 20

Palorchestidae Owen, 1874 23

Palorchestes Owen, 1874 23

Thylacoleonidae Gill, 1872 24

Thylacoleo sp. 24

Vombatidae Burnett, 1830 24

Sedophascolomys sp. cf. *S. medius* (Owen, 1872) 24

Phascolonus gigas (Owen, 1858) 25
Diprotodontidae Owen, 1838 27
Diprotodon optatum Owen, 1838 27
Zygomaturus trilobus (Macleay, 1857) 28
Supplementary Note 2: Faunal Associations 30
Supplementary Note 3: Body mass estimation for *Macropus* sp. (giant) 32
Supplementary Note 4: Stratigraphic and Sedimentological description of QML1470 sites. 33
QML1470 (SW9) (Supplementary Figure 11 & Supplementary Table 4) 34
QML1470 (SW3) (Supplementary Figure 12 & Supplementary Table 5) 41
QML1470 (SWJ) (Supplementary Figure 13 & Supplementary Table 6) 44
SWCC (Supplementary Figure 14 & Supplementary Table 7) 45
Supplementary Note 5: Geochronology of South Walker Creek (QML1470) sites 47
Methodology – Field Collection 48
Site QML1470 (SW9) 48
Site QML1470 (SW3) 50
Site QML1470 (SWJ) 50
QML1470 (SWCC) 50
Optically Stimulated Luminescence 51
Richard Lewis, Lee J. Arnold, Tim Pietsch and Jon Olley. 51
Sample acquisition and preparation 51
Instrumentation and Equivalent dose (D_e) measurement 52
Dose rate evaluation and age calculation 55
Dose Recovery Test 61
SWC-CC 61
SWJ 62
SW3 63
SW9 – AU (Unit C – main fossil unit) 64
SW9 – AU (sand lens samples) 64
SW9 – GU (Unit C – main fossil unit) 65
Supplementary Note 6: Radiocarbon Dating 76
Supplementary Note 7: U-series direct dating of South Walker Creek megafauna fossils. 79

Supplementary Note 8: U-series laser ablation and ESR direct dating of South Walker Creek megafauna fossils.......83

Sample description and methods.......83

In-situ U-series analysis.......83

Modelling of open-system U-series age.......84

U-series for ESR model.......85

ESR protocol.......85

RESULTS.......86

Open-system U-series ages.......86

US-ESR age modelling.......90

Supplementary Note 9: Palynological Assessment of QML1470 (SW9).......93

Supplementary References.......95

List of Figures and Tables

Supplementary Figure 1. Plants, bivalves, crustaceans, insects and fish remains from QML1470 (SW9) and QML1470 (SW3).......7

Supplementary Table 1. Crocodilian tooth and osteoderm characteristics used to identify isolated remains at South Walker Creek sites.......9

Supplementary Figure 2. Crocodylidae and Chelidae.......12

Supplementary Figure 3. Casuariidae and Varanidae.......15

Supplementary Figure 4. Box plots of tibia lengths (mm) for macropodids from published records of museum specimens.......21

(Over Page) Supplementary Figure 5. Macropodidae.......23

Supplementary Figure 6. Palorchestidae, Thylacoleonidae and Vombatidae.......26

Supplementary Table 2. Dental measurements of *Diprotodon optatum* from South Walker Creek.......27

Supplementary Figure 7. Diprotodontidae.......29

Supplementary Table 3. Faunal.......30

Supplementary Table 4. QML1470 (SW9) Sedimentological and Taphonomic Summary and Interpretation.......37

Supplementary Figure 8. Sediment classification and composition following Folk.......37

Supplementary Figure 9. Percentage of bone elements represented by Voorhies Group per site.......38

Supplementary Figure 10. Taphonomic examples.......39

Supplementary Figure 11. Stratigraphic map of QML1470 (SW9).......40

Supplementary Table 5. QML1470 (SW3) Sedimentological and Taphonomic Summary and Interpretation.......42

Supplementary Figure 12. Stratigraphic map of QML1470 (SW3).......43

Supplementary Table 6. QML1470 (SWJ) Sedimentological and Taphonomic Summary and Interpretation.......44

Supplementary Figure 13. Stratigraphic map of QML1470 (SWJ).......45

Supplementary Table 7. QML1470 (SWCC) Sedimentological and Taphonomic Summary and Interpretation.......46

Supplementary Figure 14. Stratigraphic map of QML1470 (SWCC).......47

Supplementary Figure 15. QML1420 (SW9), dating sample locations within the SW9.......49

Supplementary Figure 16. Example of a contaminated single-grain D_e distribution from the preliminary group of OSL samples collected in 2009 (sample SW9-Basal). 58
Supplementary Figure 17. Examples of a single-grain OSL decay and dose response. 59
Supplementary Figure 18. Multi-grain dose recovery test results obtained for sample SWC-B after administering a dose of 100 Gy (uncertainties are shown at 1σ). 60
Supplementary Figure 19. Radial plots showing single-grain OSL dose recovery test results obtained for samples SWC17-53, SWC-B and SWC-G using the AU SAR protocol shown in Table S1 (D_e errors are shown at 1σ). 61
Supplementary Figure 20. Radial plots showing the single-grain OSL D_e distributions obtained for the South Walker Creek, SWC-CC samples (D_e errors are shown at 1σ). 61
Supplementary Figure 21. Radial plots showing the single-grain OSL D_e distributions obtained for the South Walker Creek, SWJ samples (D_e errors are shown at 1σ). 62
Supplementary Figure 22. Radial plots showing the single-grain OSL D_e distributions obtained for the South Walker Creek, SW3 samples (D_e errors are shown at 1σ). 63
Supplementary Figure 23. Radial plots showing the single-grain OSL D_e distributions obtained for the South Walker Creek, SW9 samples (D_e errors are shown at 1σ) that were taken from the main fossil unit (Unit C; including both sediments in association with fossil remains and those surrounding fossil remains). 64
Supplementary Figure 24. Radial plots showing the single-grain OSL D_e distributions obtained for the South Walker Creek, SW9 samples (D_e errors are shown at 1σ) that were extracted from sand intrusions. 64
Supplementary Figure 24. Radial plots showing the single-grain OSL D_e distributions obtained for the South Walker Creek, SW9 (GU) samples (D_e errors are shown at 1σ) constraining the vertical and horizontal age distribution of the main fossil unit (Unit C). 65
Supplementary Table 8. SAR protocols used by the Adelaide University (AU) and Griffith University (GU) laboratories in this study to obtain single-grain OSL ages. 66
Supplementary Table 9. Single-grain dose recovery results obtained for 212-250 µm quartz grains of samples SWC17-53, SWC-B and SWC-G using the AU SAR protocol shown in Supplementary Table 8. n = number of accepted De values; OD = overdispersion value. 66
Supplementary Table 10. Single-grain OSL statistics showing proportion of rejected and accepted grains after applying the SAR rejection criteria to the 2015 and 2017 samples. 67
Supplementary Table 11. Dose rate data, single-grain equivalent doses (D_e) and quartz OSL ages for the 2009, 2015 and 2017 SWC samples. 68
Supplementary Table 12. Log likelihood (llik) statistics[98, 99] associated with the of De populations of the South Walker Creek samples. 69
Supplementary Table 13. Finite mixture model fitting results for sample SW9-11, which exhibits a complex De distribution with multiple dose components. 70
Supplementary Table 14. Finite mixture model fitting results for sample SWC17-49, which exhibits a complex De distribution with multiple dose components. 71
Supplementary Table 15. Environmental dose rate values calculated for the quartz fractions of the 2011, 2015 and 2017 OSL samples. 72
[f] Cosmic-ray dose rates were calculated using the approach of Prescott and Hutton [83] and assigned a relative uncertainty of ±10%. 73
Supplementary Table 16. High-resolution gamma spectrometry (HRGS) results for the OSL samples from South Walker Creek. 73
Supplementary Table 17. Weighted mean ages for OSL samples collected from the fossil-bearing and associated non-fossiliferous sediment of the each site. 75
Supplementary Table 18. Details of the OSL samples used to calculate the weighted mean ages for the fossil units at each site, as shown in Supplementary Table 17. 75

Supplementary Table 19. Radiocarbon Dates. Calibration is against SHCal13 (Hogg et al. 2013) in OxCal (Ramsey 2009). * Date may extend beyond calibration curve. 78
Supplementary Table 20. U-series results for micro-profiled teeth and measured in the Radiogenic Isotope Facility at The University of Queensland. 81
Supplementary Figure 27. SW-1A-E *Pallimnarchus* sp. tooth QMF59869, QML1470 (SW9) 82
Supplementary Figure 28. SW9-31- *Diprotodon optatum* QMF54689 M_4 (associated mandible), QML1470 (SW9) 82
Supplementary Figure 29. SW9-66 *Sedophascolomys* sp. cf. *S. medius* (associated incisors) QMF57069, QML1470 (SW9) 82
Supplementary Figure 30. SW13 – *Phascolonus gigas* molar (associated remains) QMF57065, QML1470 (SW9) 82
Supplementary Table 21. Uranium-series isotope data for the teeth and the coral standard (MK16) produced at University of Wollongong 87
Supplementary Figure 31. Measured (a) (234U/238U) and (b) (230Th/238U) activity ratios (blue dots) and modelled ratios (red dots) for QML1470 incisor I-SCU-02. 89
Supplementary Figure 32. Measured (a) (234U/238U) and (b) (230Th/238U) activity ratios (blue dots) and modelled ratios (red dots) for QML1470 SW9-2015 P-SCU01 dentine. 90
Supplementary Figure 33. Dose Response curve (DRC) were calculated using MCDoseE 2.0, with 250k iterations and Burning of 125k and a Double Saturated Exponential (DSE) function (Joannes-Boyau et al., 2018). .. 91
Supplementary Table 22. US-ESR dating data for SW9 teeth. a Dose equivalent De obtained using McDoseE 2.0, with DSE [128]. 92
Supplementary Figure 34. Identified pollen counts from QML1470 (SW9). 93
Supplementary Table 23. Pollen, habitat interpretation, *Sporomiella* (dung fungus) and Charcoal counts for ten subsamples (SW1-SW10) from QML1470 (SW9). 94

Supplementary Note 1: Systematic identification of megafauna remains

Scott A. Hocknull & Rochelle A. Lawrence

Taxonomic assignment of megafauna fossils recovered from the SWC sites and nearby Kemmis Creek and Homevale Station are justified here. This work was undertaken to develop a base taxonomic list of megafauna species recovered from each site discussed in the main text and listed in Table 2. The taxonomic identifications were also used to assist in determining associations of cranial and post-cranial material within fossil deposits.

Identifications were made using published characteristics for known species, direct morphological and morphometric comparisons with securely identified megafauna from the Queensland Museum Geosciences collection, and via correspondence with institutions holding material valuable for identification. Taxonomic identifications of megafauna were complicated due to very poor characterisation of most Pleistocene species. Where possible, specimens were compared directly with taxonomic type material.

Other non-megafauna fossil remains such as small aquatic vertebrates and invertebrates, terrestrial insects and flora have also been found at these sites and help justify the depositional settings. Examples of these are figured in Supplementary Figure 1, a-z & 2, u. Detailed taxonomic appraisals of these remains will form part of a full systematic treatment elsewhere.

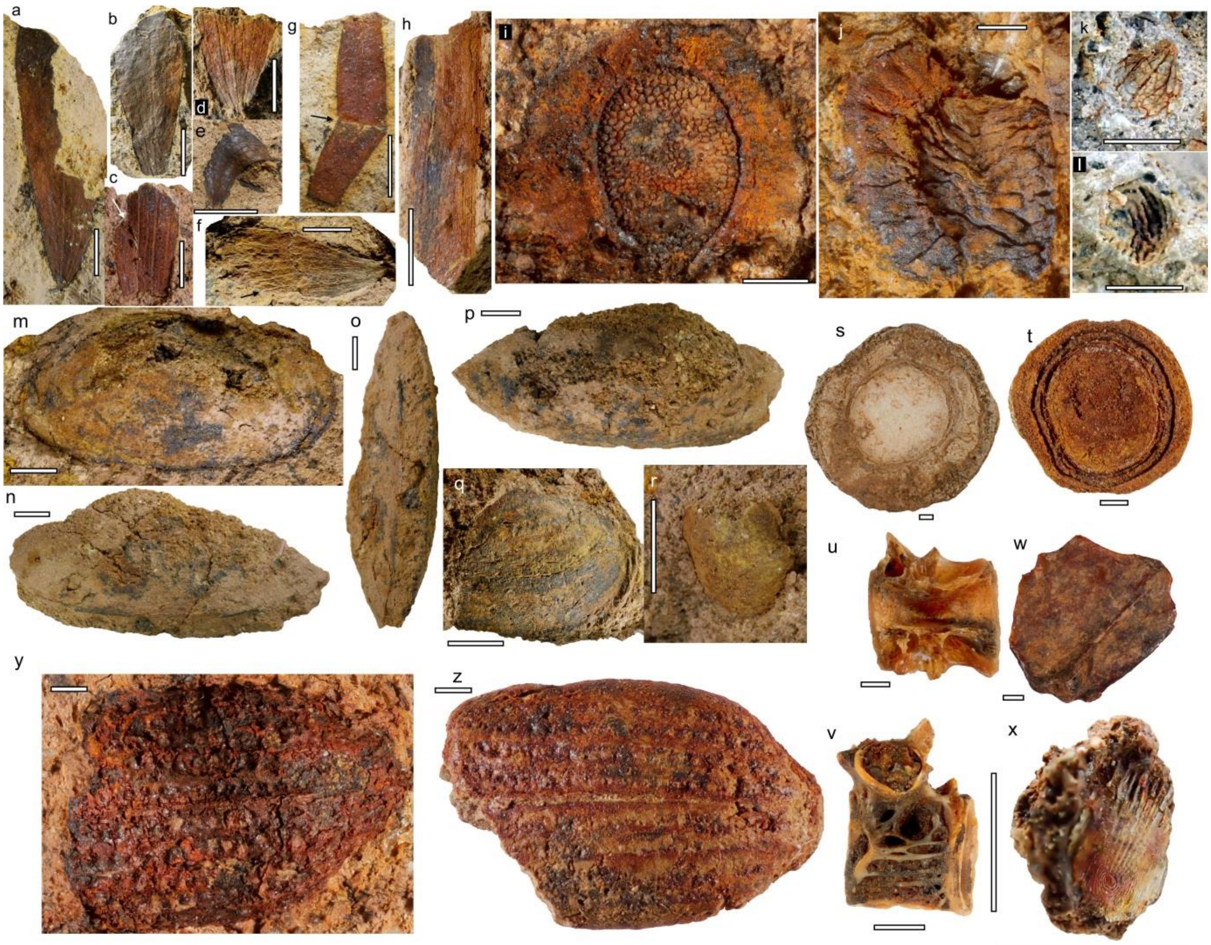

Supplementary Figure 1. Plants, bivalves, crustaceans, insects and fish remains from QML1470 (SW9) and QML1470 (SW3). Acacia sp. (a–e) partial leaves showing thick parallel venation, dried leaf split (arrow) in (c) and dried leaf curl in (e); (a) QMF59891, (b) QMF59892, (c) QMF59893, (d) QMF60003, (e) QMF59900. Melaleuca sp. (f) partial leaf showing fine reticulate venation (arrow) QMF59899. ?Eucalyptus sp. (g) partial leaf showing mid-leaf dry breakage pattern (arrow) QMF59906. ?Lomandra sp. (h) strap leaf showing fine parallel venation and soft leaf deformation QMF59903. Goodeniaceae (i) seed showing soft anatomy QMF59895. ?Allocasuarina sp. (j) seed showing internal anatomy QMF59896. Unidentified seeds (k) QMF59898 and (l) QMF59905. Bivalves, Velesunio wilsoni (m–q) articulated and isolated valves; (m) QMF59904, (n–p) QMF59890, (q) QMF59902. Corbiculidae (r) indeterminate species isolated valve QMF59889. Crustacean gastroliths (‘yabby buttons’) (s–t); (s) QMF59894 and (t) QMF59901. Teleostei (u-x); (u–v) vertebra QMF59908; (w) operculum QMF59907; (x) scale. Insecta, ?Curculionidae (y–z) articulated weevil elytron QMF59897. Scale bars equal 5 mm (a–h & m–t) and 1 mm (i–l & u–z).

CROCODYLIA Owen, 1842

Crocodylidae Cuvier, 1807

Isolated teeth (both shed and broken), osteoderms, fragmentary skull elements, a partial edentulous posterior dentary and isolated coprolites represent crocodilian remains recovered from QML1470 (SW9, SW3, SWCC & SWJ). Three or possibly four distinctive crocodilian tooth morphologies are present in addition to two different osteoderm morphologies. Comparisons have been made with large collections of isolated teeth and osteoderms from Queensland Paleogene and Neogene fossil sites; these include associated teeth and osteoderms from described cranial and postcranial remains in addition to a large comparative collection of teeth and osteoderms from extant species of crocodilians[1, 2, 3, 4, 5, 6, 7]. A preliminary identification of tooth morphotype and osteoderms to known taxa is provided here (Supplementary Table 1). A more thorough assessment of crocodilian tooth and osteoderm morphological diversity from Australian Neogene fossil sites is underway[8] to further test and refine the identification traits established previously for teeth[7, 9] whilst combining this with morphological diversity in osteoderms.

Supplementary Table 1. Crocodilian tooth and osteoderm characteristics used to identify isolated remains at South Walker Creek sites**.**

		**Tooth Morphology**				**Osteoderm Morphology**	
**Morphotype**	**Taxon**	**Profile & base shape**	**Serrations**	**Denticles**	**Fluting**	**Osteoderm base**	**Osteoderm keel and surface pitting**
1	*Crocodylus porosus*	Gracile conical, circular	Faint	Absent	Strong	Shape – Circular to ovoid with thick bone base. Bone texture – irregular. ragged edge.	Tall, curved surface with irregular pitting.
2	*Pallimnarchus gracilis*	Robust conical, circular	Fine	Absent	Faint	Shape – oblong to rectangular with thick bone base. Bone texture – smooth, regular edge.	Low, straight surface with regular large pits.
3	*Pallimnarchus pollens*	Robust conical, circular	Thick	Absent	Absent	Shape – oblong to rectangular with thick bone base. Bone texture – smooth, regular edge.	Low, straight surface with regular large pits.
4	'*Quinkana*' sp. ziphodont crocodilian	Tapered asymmetrical, labio-lingually compressed	Thick	Present	Absent		

Crocodylus Laurenti, 1768

Crocodylus sp. cf. *C. porosus* Schneider, 1801

Supplementary Figure 2 (a-h)

Referred material: QML1470. Osteoderm QMF57048; teeth QMF57085, QMF59863, QMF59867, QMF57058, QMF57076, QMF57077, QMF59864.

The presence of crocodilian morphotype 1 suggests the presence of *C. porosus* at several sites (QML1470 SW9, SW3, SWCC). Isolated teeth and a single osteoderm very closely match the morphology of extant and extinct species of *Crocodylus porosus*. In particular, the shape of the osteoderm compares very favourably with that observed in *C. porosus* (Supplementary Table 1) and to that in other observed species of *Crocodylus* (i.e. *C. niloticus, C. novaeguineae, C. johnstoni*). The presence of a species of *Crocodylus* likely that of *C. porosus* is not surprising because *C. porosus* is presently located in the lower reaches of the Fitzroy River, into which Walker Creek drains. However, the site occurs near the head waters of the Fitzroy River catchment, which suggests a major range contraction downstream over the Upper Pleistocene and evidently continued into the Holocene. In addition, *C. porosus* has been identified from Pliocene[2] sites found in the Burdekin River catchment, which is adjacent to the Fitzroy River catchment to the North; therefore, this taxon has a well-established longevity in the region.

Pallimnarchus de Vis, 1886

Pallimnarchus gracilis Willis & Molnar, 1997

Pallimnarchus pollens de Vis, 1886

Supplementary Figure 2 (i-p, t)

Referred material: QML1470. Osteoderm QMF57094; teeth QMF59868, QMF57095, QMF59861, QMF57051, QMF59866, QMF59865; right dentary (partial) QMF57092.

Crocodilian Morphotype 2 + 3. As with the first morphotype, osteoderms representing a species of *Pallimnarchus* species are rare. However, they are distinctively broad, rectangular-ovoid in shape, flat with a low keel, a thick smooth base with large regular surface pitting, which differs substantially from those osteoderms of *C. porosus* (Supplementary Table 1). Osteoderms found in association with the partial skull and postcranial remains of QMF1752 from Lansdowne, near Tambo, ~440kms south west of South Walker Creek, provide the only known associated cranial specimen with osteoderms and dentition. Willis and Molnar [6] identify QMF1752 as *Pallimnarchus gracilis*, therefore, the associated osteoderms and teeth can be used to help differentiate this species from the type species, *P. pollens*. Based on comparisons with large numbers of isolated teeth from the Queensland Museum collection there are two different morphotypes for teeth pertaining to *Pallimnarchus*: *P. pollens* are conical with no fluting and thick serrations, whilst the teeth associated with *P. gracilis* specimens are conical with faint fluting and fine serrations. Both morphotypes occur at South Walker Creek sites (QML1470 SW9 and SW3) suggesting the presence of both species but we

will treat these initial differences with caution pending thorough analysis. In the current study we tentatively consider these two separate morphotypes as a singular taxonomic grouping.

A near complete skull, without teeth, of *Pallimnarchus gracilis* was recovered from a nearby creek at Homevale Station[6], therefore, the presence of *P. gracilis* from South Walker Creek is not surprising. The presence of the more robust, typically southern distributed species[6], *P. pollens*, is new and may represent the northern limit of the species' geographic. This is plausible considering the catchment that abuts the southern perimeter of the Fitzroy River catchment is the upper reaches of the Condamine River (Murray-Darling Basin), which includes the type locality for *P. pollens* and the majority of its recorded occurrences. Directly adjacent to the western margin of Fitzroy River catchment is the west-draining Lake Eyre basin, which also records the presence of *P. pollens*[6]. The southern-most record of *P. gracilis* occurs along the catchment abutment between these two basins at Landsdowne, indicating that these species could cross over major drainage divides.

'*Quinkana*' sp.

Supplementary Figure 2 (q-s)

Referred material: QML1470. Teeth QMF59862, QMF59870 & QMF57062.

Crocodilian Morphotype 4 (Supplementary Table 1). Isolated, labio-lingually compressed denticulate teeth represent a fourth morphotype of tooth found at South Walker Creek. These teeth are easily distinguished by the presence of clearly denticulate carinae. The only other tooth morphotype commonly encountered in Australian Pleistocene sites with denticulate carinae are those of varanids, in particular the giant *Varanus priscus*. Differentiating this tooth morphotype from *V. priscus* and all other varanids is straight forward with varanid teeth being considerably more labio-lingually compressed, and possessing both greater crown curvature and plicidentine. Crocodilians with this type of ziphodont dentition are generally considered to be closest to *Quinkana*; however, direct comparison of these teeth referred to *Quinkana* (e.g. QMF10141, QMF23220, QMF51503 and QMF58607) reveals the teeth to be much larger, less labio-lingual compressed and straighter, thus not a direct match to the SWC teeth (or other denticulate teeth observed from other localities within Queensland[8]). These teeth are less compressed and we term them 'semi-ziphodont' to differentiate them from the highly compressed or conical crocodilian dentition typically found in Australian Neogene sites[4, 7, 8, 9, 10]. The tooth morphology indicates the presence of a large crocodilian unlike that of *Crocodylus* or *Pallimnarchus* within the fauna but bearing some resemblance to specimens referred to as *Quinkana*. Direct comparison of the South Walker Creek teeth to ziphodont dentition recovered from Middle Pleistocene cave deposits at Mt. Etna[11, 12, 13] indicates an additional morphotype of crocodilian living in the Fitzroy River Basin during the Quaternary, bringing the total to at least four.

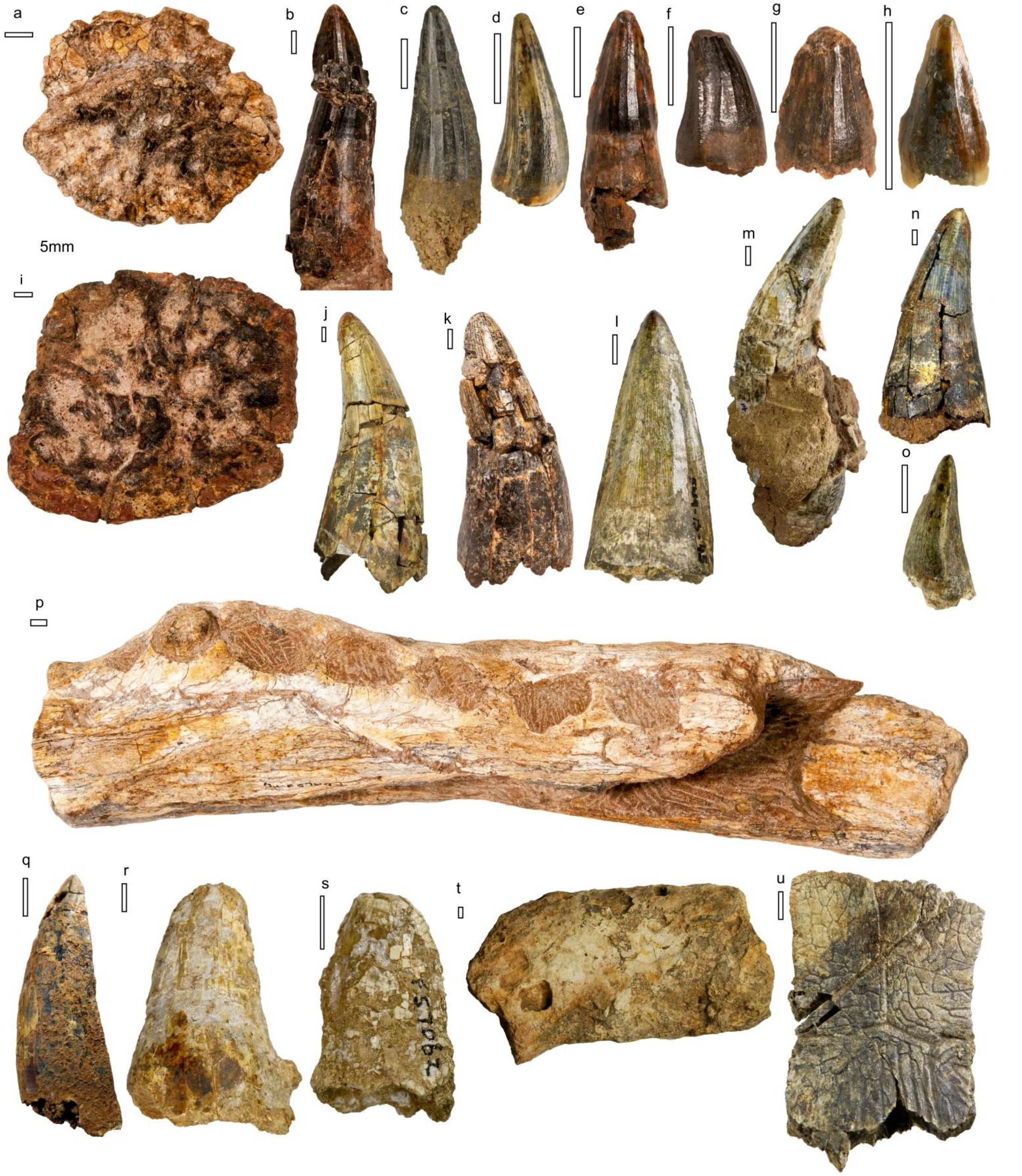

Supplementary Figure 2. Crocodylidae and Chelidae. *Crocodylus* sp. (a) osteoderm QMF57048; (b–h) teeth; (b) QMF57085, (c) QMF59863, (d) QMF59867, (e) QMF57058, (f) QMF57076, (g) QMF57077, (h) QMF59864. *Pallimnarchus* sp. (i) osteoderm QMF57094; (j–o) teeth; (j) QMF59868, (k) QMF57095, (l) QMF59861, (m) QMF57051, (n) QMF59866, (o) QMF59865; (p) right dentary (partial) QMF57092. '*Quinkana*'sp. (q–s); teeth (q) QMF59862, (r) QMF59870, (s) QMF57062; (t) coprolite QMF59960. Chelidae indet. (u) carapace QMF59909. Scale bars = 5 mm.

AVES

Casuariidae Kaup 1847

Dromaius Vieillot, 1816

Dromaius sp. cf. *D. novaehollandiae*

Supplementary Figure 3 (a & b)

Referred material: QML1470. Distal tarsometatarsus QMF57167; egg shell QMF60000.

A giant bird, likely the extant emu, *Dromaius novaehollandiae* is represented by a partial distal tarsometatarsus and egg shell. The tarsometatarus is similar in size to extant *Dromaius novaehollandiae* specimens possessing gracile condylar articular ends, unlike that seen in more robust *Casuarius* and the even more robust and thickened condyles observed in the Upper Pleistocene giant bird *Genyornis.* The egg shell bears the typical rugose surface ornamentation typical of *Dromaius novaehollandiae.*

SQUAMATA

Varanidae Merrem, 1820

Varanus priscus (Owen, 1859)

Supplementary Figure 3 (d-f, i, m & n)

Referred material: QML1470. Osteoderm (and impression) QMF60001; teeth QMF59914, QMF57054, QMF59916; dorsal vertebrae QMF59991, QMF57091.

Varanus priscus is represented by isolated teeth, large osteoderms and vertebrae across several sites. The tooth, osteoderm and vertebrae are diagnostic as varanid in morphology and their very large size makes them indistinguishable from *V. priscus*[14]. Teeth are labio-lingually compressed (ziphodont), recurved, serrate with denticles and basal plicidentine[7, 14]. The sizes of the teeth are comparable to *V. priscus*, being much larger than *V. komodoensis*. The vermiform osteoderms are also diagnostic for varanids, with most osteoderms recovered from the sites being very large >15 mm long and outside the maximum size range of extant *V. komodoensis* but within the size range of *V. priscus* from the Eastern Darling Downs (Murray Darling Basin). The two dorsal vertebrae are morphologically and morphometrically similar to the type specimen of *V. priscus* (BMNH 32908c) and additional vertebrae referred to *V. priscus* in the Queensland Museum Geosciences collection.

Varanus sp. (large)

Supplementary Figure 3 (c, g, h, j & k, l)

Referred material: QML1470. Osteoderm QMF60002; teeth QMF59917, QMF59915; parietal bone QMF59913; sacral vertebra QMF57053.

A second species of giant *Varanus* is indicated by the presence of isolated teeth, a sacral vertebra, small osteoderms and a partial parietal bone. All elements are considerably smaller and morphologically distinct from those known of *V. priscus* and *V. komodoensis*. However the overall size of the elements is similar to that of *V. komodoensis.* In particular, the sacral differs by having a more circular cotyle and relatively smaller zygopophyses compared to that seen in extant *V. komodoensis*. QMF59913 is a parietal bone that has been split in half but preserves the right side, the channel for the pineal foramen, and the medial margin of the supratemporal fenestrae. It differs substantially from the parietal of *V. komodensis* and *V. priscus* with the angle between the anterior and posterior supratemporal bars being more acute than that seen in *V. komodoensis*[14], and lacking a central parietal ridge as seen in *V. priscus*. These differences observed suggest a morphologically distinct species of *Varanus*. The possibility of intermediate *Varanus* species between *V. komodoensis* and *V. priscus* being present in the Australian Pleistocene has been suggested previously[14]; however without an ontogentic series of *V. priscus* known it is difficult to exclude the possibility that these elements represent a juvenile *V. priscus* which would indicate significant morphological change through ontogeny.

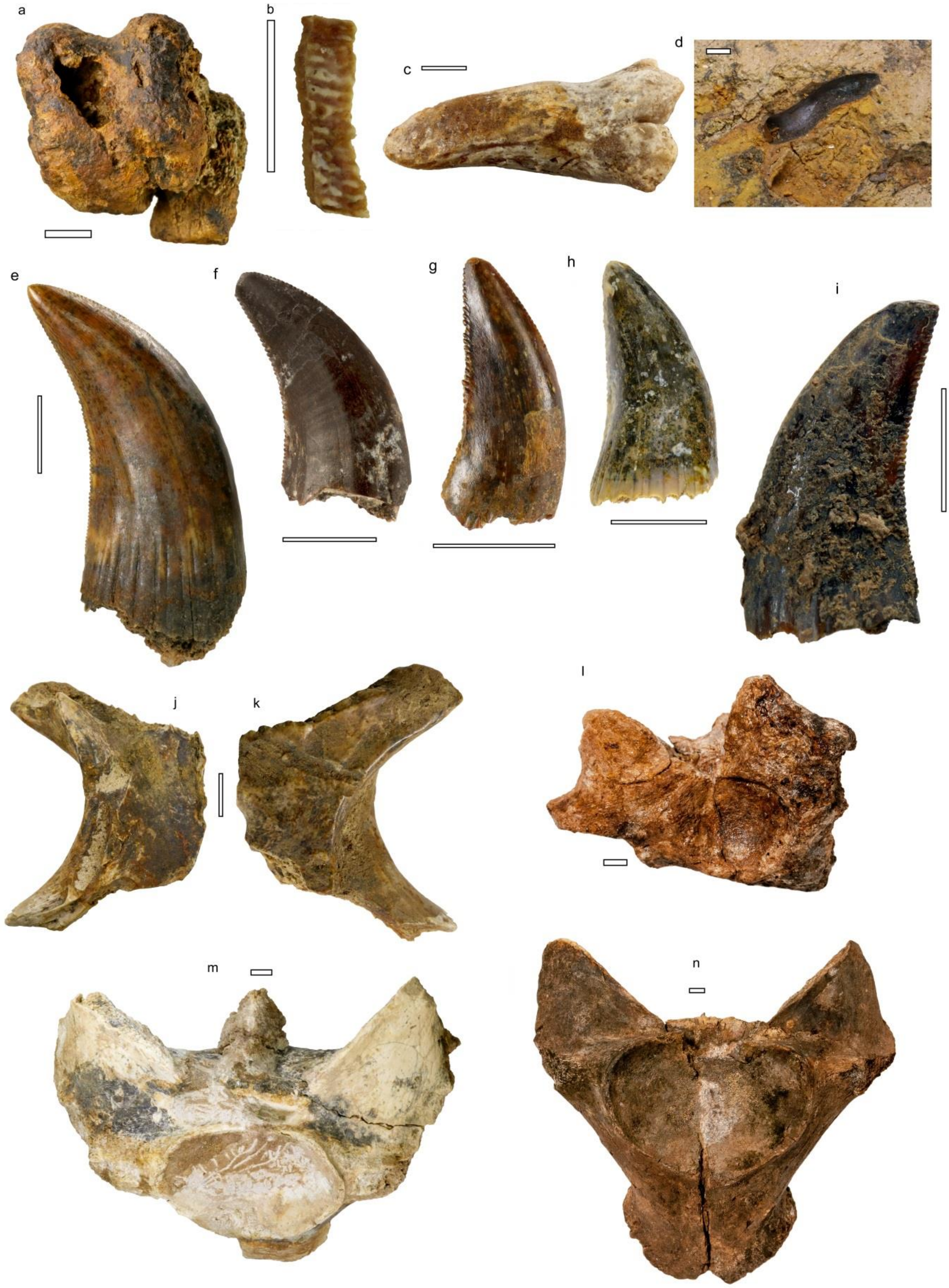

Supplementary Figure 3. Casuariidae and Varanidae. *Dromaius* sp. cf. *D. novaehollandiae* (a) distal tarsometatarsal QMF57167; (b) egg shell QMF60000. *Varanus* sp. (large) (c) osteoderm QMF60002. *Varanus priscus* (d) osteoderm (and impression) QMF60001. *Varanus* teeth (e–i): (e) *V. priscus* QMF59914, (f) *V. priscus* QMF57054, (g) *Varanus* sp. (large) QMF59917, (h) *Varanus* sp. (large) QMF59915, (i) *V. priscus* QMF59916. *Varanus* sp. (large) (j-l); (j+k) parietal bone (right side) QMF59913; (l) sacral vertebra QMF57053. *V. priscus* dorsal vertebra (m) QMF59991, (n) QMF57091. Scale bars equal 5 mm.

MAMMALIA

Order DIPROTODONTIA Owen, 1866

Macropodidae Gray, 1821

The most abundant and taxonomically diverse herbivores recovered from South Walker Creek sites are macropodines, in particular species previously within *Macropus*. We use the diagnostic molar morphology that have been used to define subgenera of *Macropus* [15]; however, to remain consistent with current taxonomy, we have elevated these subgenera to genus level[16]. Four species of macropodine kangaroos are currently recognised in the fauna: a species each of *Macropus, Notamacropus, Osphranter* and *Protemnodon,* as well as a species of a small-sized sthenurine. We recognise three extinct species that are possibly new or significant variants, including a giant species of *Macropus* with affinities to *Macropus Macropus pearsoni*; a giant species of *Notamacropus* distinct from *Notamacropus agilis siva* and a species of high-crowned *Protemnodon* that is similar in size to *Protemnodon brehus*. The extant *Osphranter rufus* and a small sthenurine are the rarest components of the macropodid fauna.

Macropodinae Gray, 1821

Notamacropus Dawson & Flannery 1985

Notamacropus sp. (giant)

Supplementary Figure 5 (a-i)

Referred material: QML1470. Mandible with R+L I_1- P_3-M_4 QMF59990; partial left dentary I_1-M_2 QMF59992; associated dentition and postcranial remains QMF57047 including RI^3 , RM_3, left calcaneum, right and left tibiae; LM^{2-3} QMF59994; RP^3 QMF59993.

Notomacropus sp. occurs across most sites and includes a near complete mandible (SW3-P), associated dentition and postcranial remains (SW9), along with isolated dentition (SW3, SWCC and Kemmis). QMF59990 and QMF59992 are mandibles possessing features seen in species of *Notomacropus*, including a lanceolate I_1 and vertical hypolophids that lack a vertical groove or notch[15]. Isolated lower molars also lack the vertical groove or notch or very tall hypolophids. These remains are comparable to the largest individuals assigned to *Notomacropus agilis siva*[17]; however, it is clearly different in P_3 morphology when compared to specimens from the Darling Downs (e.g. QMF4492). Of the largest species of *Notomacropus* QMF59994 is comparable to *N. agilis siva* due to it lacking a distinctive fore-link which is present in *N. thor* and not in *N. agilis siva*. QMF59993 is a RP^3 and possesses distinctive morphological features that cannot be firmly placed with a known taxon. Superficially the premolar is similar in morphology to *M. dryas*[18] and the exposed P^3 in *N. agilis siva* (QMF4541). Based on its size and in comparison to the P_3 available in QMF59990, it is likely that this P^3 (QMF59993) represents the same taxon. This premolar has been compared to extant and extinct species and does not match the morphology seen in species of *Protemnodon, Osphranter, Macropus, Troposodon, Wallabia, Petrogale, Thylogale, Dendrolagus, Dorcopsis, Dorcopsulus* or any species of sthenurine. On comparison with the lectotype of *Macropus dryas* (QMF3582), it shares a similar antero-posterior central

crest with one large and three small cuspules each with an accessory buccal vertical crest running to the base of the tooth, and presence of a posterolingual pocket and two large well-developed lingual margin cuspules. It differs by being larger; possessing distinct lingual cuspules and missing an antero-lingual cuspule; and a more tapered anterior margin and smaller postero-lingual basin. Together, this suite of specimens likely represents a species of giant *Notomacropus* at the larger size range of the genus and is as yet an unnamed taxon.

Osphranter sp. cf. *O. rufus* (Desmarest, 1822)
Supplementary Figure 5 (j)
Referred material: QML1470. RM_4 QMF59929; posterior half of $RM^{2 \text{ or } 3}$QMF59918.

A lower molar and partial upper molar represent a large species of *Osphranter* with very close morphology to that observed in the extant Red Kangaroo, *Osphranter rufus*. In molar dimensions the lower molar is just within the largest available specimens for comparison, which were all from male specimens. The molar bears distinctively high crowns with thick and unkinked fore and mid-links. The shallow lingual to buccal angle made by these straightened fore and mid-links, as seen in *O. rufus,* are not found in other similarly-sized species of *Macropus* bearing a posterior hypolophid groove, such as *M. titan* and *M. ferragus*. The shallower hypolophid groove in QMF59929 also differentiates the fossil from most specimens examined of these later extinct species. The fossil also falls within the lower size limit of these later extinct species. On inspection of the macropodid collections at Queensland Museum, three fossil dentary fragments (QMF9405, QMF9407 and QMF9408) from the same locality on the Eastern Darling Downs, bear near identical morphology to QMF59929. These combined records are the first identifications of Pleistocene records for the Red Kangaroo (*O. rufus*) in Queensland[19]. The presence of *O. rufus* at South Walker Creek is not surprising because it has modern collection records from approximately 150kms to the west.

Macropus Shaw, 1790
Macropus sp. (giant)
Supplementary Figure 5 (l-aa)
Referred material: QML1470. LI_1 QM59921; LI_1 QMF59992; LI^3 QMF57163; $LM_{2\text{-}3}$ QMF59928; associated hind limb elements, calcaneum, astragali, metatarsal IV, associated phalanges IV.I, II & III, tibiae and associated distal epiphyses QMF57039; IV.III (ungual) QMF57161; left femora QMF57086, QMF57038; right humerus QMF57036; associated tibiae, epiphyses and astragali QM59997; articulated metatarsals V and VI QMF59911.

Two isolated distal tips from left lower incisors, an isolated left I^3, and two longitudinally split molars (M_{2+3}) represent a very large species of macropodine referrable to *Macropus* based on: 1) the presence of enamel covering the ventro- and lingual faces of the lower incisors, extending up to well above halfway up the lingual face; and 2) posterior surface of the hypolophid rounded and possessing a vertical groove emanating from just below the hypoconid crest[15, 17, 20]. On comparison to similarly-sized species of *Macropus* the fossils resemble *M. pearsoni,* such as the very thick ventro-lingual enamel covering, development of the hypolophid

groove into a steep, deep groove with a sharp lingual (buccal) margin and a tall anterior cingulum on lower molars[15, 20]. In addition to these features, the fossils also share with *M. pearsoni* and *M. pan* a near-vertical and sharp post-protocristid that runs down the protolophid to the anterior termination of a very high and kinked cristid obliqua. This feature is developed to an extreme in *M. pan* and *M. pearsoni*. As the dental remains are fragmentary this leaves doubt that a definitive identification to previously described taxa is possible.

Postcranial elements representing a very large macropodine may represent the same taxon. At least ten tibiae representing a giant macropodine are among the most common macropodid element recovered from the South Walker Creek sites. The tibiae are very long and gracile elements with a straight diaphysis in lateral aspect. The condylar surface of the proximal epiphysis is flat, near horizontal and possesses a distinct intracondylar eminence. The anterior tibial crest is antero-posteriorly narrow, gently convex in profile, and extends distally, tapering into the diaphysis proximal to the mid-shaft. The articular surface of the distal epiphysis is near perpendicular to the antero-posterior plane of the anterior tibial crest. Based on characteristics of the tibiae established for macropodids[21] these tibiae share features with both macropodines and sthenurines, however, the predominance of macropodine characters over sthenurine characters favours a macropodine placement. In particular a macropodine placement is supported by the combination of a near straight diaphysis, and the near horizontal proximal and perpendicular distal epiphyseal articular surfaces. The distally elongate and smoothly convex anterior tibial crest is a feature present in sthenurines[21, 22], although this feature has been observed in *Macropus rufus*[21]. On comparison to available very large macropodines with associated postcranial remains (including *Macropus giganteus, M. rufus*, *M. giganteus titan, M. ferragus, Protemnodon anak* and *P. snewini*) these tibia differ most clearly on their overall smaller size (Supplementary Figures 4 and 5) , stockier proportion and presence of an abrupt end to the distal margin of the anterior tibial crest. A single morphologically very similar, but proximo-distally shorter and more robust, tibia occurs within the Pliocene Chinchilla Sands Local Fauna (WC5928). WC5928 is the only known example of this tibia morphotype found outside of the South Walker Creek sites. WC = Wilkinson Collection within the Queensland Museum collection.

Fibulae, calcaneum, astragali, metatarsal IVs, digit IV phalanges and two digit IV unguals represent the giant *Macropus* from the South Walker Creek fauna. All exhibit typical macropodine characteristics[21]. The digit IV ungual (QMF57039 & QMF57161) deserves further consideration due to its unique morphology. It is straight, triangular in both longitudinal and cross-sectional, and has a sharp tapered distal point. The dorso-lateral sides of the main body are straight with the distal claw tip curved slightly dorsally, creating a unique recurvirostral-like shape. Morphologically similar unguals are exceptionally rare in the literature, having only been published from the Quanbun fauna[23] and figured twice in comparison to other macropodid taxa[23, 24]. These unguals have been tenuously assigned to *Macropus pan* on the basis that unguals of this morphology represent the largest species of *Macropus* from their respective faunas (Quanbun and Chinchilla) and that the largest *Macropus* known from dentition at these same sites is *M. pan*[23]. Inspection of the Chinchilla Sands Local Fauna collections at Queensland Museum confirm that this ungual morphotype is present, albeit

uncommon, and does represent the largest macropodine ungual recorded. Therefore, assignment of this ungual to *M. pan* at Chinchilla does seem warranted even though no direct associations are available. The tibia mentioned above, from the Chinchilla Sands Fauna, also represents the largest macropodine tibia recovered so far from Chinchilla. This tibia shares closest morphological features with the taxon from South Walker Creek, albeit a smaller, more robust and considerably geologically older taxon.

Macropus pearsoni, a large Pleistocene species, shares similar, but further specialised, dental characteristics with *M. pan*[15, 20]. Both species have been previously considered members of a separate genus or subgenus *Fissuridon*[20, 25]. Based on the close dental morphological similarities seen between *M. pan* and *M. pearsoni*, it seems likely that if the postcranial elements referred to *M. pan* are indeed correctly assigned, the postcranial morphology of *M. pearsoni* would bear closest resemblance to that of *M. pan.* However, no post-cranial remains (partial or otherwise) with similar morphology and size to those found at SWC are known from any other Pleistocene site or localities from which *M. pearsoni* is presently known. Post-cranial remains of macropodines from these other Queensland Pleistocene localities are much smaller.

As the fragmentary dental remains found at South Walker Creek are similar in features to *M. pearsoni*, and that the postcranial remains bear closest morphological resemblance to postcranial material assigned to *M. pan*, we regard the most likely candidate for the giant macropodine both in dentition and postcranial remains at South Walker Creek to be closely related to *M. pearsoni* and *M. pan*. However, we refrain from formally allocating these remains to either taxon due to the reasons mentioned above and because we cannot rule out an as yet undescribed species.

Protemnodon Owen, 1874

Protemnodon sp. nov?

Supplementary Figure 5 (ab-an)

Referred material: QML1470. Isolated metatarsal IV QMF57037; an associated juvenile with RI_1, LP^2-M^2, LP^2, LP^3, thoracic vertebrae, left femur, left fibula, left and right tibiae, tibial epiphyses, metatarsal IV-IV.III, mtV-V.I and III.I QMF57165; associated adult with LI_1, RI^3, LP^3, a left mandible with P_3-M_4, left pelvis, left proximal femur QMF57035; left proximal femur QMF57127; P^3 QMF59933.

The best preserved macropodid dentition so far recovered from South Walker Creek includes teeth from two individuals, an adult and a sub-adult, from QML1470 (SW9). These two individuals also possess associated postcranial remains that are either semi-articulated or closely associated in the site. We assign these adult and sub-adult dental remains to their respective post-cranial associations. On comparison with species of *Protemnodon*[26, 27, 28, 29, 30, 31, 32, 33], this taxon is larger than *P. snewni, P. anak, P. chinchillaensis, P. devisi*, *P. otibanda* and *P. nombe*. It is smaller than *P. roechus*, leaving *P. hopei* and *P. brehus* for comparison. The lower dentition is higher crowned than *P. hopei* but shares closest morphological features with *P. brehus*. The South Walker Creek taxon differs in upper molar morphology to *P. brehus* by possessing less developed central and buccal crests and better developed anterior cingulum on P^2. dP^3 is lower crowned with a distinct mid-link and buccal protoloph-hypoloph link, and broader posterior cingulum. P^3 is higher crowned with anterior cingulum

present and a deep posterior fosset. Upper molars are distinctly higher crowned than referred specimens of *P. brehus* from the eastern Darling Downs (e.g. QMF4954, QMF4947 and QMF12505). Posterior unworn crown heights of M^2 (11.24mm) and M^3 (13.03mm) in QMF 57165 are taller than measured unworn molars of the Darling Downs *P. brehus* specimens (e.g. QMF 4954, M^2 (7.66mm) and M^3 (9.04mm) and QMF4947 M^3 (8.84mm)). $M^{1\text{-}3}$ possesses prominent buccal links between protoloph and hypoloph with a narrower anterior cingulum. Overall the SWC *Protemnodon* is either a new taxon or a higher crowned northern variant of *P. brehus*.

Sthenurinae Glauert, 1926
sthenurine indeterminate
Supplementary Figure 5 (k)
Referred material: QML1470. Partial RM^{1or2} QMF59999.

A fragment of an anterior upper right molar represents the only definitive evidence of the Sthenurinae within the Pleistocene South Walker Creek fauna. It is the second record of sthenurines within the Fitzroy River Catchment, the only other evidence being an unpublished partial molar from a Middle Pleistocene cave deposit at Mt. Etna[12] in the lower reaches of the Fitzroy River Basin. QMF59999 preserves the antero-buccal corner of the upper molar, including the paracone, protoloph and anterior cingulum. The combination of features of this molar that identify it as a species of sthenurine include; a low crowned protoloph with the anterior slope possessing crenulations (3, possibly 4 crenulations); a broad, low anterior cingulum; and a distinct indentation and groove located at the junction of the preparacrista and the anterior cingulum. On comparison with sthenurines[34] this taxon is a very small species, similar in size to *Simosthenurus andersoni* and *Procoptodon gilli*. There are no additional features that assist with identifying QMF59999 further than a species of small-sized sthenurine.

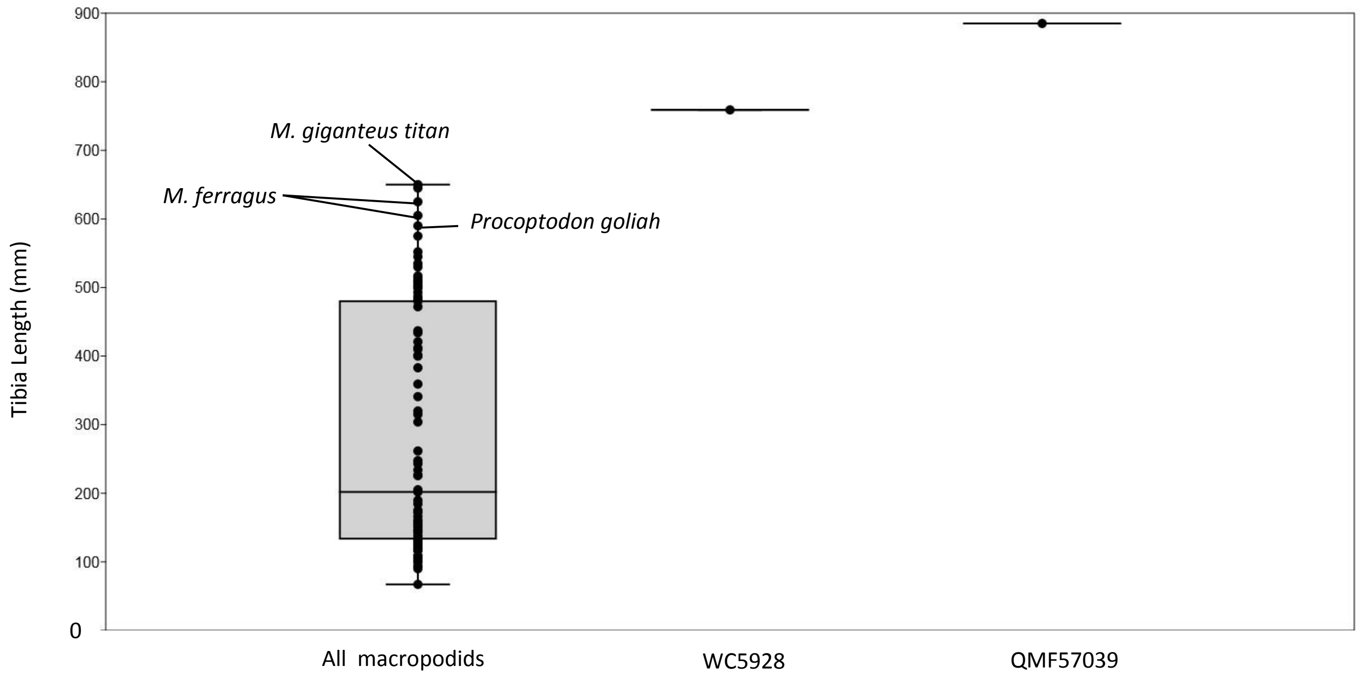

Supplementary Figure 4. Box plots of tibia lengths (mm) for macropodids from published records of museum specimens[35, 36] (left), *Macropus* ?*pan* WC 5928 (centre) and the South Walker Creek *Macropus* sp. (giant) QMF57039 (right). Source data are provided as a Source Data file.

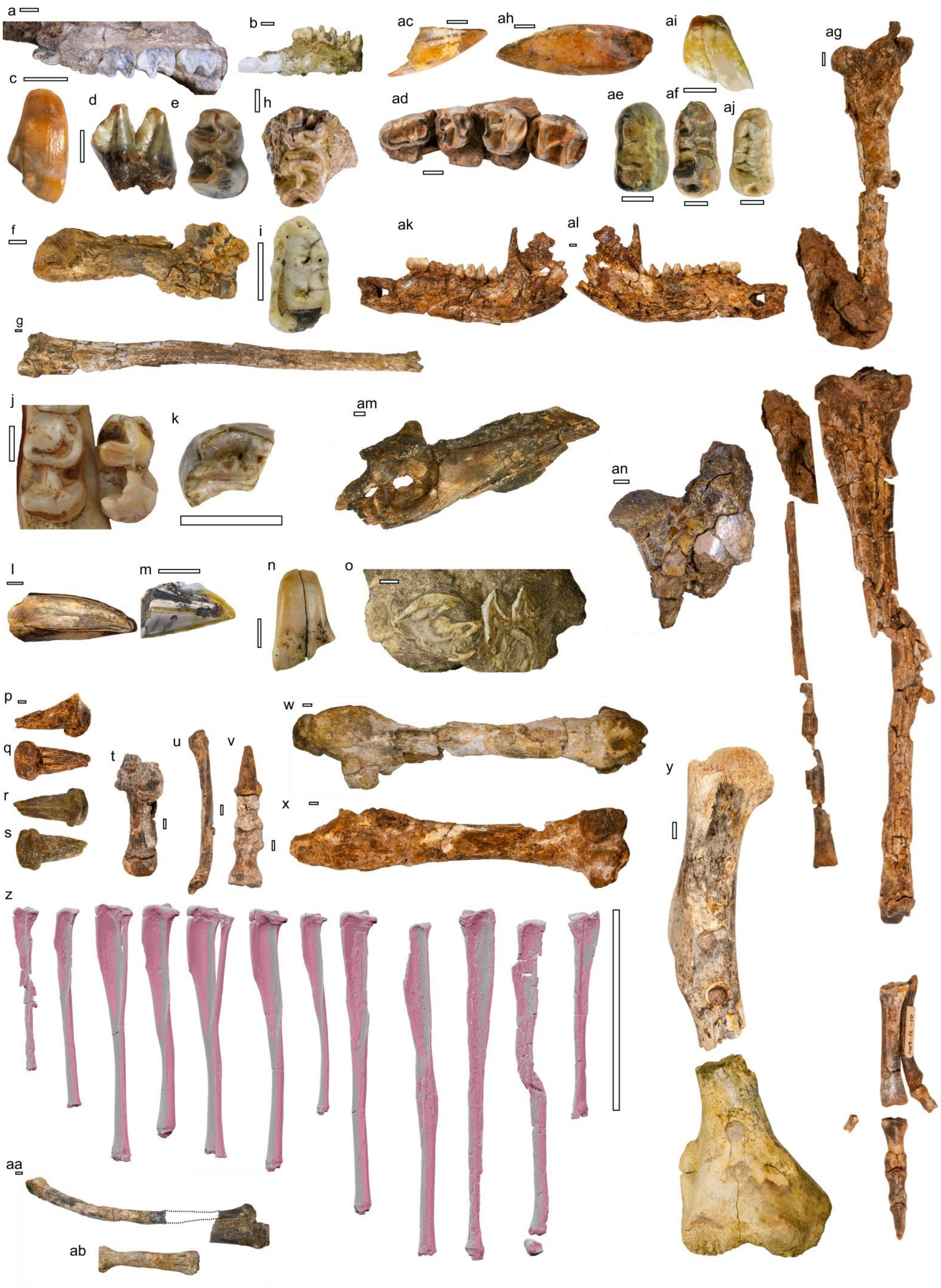
a
b
ac
ah
ai
ag
c
d
e
h
ad
ae
af
aj
f
i
ak
al
g
j
k
am
an
l
m
n
o
p
q
r
s
t
u
v
w
x
y
z
aa
ab

(Over Page) Supplementary Figure 5. Macropodidae. *Notamacropus* sp. (a–i); (a) left dentary showing P_3-M_4 of mandible QMF59990; (b) partial left dentary I_1-M_2 QMF59992; (c–g) associated dentition and postcranial remains of QMF57047; (c) RI^3, (d–e) RM_3, (f) left calcaneum, (g) right tibia; (h) $LM^{2\text{-}3}$ QMF59994; (i) RP^3 QMF59993. *Osphranter* sp. cf. *O. rufus* (j) RM_4 extant *O. rufus* QMJ4452 (left) and RM_4 fossil QMF59929 (right). Sthenurinae (k) Sthenurine indeterminate RM^{1or2} QMF59999. *Macropus* sp. (l) LI_1 QM57039; (m) LI_1 QMF59921; (n) LI^3 QMF57163; (o) $LM_{2\text{-}3}$ QMF59928; (p–q) associated hind limb elements IV.III (ungual) in lateral and ventral views, (t) calcaneum, (u) metatarsal IV, (v) associated phalanges IV.I, II & III; (r-s) IV.III (ungual) in dorsal and ventral views QMF57161; (w) left femur QMF57086; (x) left femur QMF57038; (y) right humerus QMF57036; (z) 3-D models generated through CT scans, aligned together of macropodids for comparison to the giant *Macropus* sp. Left – Right; *Protemnodon* sp. QMF57165; *Protemnodon snewini* QMF9075; *Macropus ferragus* MVP28290; *Protemnodon* sp. QMF14.624, *Macropus titan* QMF12262; *Macropus titan* QMF3298; *Macropus giganteus* (modern); *Macropus ?pan* (Chinchilla) WC5928; *Macropus* sp. QM59997, QMF57039 (right and left tibia); *Sthenurus stirlingi* MVP150275. *Macropus* sp. (giant) (aa) articulated metatarsal V and VI QMF59911. *Protemnodon* sp. (ab) isolated metatarsal IV QMF57037; an associated juvenile (ac) RI_1, (ad) LP^2-M^2, (ae) LP^2, (af) LP^3, (ag) femur, fibula, tibia, metatarsal IV-IV.III, mtV-V.I and III.I QMF57165; associated adult (ah) LI_1, (ai) RI^3, (aj) LP^3, (ak+al) left mandible with P_3-M_4, (am) left pelvis, (an) left femur QMF57035. Scale bars equal 5 mm (a–y, aa–an); 500 mm (z).

Suborder VOMBATIFORMES Woodburne, 1984

Palorchestidae Owen, 1874

Palorchestes Owen, 1874

Palorchestes sp. nov?

Supplementary Figure 6 (a-c)

Referred material: QML1470. Left I^3 and M^2 QMF57059.

Two teeth referrable to *Palorchestes* were found together within QML1470 (SW9), both similarly worn and coming from the left upper dentition, we therefore regard them as associated. The LI^3 is heavily worn and difficult to directly compare with species of *Palorchestes* due to few I^3s being known for described *Palorchestes* species[37]. The I^3 is larger than the I^3 preserved in *P. parvus* (QMF789) but is morphologically similar. It differs by possessing thicker enamel on the antero-buccal surface. The tooth is split medially, but would have been antero-posteriorly broader than QMF789. The I^3 of *P. azael* is unknown so it was not possible to be directly compared; however, the illustrated rostrum[37] of *P. azael* (QMF3837) indicates an I^3 alveolus that would have received an incisor that was proportionately broader than that seen in *P. parvus* or *P. painei*. However, *P. azael* is much larger than *P. parvus*, and the I^3 of QMF57059 would have therefore been much smaller than that expected in *P. azael*. Similarly, the LM^2 is worn and is much smaller than the Upper Pleistocene *P. azael* specimens (e.g. QMF772 & QMF33510). It is, however, more morphologically similar to *P. azael* than it is to Palorchestes closer in size to QMF57059 such as *P. parvus*[37, 38, 39, 40, 41, 42, 43, 44, 45]. Even though QMF57059 is missing the lingual third of the molar and is heavily worn, there are a number of features that differentiate it from all other species of described *Palorchestes*. The projected unworn tooth crown height along with a steeper tooth wear gradient across the lophs is greater than that seen in smaller species of *Palorchestes* but similar to the much larger *P. azael*. An accessory buccal anterior link on the anterior cingulum, buccal interloph cuspule, faint protoloph crenulations and posterobuccal vertical groove of the hypoloph provides a combination of features not seen in species so far described. Due to the poor

representation of this taxon a firm species-level allocation cannot be made; however, it is likely that it represents either a new species or a significantly small-sized variant of *P. azael.*

Thylacoleonidae Gill, 1872

Thylacoleo Owen, 1859

Thylacoleo sp.

Supplementary Figure 6 (d-i)

Referred material: QML1470. Associated *Thylacoleo* postcranial remains including a left fibula, left metacarpal III, phalanx, ?left V.I phalanx QM59910; isolated phalanx QMF59912.

Although the distinctive dentition of *Thylacoleo* has yet to be recovered from Quaternary sites within the South Walker Creek system this taxon is represented at QML1470 (SW9) by several associated postcranial elements and rare, but distinctive bite-marked bones. The elements recovered from this individual include a left fibula and left manual elements. On comparison with described skeletal elements of *Thylacoleo carnifex*[46, 47, 48, 49], and additional comparison with photographs of the fibula, pes and manual elements of *T. carnifex* specimens (kindly provided by Aaron Camens, Liz Reed and Steve Bourne, respectively), it is clear that these elements are referrable to *Thylacoleo*. The fibula is a thin, elongate limb, with expanded proximal epiphysis that possesses a bevelled proximal articular end with flattened femoral and tibial facets as illustrated here[48]. The metacarpals and phalanges are gracile and elongate elements, easily distinguished from macropodid, vombatid and diprotodontid pedal and manual elements. They possess narrow mid-shafts that bend dorsally with flared proximal and distal articular ends. The fossils are directly comparable to those illustrated here[46, 47]. Based on their similar size and the Upper Pleistocene age of the South Walker Creek deposits, it is likely that the species is *Thylacoleo carnifex*. However, without dental remains to confirm this identification, we will refer these fossils to *Thylacoleo* sp.

Vombatidae Burnett, 1830

Sedophascolomys Louys, 2015

Sedophascolomys sp. cf. *S. medius* (Owen, 1872)

Supplementary Figure 6 (j-o)

Referred material: QML1470. RM^{3-4} QMF59934; associated right and left I_1s QMF57069.

The lower right and left incisors, along with isolated pedal phalanges, so far represent a single large vombatid individual, smaller in size than the largest known vombatid species *Phascolonus gigas.* The specimens are both larger and morphologically different to members of the extant genera, *Lasiorhinus* and *Vombatus.* Following the traits set out by Dawson[50] for the lower incisors of extant and extinct wombat taxa, these fossils share traits with *Phascolomys medius*, now established within its own genus, *Sedophascolomys*[51]*.* In cross-sectional shape the incisors are ovoid with the height of the incisor slightly taller than wide. Enamel is distributed around the ventral half of the tooth with fine longitudinal striations evident. Both incisors preserve partial wear facets from the upper incisors. These facets indicate an elongate wear area, similar to

that seen in specimens assigned to *S. medius*. The tooth shafts are straight, lacking any dorso-ventral curvature along their length. This is a unique trait and unlike any of the incisors observed in well-preserved specimens.

On closer inspection of comparative specimens available for this study and through X-ray computed tomographic scanning of several specimens (Supplementary Figure 6, p-r), the incisors of *S. medius* show limited or no bending along their antero-posterior length. This traits is here considered to be an autapomorphy of *Sedophascolomys*, although it will require further investigation with other fossil vombatid taxa where lower incisors are known but not entirely visible due to preservation.

Based on the above features, along with the very close size to comparative specimens at the Queensland Museum collection from the Darling Downs and Chinchilla sites of Queensland, it is most likely that this wombat represents the youngest fossil occurrence for *Sedophascolomys* and a Pleistocene range extension into northern Australia. Since the genus is monotypic we tentatively allocate these specimens to the Plio-Pleistocene species, *S. medius*, until further material is available.

Phascolonus Owen, 1872

Phascolonus gigas (Owen, 1858)

Supplementary Figure 6 (p-v)

Referred material: QML1470. An associated specimen including right and left femora, pelvis and articulated sacral and caudal vertebrae, and associated teeth including RI^1, RI_1 fragment, $RM^{3\text{ or }4}$, RM_4, LM_2, LM_3 and LM_4 QMF57065.

Phascolonus gigas is represented at QML1470 (SW9) by a single partial skeleton including dentition, hind appendicular elements along with a large pelvis with articulated caudal vertebrae. Isolated ribs and pedal elements likely belong to this same individual, but comparative material of *P. gigas* was difficult to attain for this preliminary identification. *P. gigas* has been identified on the basis of the autapomorphic dorso-ventrally compressed and medio-laterally broad upper incisors[50]; large hypselodont molars and morphologically similar postcranial elements as seen in specimens recovered from Lake Callabonna[52] and via photography provided to us by A. Camens (*pers comm.,* 2014) of cast specimens lodged within the South Australian Museum.

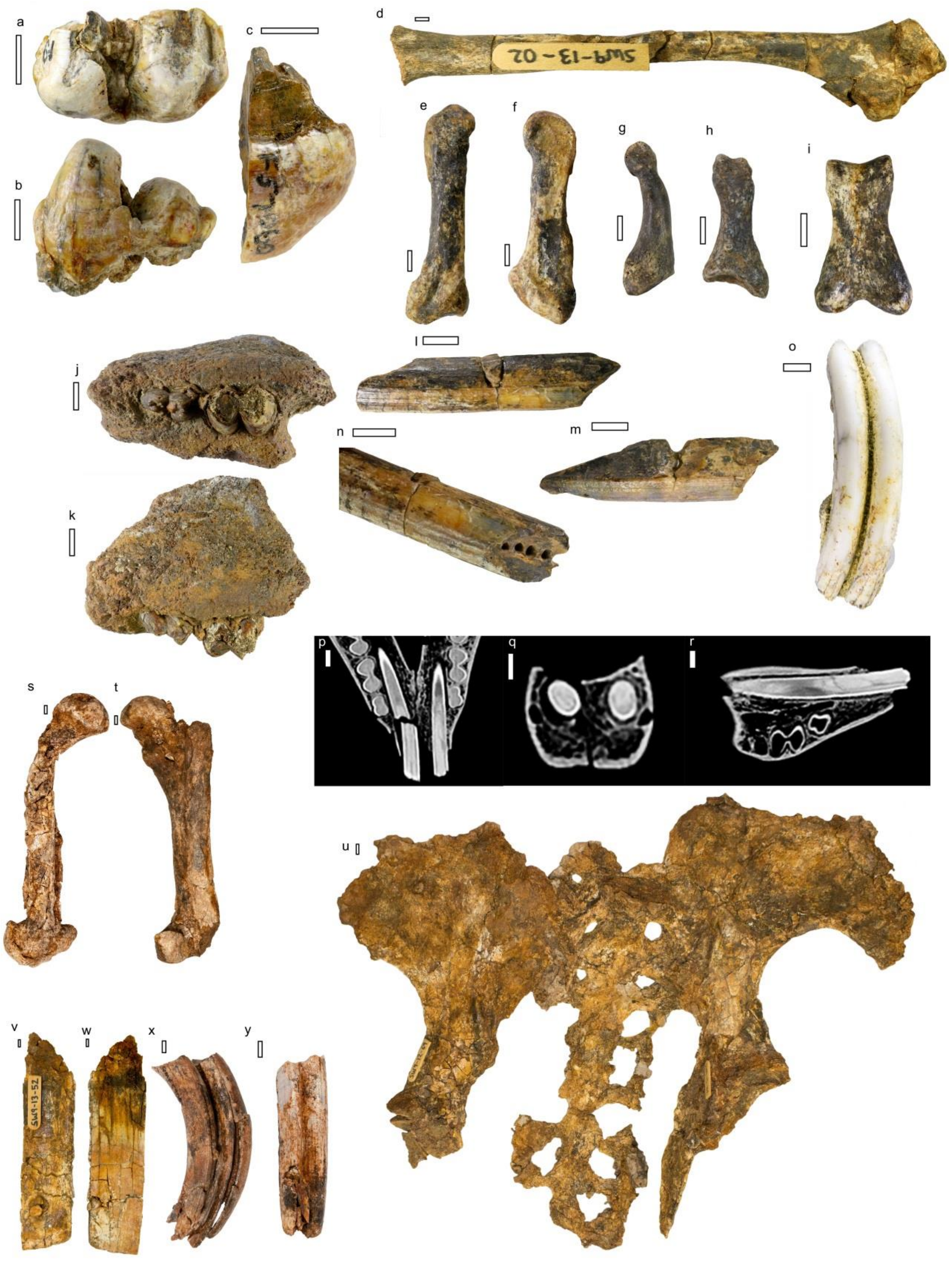

Supplementary Figure 6. Palorchestidae, Thylacoleonidae and Vombatidae. Associated *Palorchestes* sp. teeth (a–b) LM^2, (c) LI^3 QMF57059. *Thylacoleo* sp. associated postcranial remains; (d) left fibula, (e–f) left metacarpal III, (g) phalanx, (h) ?left V.I phalanx QM59910; (i) phalanx QMF59912. *Sedophascolomys* sp. cf. *S. medius* (j–k) RM$^{3\text{-}4}$ QMF59934; (l–n) associated right and left I_1s showing drill position for uranium-series samples QMF57069 (o); LM$_4$ QMF59920. *Sedophascolomys medius* mandible CT scans (p-r) showing the straight lower incisor with ventral enamel and dorso-ventrally oblong circular cross-section, QMF12493. *Phascolonus gigas* associated remains (s-y) including (s) right and (t) left femora, (u) pelvis with articulated sacral and caudal vertebrae, (v-w) RI^1, (x) RM^{3or4} and (y) RM$_4$ QMF57065. Scale bars equal 5 mm (a–r, v–y) and 10 mm (s, t + u).

Diprotodontidae Owen, 1838

Diprotodon Owen, 1838

Diprotodon optatum Owen, 1838

Supplementary Figure 7 (a-g) & Supplementary Table 2

Referred material: QML1470. An associated small-sized sub-adult with skull, mandible, pelvis, tibia shard and pedal elements QMF54689; an associated small-sized old individual with right dentary, skull, cervical vertebra, scapula and femur QMF59996; an isolated large skull QMF57172; astragulus QMF57066.

The cranial, incisor, premolar and molar morphology along with postcranial elements are indistinguishable from specimens referred to as *D. optatum*[53, 54, 55, 56, 57] and those compared directly; therefore we refer all of these specimens to *D. optatum*. However, the specimens from QML1470 (SW9) are worthy of further description. Two individuals are presently recognised within the prepared portion of fossil collection, a small-sized sub-adult large form and a small-sized, small form old individual. Both individuals are very small in overall size being at the minimum size range for *D. optatum* dentaries as described by Price[55]. The dentary depth to width of QMF54689 is 59.5 mm / 45.2 mm and of QMF59996 is 72 mm / 48 mm which is within the smallest range figured here[55]. However, on the basis of molar size QMF54689, the sub-adult, plots within the largest of the large form of Price[55] whilst QMF59996 plots within the small form size range (Supplementary Table 2). The sub-adult individual exhibits a large degree of wear on M_{1-2}, whilst the old individual has a fully erupted dental arcade that exhibits extreme levels of wear on all teeth. QMF54689 represents a sub-adult large morph individual (e.g. a male) and QMF59996 represents a very old small morph (e.g. a female), yet both individuals are at the minimum overall size for *Diprotodon optatum* defined by Price[55]. We conjecture that these specimens represent a population of *Diprotodon* that has undergone some degree of body-size reduction (dwarfing), or is a population exhibiting a small body-size variant not usual for Upper Pleistocene populations typical of southern Australia[56].

Supplementary Table 2. Dental measurements of *Diprotodon optatum* from South Walker Creek and inferred size form based on comparison to measurements in Price[55].

	**M_1**	**M_2**	**M_3**	**M_4**	**M^1**	**M^2**	**M^3**	**M^4**	**Size**
	L x PW<br>Area mm^2	L x PW<br>Area mm^2	L x PW<br>Area mm^2	L x PW<br>Area mm^2	L x PW<br>Area mm^2	L x PW<br>Area mm^2	L x PW<br>Area mm^2	L x PW<br>Area mm^2	Large or<br>Small Form[55]
QMF54689	37.88 x 25.9 mm<br>981mm^2	45.99 x 31.8mm<br>1463 mm^2	56.96 x 40.09mm<br>2283 mm^2	erupting					Large
QMF59996	missing	heavily worn	48.5x ?mm	54.72 x 33.76mm<br>1849 mm^2	32 x 34mm<br>1088 mm^2	40.4 x ?mm	45.2 x ?mm	42.3 x ?	Small
QMF57172					32.97 x 31.48mm<br>1037mm^2	42.33 x 38.3mm<br>1621mm^2	50.82 x 41.27mm<br>2097mm^2	48.82 x 44.78mm<br>2186mm^2	Small

Zygomaturus Owen, 1858

Zygomaturus trilobus (Macleay, 1857)

Supplementary Figure 7 (i)

Referred material: Homevale Station. Mandible QMF11136.

A near complete mandible preserving the right and left dentition from I_1-M_4 represents *Zygomaturus trilobus*. This specimen possesses the typical morphological traits of *Z. trilobus* including molars that are broad and square in occlusal outline, crescentic lophids with wide u-shaped median valleys, a small triangular P_3 and parallel short incisors.

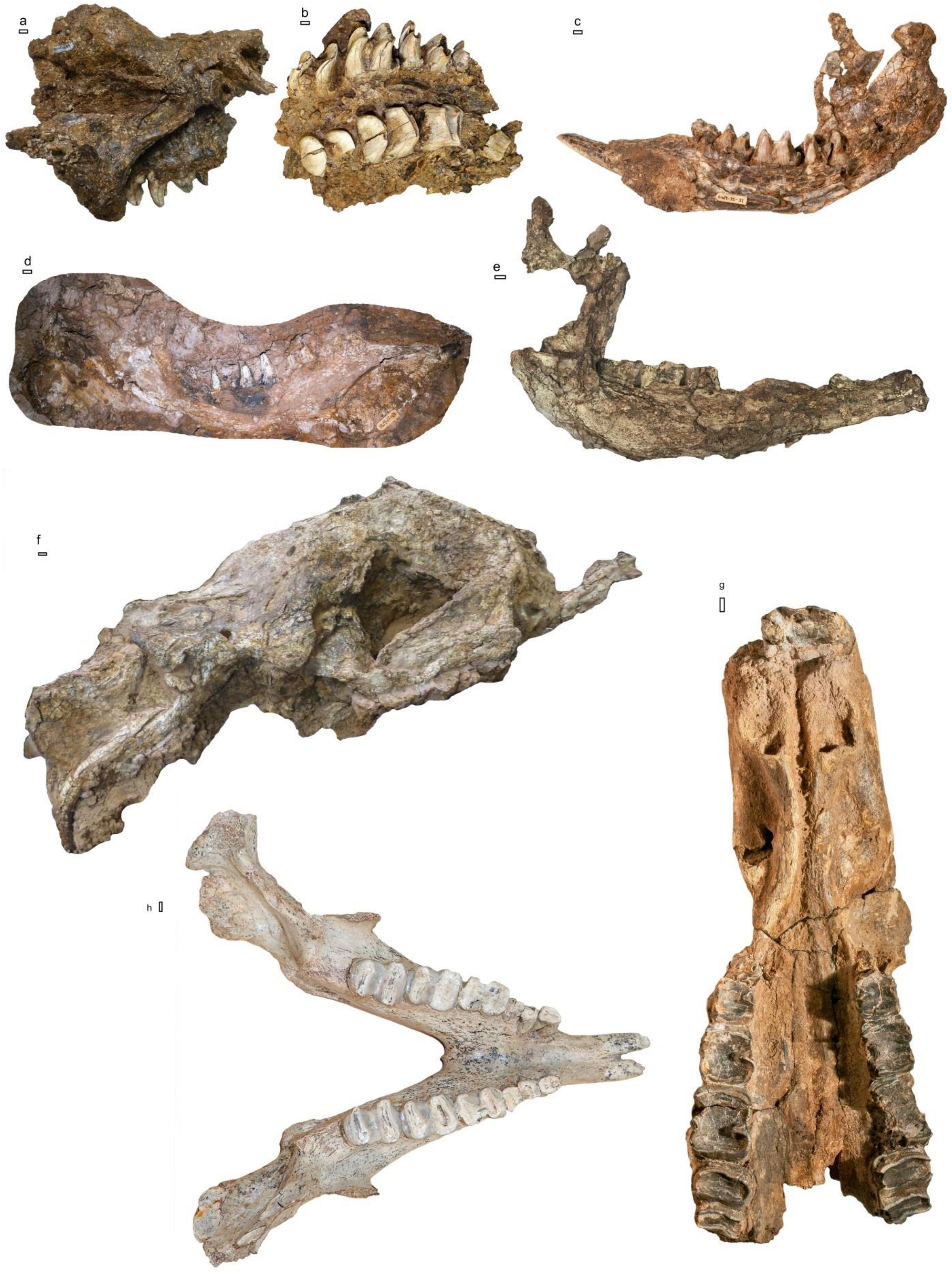

Supplementary Figure 7. Diprotodontidae. *Diprotodon optatum* (a–d) an associated skull and mandible; (a) skull in lateral and (b) oblique ventral views; (c–d) mandible showing (c) prepared right dentary and (d) unprepared left dentary QMF54689; (e) associated right dentary and (f) skull QMF59996; (g) skull in occlusal view QMF57172. *Zygomaturus trilobus* (Homevale Station) (h) mandible QMF11136. Scale bars equal 10 mm.

Supplementary Note 2: Faunal Associations

Scott A. Hocknull & Rochelle A. Lawrence

Articulated and semi-articulated remains were recorded during excavations and preparation. Association of remains was determined through a process using: 1) identical taxonomic identification, 2) observations of relationship within the excavation, 3) assessment of element duplication, 4) establishing the minimum number of individuals, 5) determining the proximity of any element to another sequential element within a body part, and 5) determining re-articulation fit for sequential associated elements.

Supplementary Table 3. Faunal Associations observed per site and per taxon. Numbering indicates the minimum number of individuals (MNI) recorded from each site. > = many isolated specimens (MNI not known). Abbreviations: Art = Articulated; Sem = Semi-articulated; Ass = Associated; Iso = Isolated; Trace = trace fossil; bm = bone modification (bite/cut marks); c = coprolite.

QML1470 (SW9)

Taxon	Art	Sem	Ass	Iso	Trace
Crocodile					bm
Pallimnarchus sp.				>	
Crocodylus sp. cf. *C. porosus*				>	
Varanus priscus				1	
Varanus sp. (large)				1	
Thylacoleo sp.			1		bm
Phascolonus gigas	1				
Sedophascolomys sp. cf. *S. medius*			1		
Protemnodon sp. nov?		2			
Macropus sp. (giant)		1	2	1	
Notamacropus sp. (giant)			1		
Palorchestes sp. nov?			1		
Diprotodon optatum			2		
Dromaius sp. cf. *D. novaehollandiae*					shell
Velesunio wilsoni	2	1		>	
Corbiculidae				2	
Arthropoda			>	>	

QML1470 (SW3)

Taxon	Art	Sem	Ass	Iso	Trace

				*	
Crocodile					bm/c
Pallimnarchus sp.				>	
Crocodylus sp. cf. *C. porosus*				>	
'Quinkana' sp.				1	
Varanus sp. (large)				1	
Varanus priscus				1	
Sedophascolomys sp. cf. *S. medius*				1	
Protemnodon sp. nov?			1	2	
Macropus sp. (giant)	1		1	1	
Notamacropus sp. (giant)	1			1	
Osphranter sp. cf. *O. rufus*				2	
Diprotodon optatum				2	
Dromaius sp. cf. *D. novaehollandiae*				1	

QML1470 (SWJ)

Taxon	Art	Sem	Ass	Iso *	Trace
Pallimnarchus sp.				1	
'Quinkana' sp.				1	
Varanus priscus				1	
Notamacropus sp. (giant)				1?	
Macropus sp. (giant)				1	

QML1470 (SWCC)

Taxon	Art	Sem	Ass	Iso *	Trace
Pallimnarchus sp.				>	
Crocodylus sp. cf. *C. porosus*				1	
'Quinkana' sp.				1	
Varanus priscus				1	
Protemnodon sp. nov?				1	
Macropus sp. (giant)			1		
Notamacropus sp. (giant)				1	

sthenurine				1	

Supplementary Note 3: Body mass estimation for *Macropus* sp. (giant)

Scott A. Hocknull

Macropus sp. (giant) left femur (QMF57038) mid-shaft circumference = 160mm

Mass estimate of *Macropus* sp. from equation 2 of Helgen et al 2006[58]

$$Mest = 1.0146*10^{[2.5932\ \log10(c) - 3.2842]}$$

$$Mest = 274.0431\text{kg}$$

Supplementary Note 4: Stratigraphic and Sedimentological description of QML1470 sites.

Scott A. Hocknull & Rochelle A. Lawrence

The fossil sites described herein are located along the eroded gully and rill systems of Walker Creek, nearby the South Walker Creek Mine (Latitude -21.708883° ; Longitude 148.349112°). Walker Creek is a headwater tributary of the Isaac River system that drains the northern section of the Fitzroy River catchment. The sites are located approximately 30km downstream of Lake Elphinstone. Several tributaries run in a north to southeast and southwest direction draining a northern headwater catchment created between the western Carborough Range, northern Pisgah (and Connors) Range and eastern Balaclava and Blue Mountains. Quaternary vertebrate fossils have been collected from the intervening valley system, derived from three separate areas along Walker Creek, Kemmis Creek and Homevale National Park. This system drains into the Bee Creek and Funnel Creek systems that enter the Isaac River. Funnel Creek formed a component of a previous Fitzroy River catchment-wide assessment of Quaternary alluvial sedimentation[59]. The fossil deposits presented here are derived entirely from the South Walker Creek sites; however, discussion of faunal remains will include those other sites in this headwater catchment, including fauna from Kemmis Creek and Homevale National Park.

Based on the current extent of surveys undertaken along Walker Creek the fossil sites are localised to short stretches of two meanders presently eroding Quaternary-aged alluvial deposits. The meanders are bedrock-controlled, with the basement of the creek system and alluvial deposits wedged upon the downward slope of a locally dipping Permo-Triassic Rangal and Fort Cooper Coal Measures and Triassic Rewan Group.

Sediments preserving the Quaternary fossils range from singular matrix-supported muddy cobble beds (~50cm thick) through fine-grained sandy muds (~1-1.5m thick) to confined coarse-grained gravel beds (~20-40cm thick). These types of fossil deposits occur within a more broadly horizontally distributed, fine-grained, and internally unstructured, alluvial overbank fines. Notably, only one site (QML1420 SWCC) records fossils from a depositional environment not typically flood plain. Most of the numerous, better structured, and well-sorted channel, point bar or crevasse splay deposits associated with the floodplain deposits are not fossiliferous. The fossil deposits occur in localised areas of the alluvial floodplain where they sit above the bedrock, with indications of some form of scouring prior to deposition, either directly scouring the bedrock or scouring into older floodplain sediments.

The fossil deposits are laterally discontinuous over scales of tens to hundreds of meters with limited opportunity directly correlate each site stratigraphically; however, within sites the relative positions of the fossils can be determined and thus relative stratigraphic height attained. SW9, SWJ and SWCC are locally confined deposits with lateral extents less than $50m^2$ (SWCC) to approximately $70m^2$ (SW9). SW3 is the only laterally extensive site, with fossil concentrations found in an area covering approximately $6000m^2$. Isolated remains have been recovered from a few additional sites, but these deposits are neither laterally extensive, nor fossiliferous. However, each site so far recorded has been sampled for dating and this will form part of a larger work.

QML1470 (SW9) (Supplementary Figure 11 & Supplementary Table 4)

The stratigraphy at QML1470 (SW9) is divided into three depositional units above Rewan Group bedrock. Unit A comprises cemented slightly gravelly sandy mud to muddy gravel (see Supplementary Figure 8 for sedimentological definitions) that is poorly sorted, poorly structured and containing angular medium gravel-sized clasts comprised only of Rewan Group. The unit is not fossiliferous and contacts the basement Rewan Group as an unconformity along its eastern margin. Unit B comprises slightly gravelly sandy mud to sandy mud, which is poorly sorted with limited sedimentary structure and includes sporadic lenses of angular fine to medium-sized gravel clasts of Rewan Group and reworked cemented sediment. The unit is not fossiliferous and contacts Unit A as a scoured surface forming an unconformity at the junction of both units, thickening to the west and southwest. Unit C is divided into three subunits (C1-C3). Unit C1 comprises a single bed of poorly sorted, poorly structured sediment ranging from mud to gravelly mud which forms a matrix supporting coarse pebble to small cobble-sized clasts. Gravel to pebble-sized clay-rich clasts that are likely rip-up clasts preserve some macro-floral remains, including macro-charocoal; however, there is limited sedimentological structure to define these structures. The lithified siliceous and bedrock clasts are dispersed throughout the bed, are concentrated toward its base, and are usually associated with large bones. These larger clasts include lithified angular to rounded pieces of petrified wood, silcrete, sandstone and siltstone that are derived from bedrock sources. Unique to all units within this sequence, unit C1 is fossiliferous preserving abundant vertebrate, invertebrate and plant remains throughout. Unit C1 contacts Unit B and the bedrock at an irregular scoured surface, which is identifiable as a slightly more gravelly muddy iron-oxide stained lens on which the lowermost fossils, pebbles and cobbles rest. This scoured surface forms a shallow northeast-southwest oriented basin into which unit C1 has accumulated. Fossils and the non-Rewan Group clasts are not found in unit B, or above the C1 bed in C2-C3. Unit C1 transitions into unit C2 with no definable contact between the two. The within-unit transition retains the massive slightly gravelly sandy mud matrix, but lacks the pebble to cobble clasts and fossils that define C1. Unit C3 is a slightly gravelly sandy mud lens with well-sorted sand that transitions from the poorly sorted, structureless unit C2. This transitional zone is not horizontal and thickens slightly to the southwest, but is not laterally extensive. The transition between units C2 and C3 is marked by a greater concentration of iron-oxide staining of the sediment. Unit C1 to C3 combined shows a general fining upwards trend with basal matrix-supported fossiliferous gravel to pebble and cobble bed that transitions into a well-sorted fine sandy mud with the absence of larger clasts and fossils. A shallow, clay-rich soil profile caps the site, and comprises fine carbonate nodules.

Fossil remains recovered from the site include flora and fauna representing both aquatic and terrestrial taxa. Floral preservation has only been observed at this site and is dominated by oxidised organic remains and impressions of leaves and seeds. Very small pieces/flecks of reworked charcoal of similar size to the clasts entraining them are sporadically recovered exhibiting significant weathering and are mostly oxidised. Leaves and seeds preserve limited structure; however, those leaves with thick sclerophyllous cuticle and rigid venation create good impressions when the matrix surrounding them is almost entirely fine clay. Similarly, seeds are found as both oxidised organic remains or moulds and casts. Oxidised leaves and seeds are found throughout unit C1 being

well preserved in clay-rich matrix but poorly preserved when included in more sandier or gravelly matrix. No plant remains have been recovered from units C2 or C3 overlying this. The distribution and orientation of plant fossil remains is haphazard, with leaves and seeds showing pre-burial fragmentation, splitting, curling and orthogonal breakage, indicating that the leaves were dry prior to deposition (Supplementary Figure 1 c, e & g). However, other plant remains show labile deformation within the sediment and entire portions preserved indicating soft plant tissue that had not completely dried out prior to deposition (Supplementary Figure 1 f, h–l). A small ~50cm x 50cm patch of dominantly clay matrix has produced the best preserved plant remains and has also returned low but present pollen counts (Supplementary Figure 32 and Supplementary Table 22). Tiny seeds, preserving very fine organs, are found preserved in relatively coarse sediment, indicating rapid burial with limited transport (Supplementary Figure 1 i–l). In particular, a seed from the Goodeniaceae (Supplementary Figure 1 i) family represents a member of a small-sized short-lived herbaceous taxon suggesting that this deposit was formed rapidly when seeds were available, perhaps within a season. These types of seeds do not survive intact and will germinate if sown; therefore, it would be expected that the time between seeding of this particular seed being deposited was very short, perhaps within weeks.

Aquatic and terrestrial invertebrate fossils are preserved in unit C1 as oxidised moulds and casts. Terrestrial insects are only preserved at QML1470 (SW9), represented by three-dimensionally preserved abdomens with oxidised elytra and suspected (but indeterminate) wing and limb impressions. The elytra compare closely to members of the weevil family, Curculionidae (Supplementary Figure 1 y–z), a diverse terrestrial insect group. Aquatic invertebrates include two freshwater bivalve taxa from the hyriidae and corbiculidae, both preserved as either moulds or casts with impressions of the commarginal ornament and dentition. These bivalves are small-sized (10mm-60mm shell length) and are found either as isolated or articulated valves not in life position, indicating that they have been displaced from their life habitat and transported pre-burial. No original shell material remains, nor has it been replaced. Oxidisation has occurred at the interface between where the internal or external organic bivalve has contacted the surrounding matrix. Freshwater crustaceans are represented by very rare oxidised gastroliths.

Sieving and sorting of approximately 500kgs of sediment from unit C1 recovered rare and poorly preserved vertebrate microfossils. Partial fish vertebrae, scales and broken spines are the most common remains, whilst the rarest fossils include a heavily rounded dasyurid molar, a fragment of scincid maxilla, few isolated murid teeth and a calcaneum. Of the sites preserving small vertebrates, QML1470 (SW9) is the least well preserved. In comparison, the larger vertebrate remains are the best preserved remains of all sites so far excavated and include both aquatic and terrestrial taxa, with the terrestrial fauna most abundant. Within the C1 bed, crocodilians are known from isolated teeth representing hatchling to large adult size. A single carapace piece is the only known freshwater chelid turtle fossil within the bed (Supplementary Figure 2, u). No large-sized teleost remains (e.g. vertebrae >5-10 mm in length) are present (Supplementary Figure 2, u-x).

Terrestrial taxa are predominantly represented by appendicular postcranial remains; however, Voorhies groups I-III including associated cranial, articulated and semi-articulated appendicular and associated axial remains of differing sizes are observed at similar proportions (Supplementary Figure 9). Breakage patterns and surface weathering features indicate a similar proportion of dry to wet fractures with little to no long-term surface exposure. Skeletal associations are observed for nine species (Supplementary Table 3) with clear articulation present in the pelvis of the only *Phascolonus gigas* individual (QMF57065) (Supplementary Figure 6) and semi-articulation of a hind limb of a small *Protemnodon* individual (QMF57165) (Supplementary Figure 5). Low minimum number of individual (MNI) counts for each taxon is supported by the element associations and supports rapid burial with limited time averaging of the deposit. Predatory modification of bone is observed and include puncture, gouge and cut marks made by crocodilians along with one macropodid rib preserving sequential cut and bone peel marks likely made by *Thylacoleo* (Supplementary Figure 10).

Bone long-axis orientations do not show a well-defined directional mode and bone orientations are not statistically significant (Supplementary Figure 11); however, there is a tendency for the longest bones (e.g. tibiae, femora and humeri) to be oriented in a northeast-southwest, or orthogonal to this, direction. This, albeit weak, tendency does reflect the overall shape and orientation of the shallow basin that has been infilled, indicating some influence of flow direction on the largest elements during deposition. This general direction of flow is reflected in the proximity of associated elements to one another, for example, the associated hind-limb of *Protemnodon* sp. (QMF57165) (Supplementary Figure 5, ag) has disassociated along a southwest to north east trajectory with the proximal pelvic element in the southwest with subsequently more distal elements dispersed in a general northeast direction from this point. This relationship is also seen in the associated *Phascolonus gigas* pelvis and hind limbs elements (QMF57065) (Supplementary Figure 6). Similar dislocation of elements, but in an orthogonal direction to this, is observed with the associated subadult *Diprotodon* skull, and isolated left and right dentaries (QMF54689), with each element distributed along a near east-west trajectory. These differing trajectories implicate a relatively turbid and changing flow regime during rapid deposition, possibly part of a localised hyperconcentrated flow.

Post-depositional modifications of bones and teeth within the deposit have been observed at different levels of frequency. In decreasing frequency modifications include: 1) cortical surface bone splitting due to clay contraction and swelling within matrix surrounding and internal to the bone (common); 2) medullary cavity collapse due to loss of internal integrity (collagen loss and lithostatic pressure) (common); 3) slight lateral and vertical dislocation of once competent bone elements due to a combination of clay sediment slippage (e.g. showing micro-slickensides) and opening of cracks due to long-term drying (rare); and 4) termite damage (very rare, a single observation). Some elements collected during the early period of excavation and preparation have experienced modifications due to human-induced accidental damage and preparation.

Supplementary Table 4. QML1470 (SW9) Sedimentological and Taphonomic Summary and Interpretation.

Site	Sedimentology	Sedimentary Structure	Fossil Preservation	Fossil Modification	Interpretation
**SW9**	Multimodal, mud-dominant matrix-supported poorly-sorted gravel to cobble bed transitions to well-sorted mud-dominated fine sand cap.<br><br>Iron-oxide precipitation.	Basal scour unconformity. Unstructured. Localised post depositional surface cracking.	Well preserved, articulated and associated terrestrial and aquatic invertebrate, vertebrate and plant taxa. Fine preservation of plants and insects. No coprolites.<br>Single definable fossiliferous bed 0.3-0.5m thick.	Crocodile, marsupial carnivore (thylacoleo) bone modification. Pre-depositional dry and wet bone fracturing. Post-depositional lithostatic compression and swelling clay bone-splitting and dislocation.<br>Dry leaf splitting preservation.	**Flood event on floodplain.** Short-lived high energy deposition (localised hyperconcentrated flow), rapid burial of proximal faunal and floral remains within a scoured floodplain. Transition into suspended load and fine sorting (~1-1.2m thick deposit), then vertical accretion.

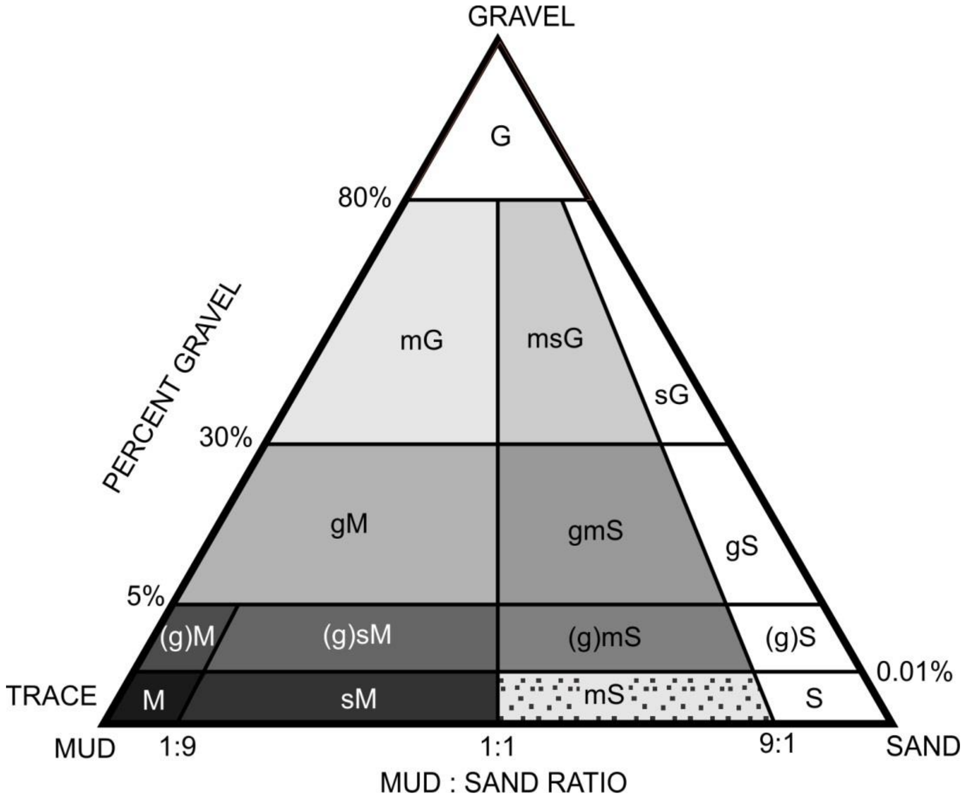

Supplementary Figure 8. Sediment classification and composition following Folk[60]. G – gravel; M – Mud; S – Sand; g – gravelly; m – muddy; s – sandy; (g) slightly gravelly.

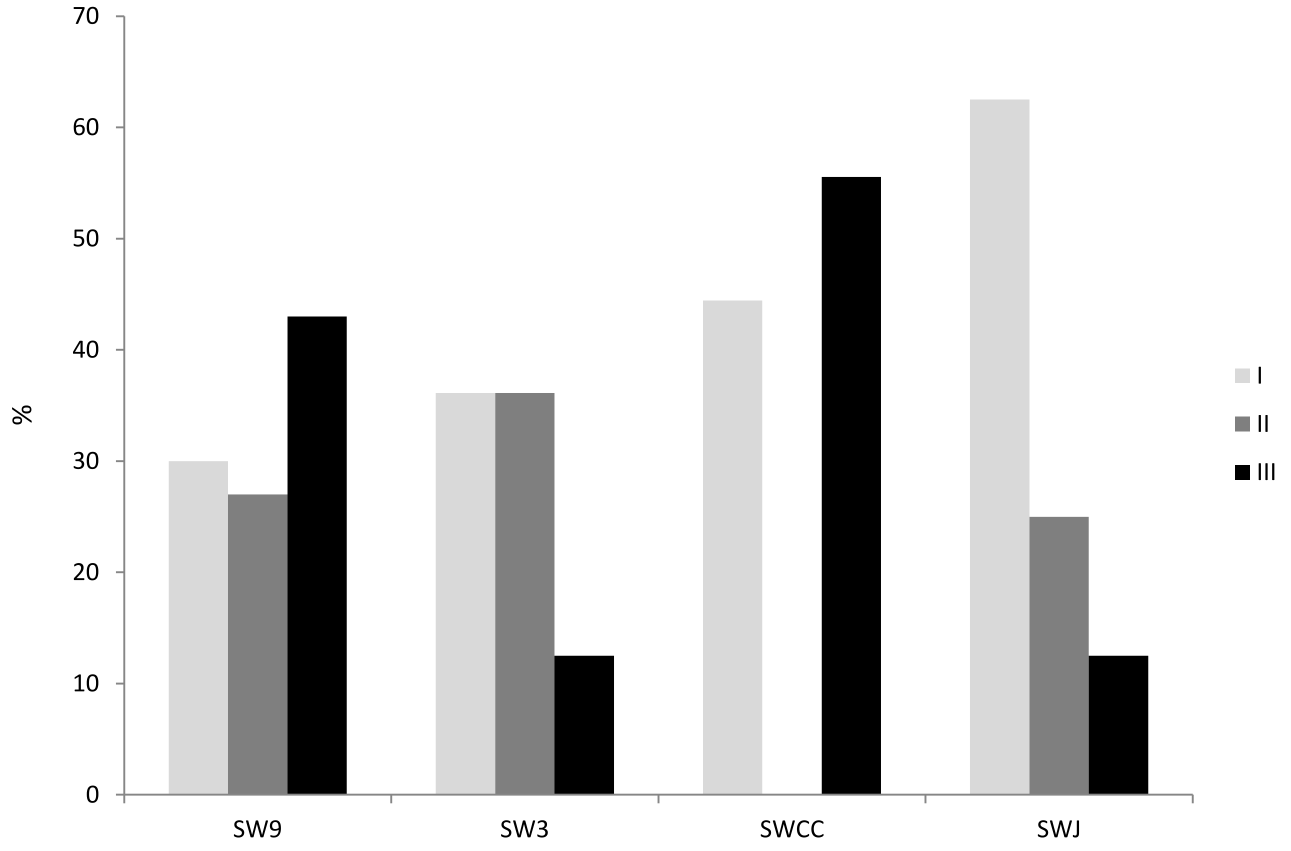

Supplementary Figure 9. Percentage of bone elements represented by Voorhies Group per site**.** Source data are provided as a Source Data file.

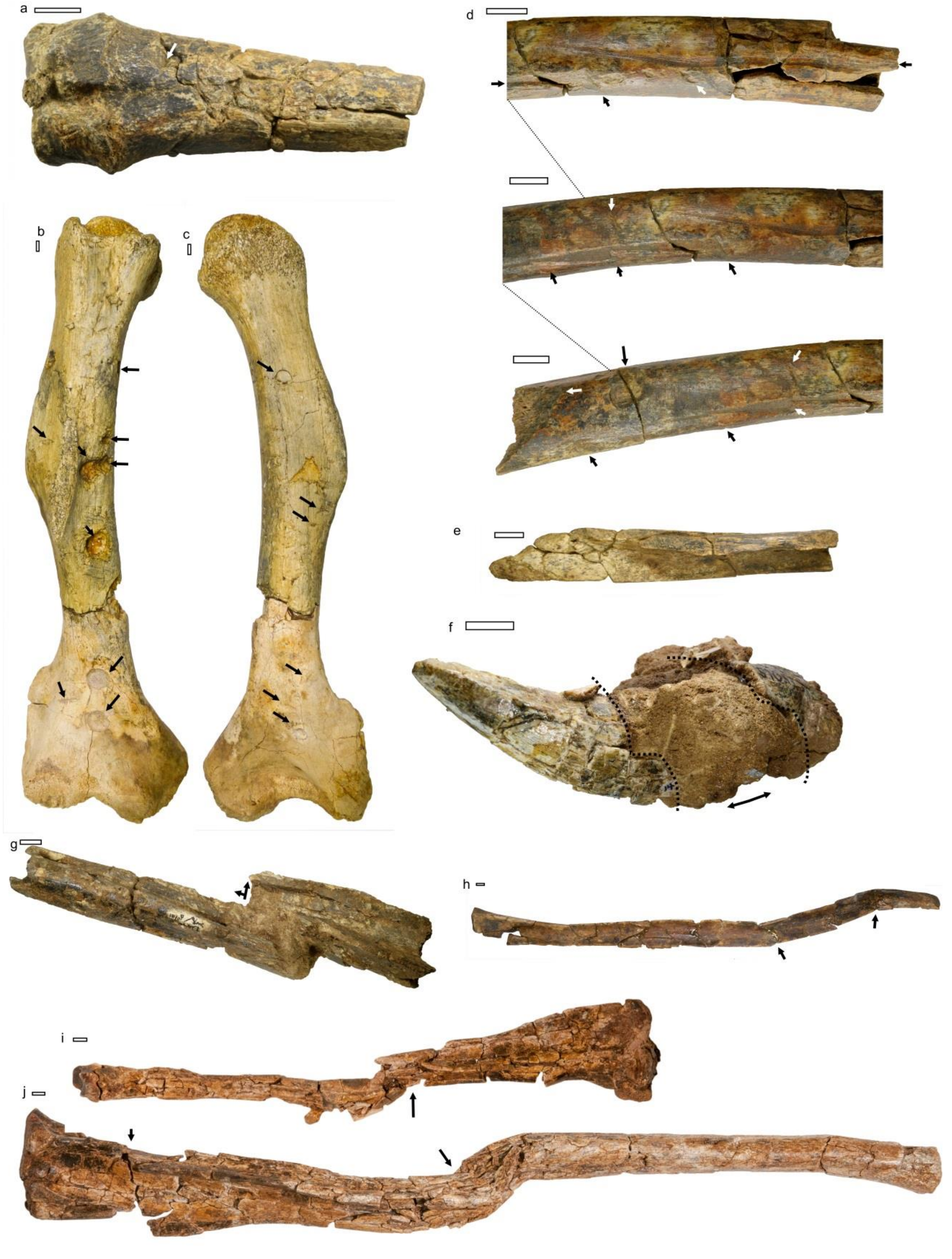

Supplementary Figure 10. Taphonomic examples. Puncture and cut marks (arrows) in (a-d); (a) distal macropodid fibula with crocodile puncture mark and dry fracture QMF59989; (b–c) right *Macropus* sp. humerus with crocodile puncture and cut marks, and unfused epiphyses QMF57036; (d) rib shaft showing consecutive cut and bone peeling along rib surface by *Thylacoleo* QMF59995. (e) piece of tibial shaft illustrating wet (left margin) and dry (right margin) fracture patterns QMF59988. (f-j) post-depositional subsurface deformation of fossils with direction indicated by arrows; (f+ g) movement through drying and crack filling with silt; (f) *Pallimnarchus* sp. tooth QMF57051; (g) macropodid tibial shaft QMF59952; (h–i) subsurface deformation of fossilised long bones; (h) *Macropus* sp. (giant) fibula QMF57039; (i) *Protemnodon* sp. tibia QMF57165; (j) *Macropus* sp. (giant) tibia QMF57039. Scale bars equal 10 mm (a, b, c, e, g, h, i, j) and 5 mm (d, f).

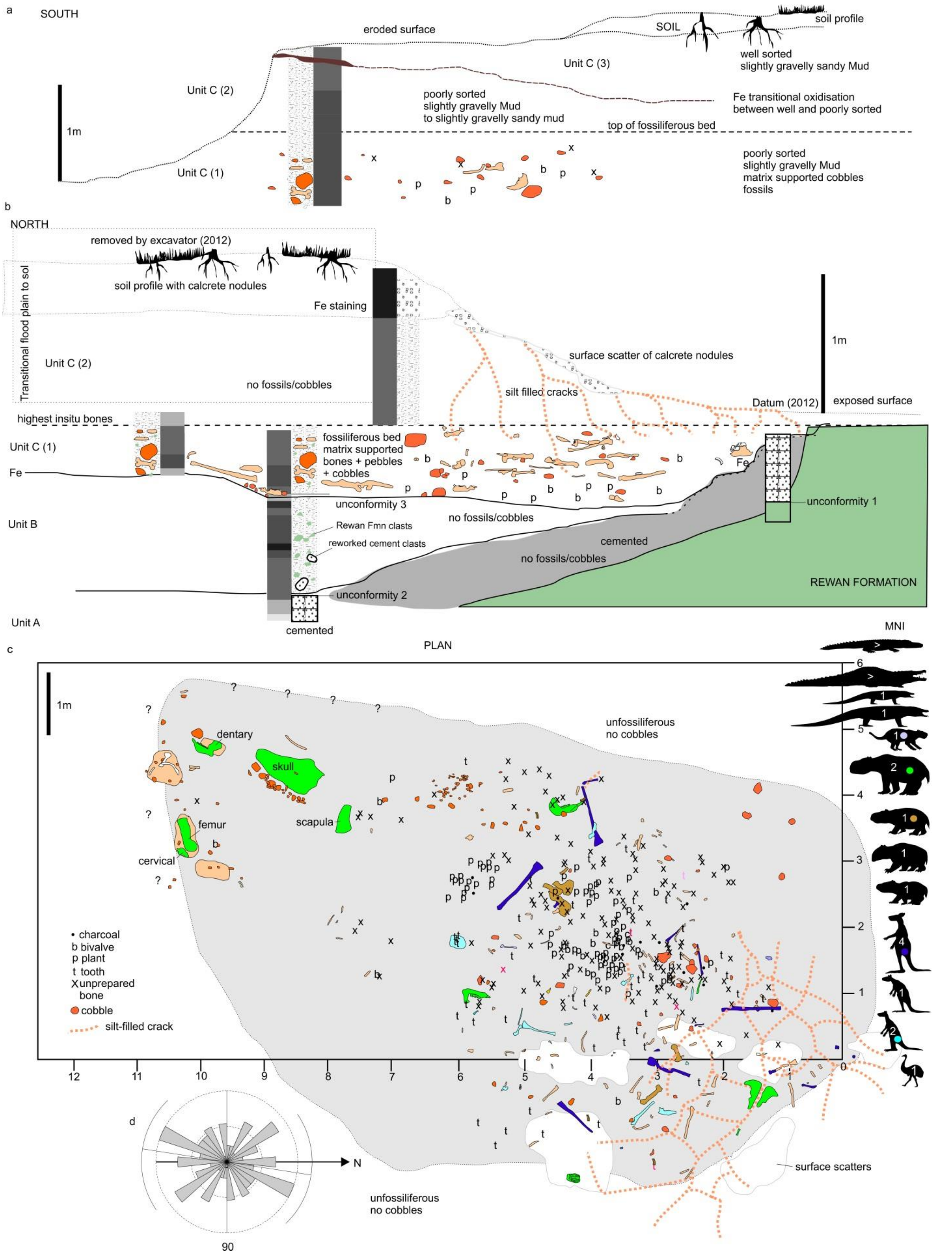

Supplementary Figure 11. Stratigraphic map of QML1470 (SW9) in South Section (a), North Section (b) and Plan view (c). In situ bone orientations indicated in rose diagram (d). Minimum number of individuals (MNI) of megafauna indicated within the silhouette. Sediment classification and composition key provided in Ternary diagram of Supplementary Figure 8.

QML1470 (SW3) (Supplementary Figure 12 & Supplementary Table 5)

The fossiliferous bed at QML1470 (SW3) is the most laterally extensive of the sites. Fossil vertebrate remains are found throughout the entire unit with the highest concentration at the north-northeastern margin of the exposed deposit. The unit contacts the basement bedrock as a sharp contact with and immediately transitioning into poorly sorted, poorly structured, slightly gravelly sandy mud. Occasional small lenses of angular to rounded Rewan Group gravels are present within the lower metre of the unit; however, these are localised and are not directly associated with bone accumulations. The unit is composed of relatively uniform sandy mud with the only definable component of change within the unit being the increasing concentrations of fossil bone up sequence and to the North. A soil profile is evident at the top of this unit.

Both aquatic and terrestrial vertebrate fauna have been found. No plant or terrestrial invertebrates are known and crustacean gastroliths are present, but rare. Terrestrial vertebrate faunal postcranial remains are the predominant vertebrate fossil recovered; however, aquatic vertebrate fauna are better represented at QML1470 (SW3) compared to QML1470 (SW9). Aquatic fauna are also represented by coprolites. Faunal remains predominantly represent large terrestrial taxa, only a single maxilla from a species of dasyurid has been found that represents small fauna. No fish remains have been recovered. Large portions of carapace and plastron represent chelid turtles, whilst isolated teeth, osteoderms, skull fragments, coprolites, postcranial remains and a partial dentary represent crocodilian remains. Appendicular elements are the most abundant component of terrestrial fauna; however, axial remains are better represented at SW3 than at other sites. Skeletal associations are recognised for three species found at the site (Supplementary Table 3).

Pre-depositional modification of bone is difficult to ascertain due to the heavy carbonate encrustation and surface corrosion of bone; however, bone modification through predator action includes puncture, gouge and cut marks on bones from crocodilians (Supplementary Figure 10, b & c). Pre-depositional fracture patterns are hard to discern due to the exposed weathering of this site prior to excavation. Excavated remains include almost complete elements suggesting that limited fracturing occurred prior to burial. Post-depositional modification includes surface cortical bone splitting through clay swelling; organic acid corrosion of bone surfaces and deposition of thick penetrating carbonate encrustation (caliche). This encrustation adheres very tightly to the bone and is difficult to remove to reveal original bone surface. However, when removal of the carbonate is possible, bone modifications are revealed, including evidence of wet bone fractures and predator modification.

Supplementary Table 5. QML1470 (SW3) Sedimentological and Taphonomic Summary and Interpretation**.**

Site	Sedimentology	Sedimentary Structure	Fossil Preservation	Fossil Modification	Interpretation
**SW3**	Mud-dominated, poorly-sorted matrix.<br><br>No cobbles or pebbles.<br><br>Carbonate precipitation.	Basal contact with bedrock.<br><br>Unstructured / massive.	Caliche-encrusted terrestrial and aquatic vertebrate fauna, invertebrates minor, no plants. Coprolites common. Concentration of fossil bones up profile ~2.5m thick.	Crocodile bone modification. Pre-depositional dry and wet bone fractures. Swelling clay surface bone splitting. Organic acid / root surface corrosion of surface bone.	**Vertical accretion on floodplain.** Low energy accretion on flood plain (muddier than SWJ). Dispersed fossil beds within a 2.5-3m thick depositional sequence.

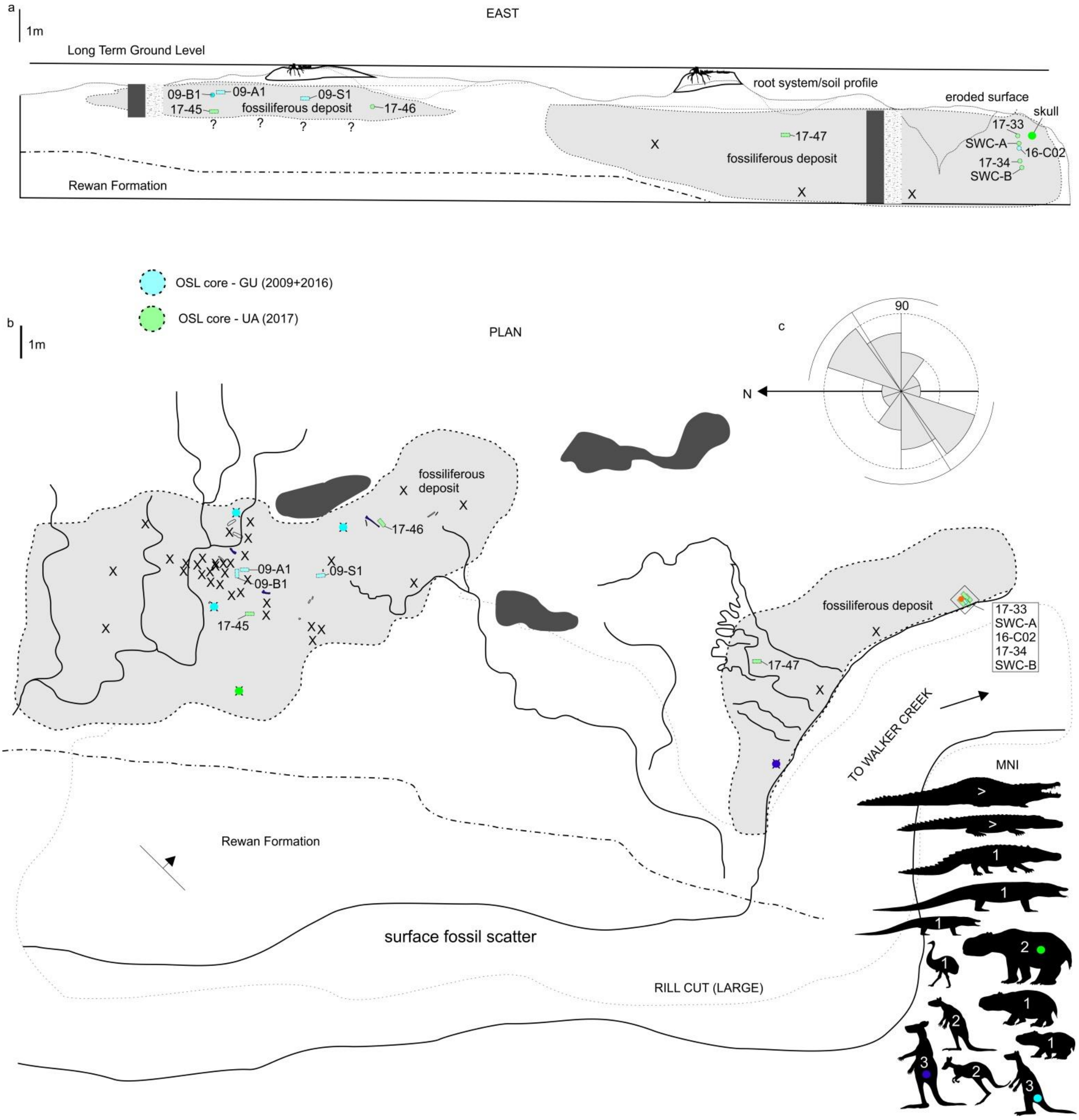

Supplementary Figure 12. Stratigraphic map of QML1470 (SW3) in East Section (a) and Plan view (b). *In situ* bone orientations indicated in rose diagram (c). MNI of megafauna indicated within the silhouette. Sediment classification and composition key provided in Ternary diagram of Supplementary Figure 8.

QML1470 (SWJ) (Supplementary Figure 13 & Supplementary Table 6)

The QML1470 (SWJ) fossils are exposed at the top of an eroded slope above a thick exposure of Rewan Group bedrock. Above this bedrock a series of exposed cemented beds form a basal unit (unit A) consisting of a coarsely to finely-graded sandy gravel with cemented calcrete rhizoliths. Contact between unit A and the unit above (unit B) is not observable, but the lowest observable point of unit B is un-cemented indicating a transition from cemented to un-cemented sediments between units A and B. Unit B is comprised of slightly gravelly muddy sand to muddy gravel, which is poorly sorted with limited structure. Unit C consists of two subunits C1 and C2; C1 is approximately 15cm thick and contacts the top of unit B which is a scoured surface unconformity. It consists of sandy gravel comprising angular coarse pebbles of Rewan Group. C1 transitions into the C2 bed which is the fossiliferous section and is comprised of well sorted gravelly muddy sand. A soil profile exists above unit C.

Only vertebrate fossils are known from SWJ. All vertebrate remains are rare and poorly preserved, including a ziphodont tooth, a piece of chelid carapace, mammalian vertebrae and appendicular elements. An exception to this overall poor preservation is a single, well-preserved, dorsal vertebra from *Varanus priscus*. With the exception of a small number of bones, no primary bone pre-depositional modifications are visible due to the extensive removal of most surface bone through post-depositional corrosive processes. Surface corrosion reveals internal trabeculae on most elements except the varanid vertebra. It is likely that bone in this deposit has undergone a significant degree of post-depositional alteration through the actions of organic acids, moreso than the corrosive features observed on bones from SW3.

Supplementary Table 6. QML1470 (SWJ) Sedimentological and Taphonomic Summary and Interpretation

Site	Sedimentology	Sedimentary Structure	Fossil Preservation	Fossil Modification	Interpretation
**SWJ**	Fine sand-dominated, well-sorted matrix.	Fining upwards from gravelly base, otherwise unstructured.	Very poorly preserved terrestrial and aquatic vertebrate taxa.	Surface bone corrosion, minor exceptions.	**Vertical accretion on floodplain**. Higher energy deposition compared to SW3 and SW9, however, lower energy deposition compared to SWCC. Dispersed fossil bed at the top of a 2.5-3m thick non-fossiliferous sequence.

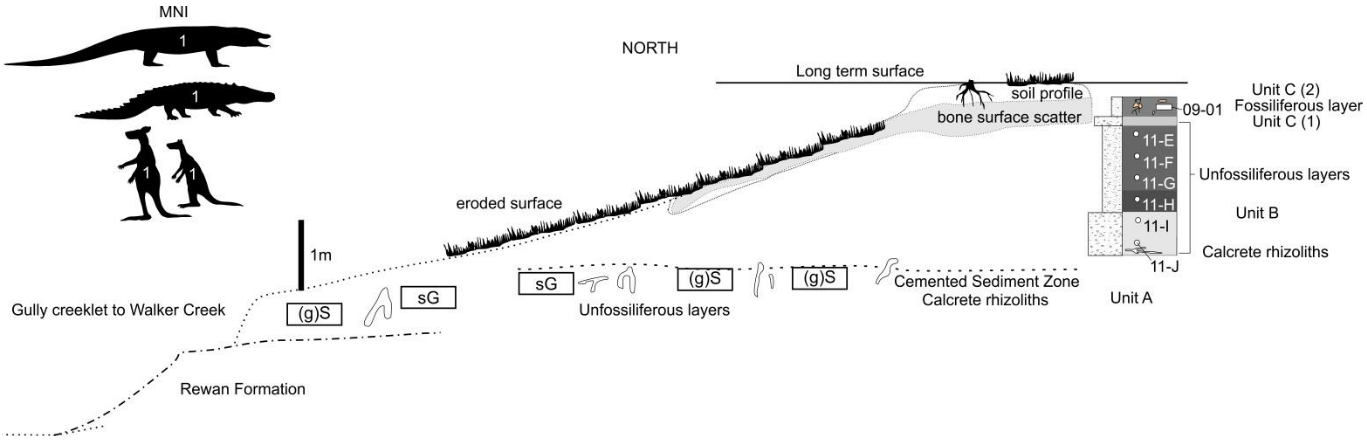

Supplementary Figure 13. Stratigraphic map of QML1470 (SWJ) in North Section (a). MNI of megafauna indicated within the silhouette. Sediment classification and composition key provided in Ternary diagram of Supplementary Figure 8.

SWCC (Supplementary Figure 14 & Supplementary Table 7)

QML1470 (SWCC) is the most sedimentologically complex deposit of the localities investigated, made up of five units A-E, with the basal unit contacting Rewan Group bedrock. Fossils have been recovered from units A-D, with the better preserved material derived from unit B. Unit A consists of a course-grained sandy to pebble-cobble matrix comprising angular to rounded Rewan Group gravel and small siliceous cobbles that fines to the northeast. Unit B contacts unit A at a sharp-based scour unconformity and consists of poorly structured gravelly muddy sand that fines upwards and toward the west, capped by an irregular mud drape. Unit C is a lens of coarse-grained sandy gravels to pebbles consisting of angular and rounded clasts that cut into unit B as a scour deposit and fines to the West. Unit D replicates unit B as a poorly structured gravelly muddy sand unit that fines upwards and to the East with a capping mud drape. It sharply contacts and bounds the top surface of both unit B and C laterally. Unit E is a poorly sorted, unstructured sandy mud unit that sharply contacts Unit D forming a scour unconformity. A soil profile is present above unit D.

Aquatic and terrestrial fauna are preserved within these deposits. Crustacean gastroliths and small corbiculid bivalve valves represent the aquatic invertebrate fauna. Aquatic vertebrates include small to large fish remains, crocodilian teeth and cranial remains and large numbers of turtle carapace and plastron pieces. Terrestrial vertebrate remains are rare, but include a diverse range of isolated mammalian and reptilian teeth, fragmented postcranial remains, and an isolated and well-preserved dorsal vertebra from *Varanus priscus*. The large vertebrate remains are fragmented into small pieces, close to the size of the largest entraining clasts within the matrix. The bone fragments show limited signs of rounding indicating limited transport time, but due to the coarseness of the matrix breakages are abundant. Smaller vertebrate remains are fragmented; however, they are generally better preserved also showing limited time of transportation. Dental association is recognised for one species (Supplementary Table 3).

Supplementary Table 7. QML1470 (SWCC) Sedimentological and Taphonomic Summary and Interpretation.

Site	Sedimentology	Sedimentary Structure	Fossil Preservation	Fossil Modification	Interpretation
**SWCC**	Variable grain-size between units, poorly sorted matrix.	Multi-directional sediment grading, with sharp basal scours and muddy drapes above unit.	Well-preserved but fragmented terrestrial and aquatic vertebrate taxa. Very well preserved micro fossils.	Variable – complete to fragmented, unmodified to rounded teeth and bones.	**Small inter / chute channel flash flood events.** Short-lived, high energy, multiple events proximal to a main channel (possibly a chute channel). Rapid burial with coarse sediment and post event mud drape.

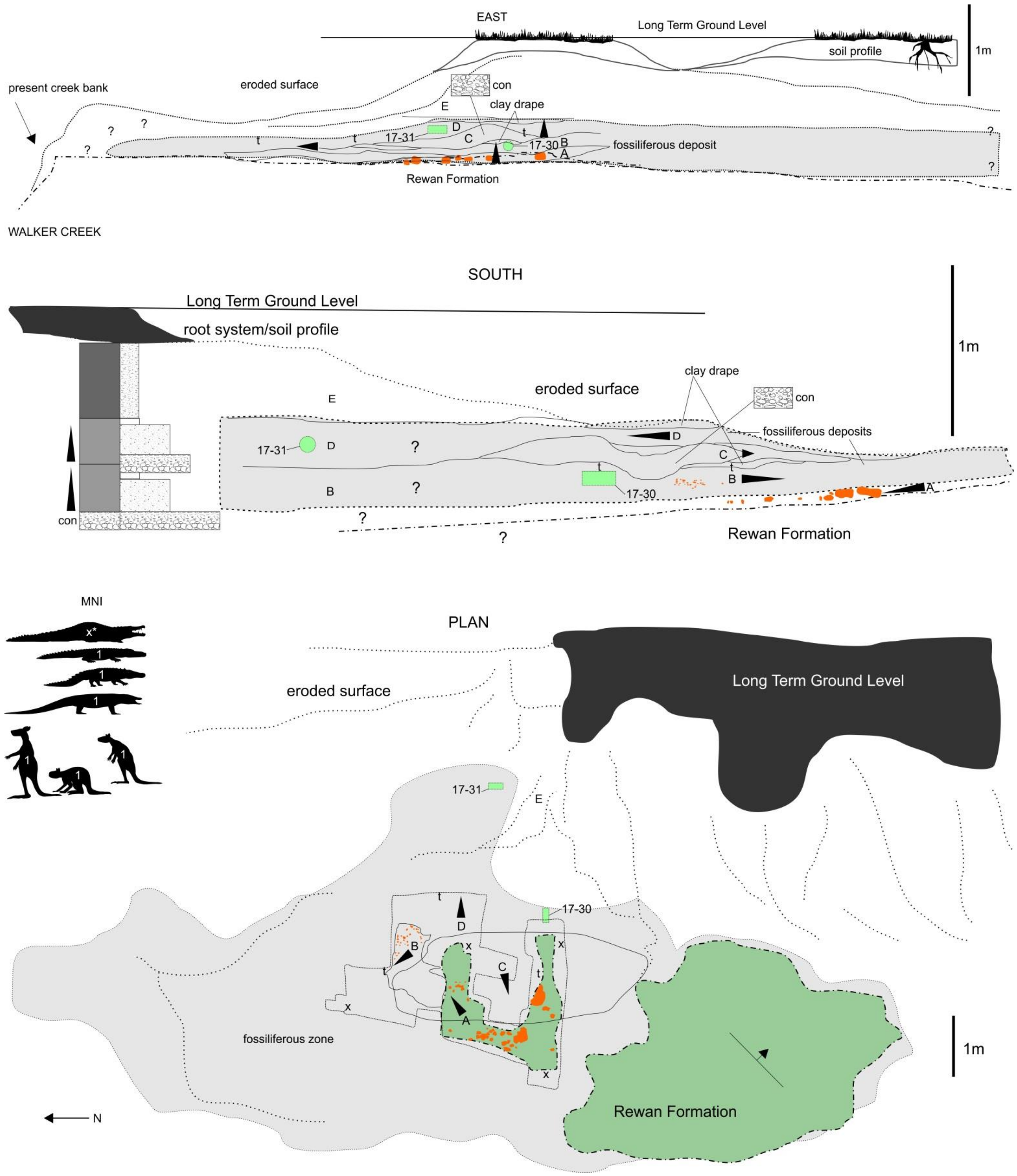

Supplementary Figure 14. Stratigraphic map of QML1470 (SWCC) in East Section (a), South Section (b) and Plan view (c). MNI of megafauna indicated within the silhouette. Sediment classification and composition key provided in Ternary diagram of Supplementary Figure 8. Arrows and letters indicate direction of grading and unit.

Supplementary Note 5: Geochronology of South Walker Creek (QML1470) sites.

Richard Lewis, Lee J. Arnold, Tim Pietsch, Scott A. Hocknull, Jon Olley, Rochelle A. Lawrence & Julien Louys.

Methodology – Field Collection

Dating samples were collected during field excavation and laboratory preparation from 2009 to 2017, with a focus on collecting adequate material to undertake multidisciplinary age determinations. Four independent dating techniques were used: optically stimulated luminescence (OSL); radiocarbon (^{14}C), uranium (U)-series, and electron spin resonance (ESR) dating. Supplementary Figures 12-15 show the locations of all dating samples collected from SW9, SWCC, SW3 and SWJ.

Site QML1470 (SW9)

QML1470 (SW9) has been most intensively sampled for dating. Twenty two OSL samples have been taken vertically and horizontally in an effort to capture as much of the depositional age of the target fossil bed of Unit C as possible. OSL cores were taken in vertical section within Unit C from C3 down to C1, along with two cores recovered in vertical sequence from the lower Unit B. One core sampled Unit C3 (OSL 11-01), five cores sampled Unit C2, twelve cores sampled Unit C1 (fossil bed), two cores sampled Unit B (SW9-16-C1 and SW9-16-C2) and two additional cores sampled deposits extralimital to the main site. Nine ^{14}C dating samples were derived from Unit C1. An additional six samples from Unit C1 were used for U-series and/ or ESR dating. Sample locations are provided in Supplementary Figure 15.

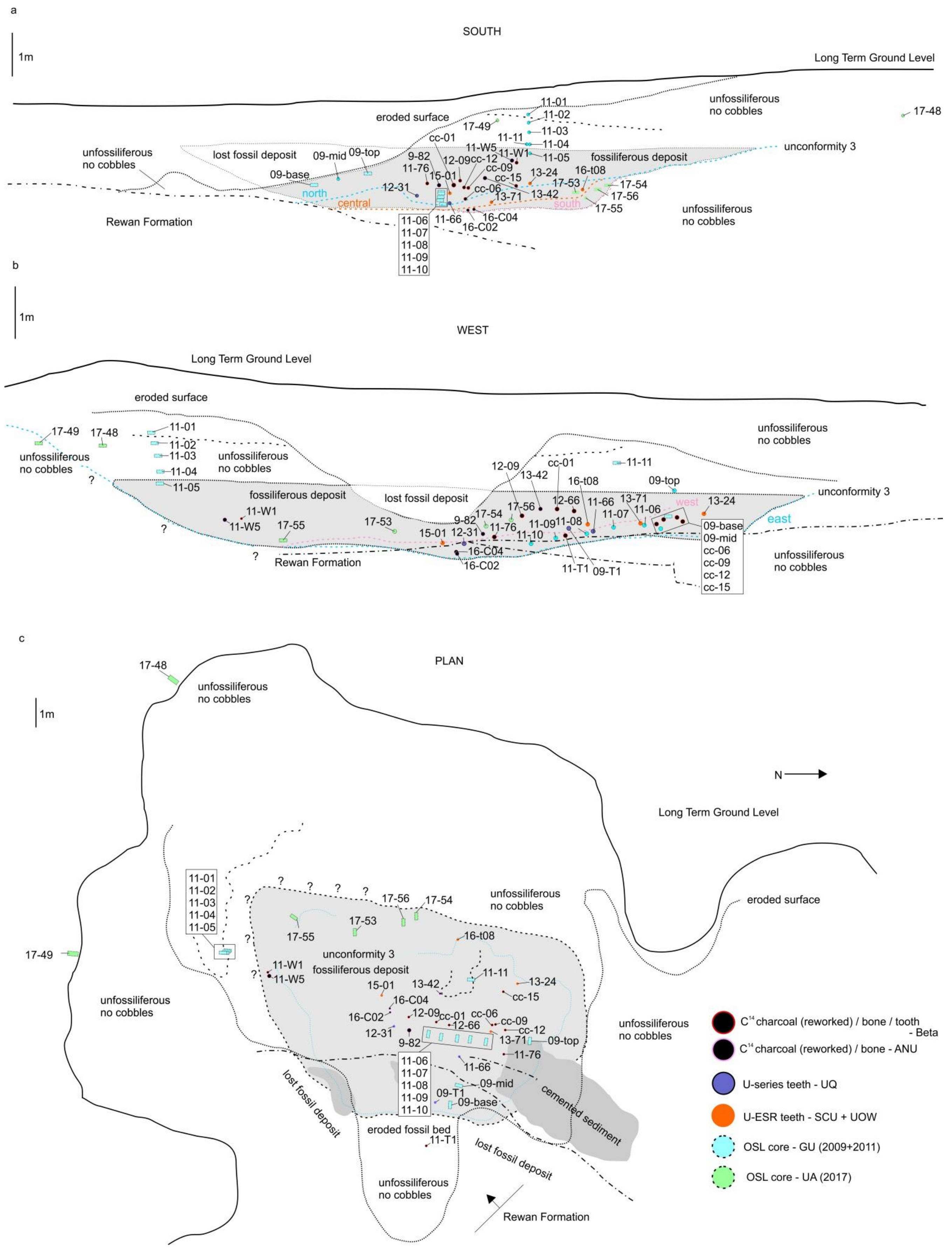

Supplementary Figure 15. QML1420 (SW9), dating sample locations within the SW9 site in southern (a), western (b) and plan (c) views.

Site QML1470 (SW3)

OSL and U-series / ESR samples were taken from this locality in 2009, 2016 and 2017. Only the OSL samples have been successfully processed. Eleven OSL cores were sampled vertically and horizontally across the entire site, focusing on two fossiliferous areas. The highest samples come from the northern section of the site (OSL-09 A1, and S1); however, these did not yield viable OSL ages (see below). The lowest sample (OSL-17-34) was collected toward the southern margin, at the base of a vertical series of samples taken at and below the level of a large *Diprotodon* skull. Sample locations are provided in Supplementary Figure 12.

Site QML1470 (SWJ)

OSL samples were collected from this locality in 2009 and 2011 along a vertical sequence crossing Units B and C (C1 and C2). Unit B and C1 are unfossiliferous, with the OSL sample from C2 representing the stratigraphically closest approximation of fossil depositional age. Sample locations are provided in Supplementary Figure 13.

QML1470 (SWCC)

Two OSL samples were collected in 2017 from Units B and D of this locality. Sample locations are provided in Supplementary Figure 14.

Optically Stimulated Luminescence

Richard Lewis, Lee J. Arnold, Tim Pietsch and Jon Olley.

Sample acquisition and preparation

OSL samples were collected from four sites within the SWC fossil deposit area QML1470 (SW9, SW3, SWJ, SWCC). The sampled sediments are considered to represent a combination of flood and channel deposits. The preserved sediments at SW3 and SWJ are thought to have been deposited quickly when channel banks were breached and flooding occurred. OSL samples at these sites have been taken in stratigraphic succession to ascertain whether there is evidence to support several generations of flooding events. Fossil-bearing units at SW9 and SWCC occur within sediments that are incised into underlying units and down to the bedrock as a result of fluvial activity. The fossil-bearing sedimentary layer at SW9 is unique in exhibiting vertical sand lenses that intrude into the fossiliferous sandy clays. These features are interpreted as a younger generation of sand infill deposited after desiccation and cracking of the clay sediment. Standalone sampling was therefore conducted on these sand layers to deduce whether they might yield complex burial dose characteristics and to evaluate whether they could bias the OSL age interpretations. For this purpose, a series of comparative samples were collected from the sand intrusion (SWC17-52), the clay layer (control; SWC17-56) and a combination of the two layers (SWC17-54).

OSL samples collected in 2011, 2015 and 2017 are considered to be the most informative and stratigraphically reliable for undertaking chronological evaluations of the four SWC sites. The following methodological descriptions and dating assessments therefore focus on these three groups of OSL samples. The preliminary group of OSL samples collected *ad hoc* by the excavation team in 2009 (Griffith University Batch 1) all showed clear evidence of contamination with younger material (e.g., Supplementary Figure 16). This contamination was attributed to sub-optimal sampling conditions and/ or sediment mixing via modern bovine trampling. These samples were subsequently not deemed suitable for OSL dating and a new study was undertaken in 2011 with a series of replacement samples collected from carefully excavated exposures at SW9 (SW9 1-11) and SWJ (SWJ E-J). An additional five OSL dating cores were collected from SW3 (SW3 A, SW3-15-C2 and SW3 B) and SW9 (SW9-16-C1 and SW9-16-C2 – both from Unit B) in 2015 and 2016 respectively. However, these samples yielded very low grain recovery ratios (i.e. number of grains accepted as a proportion of grains analysed). The low accepted grain yields (7-11 single-grain D_e values per sample), which may be partly related to variations in sediment sourcing (and hence luminescence properties) between different units, are considered insufficient to characterise the true D_e scatter affecting these five samples and to derive meaningful burial dose estimates, hence they have not been considered further in our site chronological evaluations.

Finally, in 2017 additional cores were recovered from SW9, SW3 and SWCC that included in situ dosimetry measurements to provide corroboration of previous samples and to broaden the range of sites assessed.

The 2017 (SWC17) and 2015 (SWC) OSL samples were extracted from their coring tubes and prepared under controlled lighting conditions (subdued red lighting) at the Prescott Environmental Luminescence Laboratory (The University of Adelaide; AU). The 2011 OSL samples (SW9-(X)) were extracted and prepared under similar safelight

conditions at the Environmental Forensics Laboratory (Griffith University; GU). Purified coarse-grain quartz fractions (SWC17 & SWC = 212-250 µm; SW9-X = 180-212 µm) were prepared using standard procedures [61]. This process included wet sieving of sediments, chemical treatment with HCl and H_2O_2 to remove carbonates, organic material and clay, and isolation of quartz fractions through heavy liquid density separation. Isolated quartz grains were etched with concentrated HF (48%) for 40 mins (a double etch was used for the GU 2011 samples) to remove the alpha-irradiated outer layer of the quartz grains, and subsequently washed with HCl to dissolve any fluoride precipitates. The etched quartz grains were re-sieved using a smaller mesh size to ensure any remaining partially etched feldspars and smaller disaggregated quartz grains were removed prior to OSL measurement.

Instrumentation and Equivalent dose (D_e) measurement

The 2011 (GU), 2015 (AU) and 2017 (AU) samples were measured using Risø TL-DA-20 readers equipped with 10 mW Nd:YV04 (532 nm) single-grain laser attachments, EMI 9235QA photomultiplier tubes fitted with 7.5 mm thick Hoya U-340 filters, and calibrated $^{90}Sr/^{90}Y$ β sources. D_e measurements were made using standard single-grain aluminium discs drilled with an array of 300 µm x 300 µm holes, the positions of which had been individually calibrated to account for spatial variations in the beta dose rate across the disc plain. Single-grain OSL D_e measurements were conducted using single-aliquot regenerative-dose (SAR) protocols [62] that had been modified to allow measurement of individual grains[63] (Supplementary Table 8). D_e measurements were calculated by interpolation of the sensitivity-corrected natural signal onto a single saturating exponential fit of the sensitivity-corrected dose response curve (Supplementary Figure 17). For the 2015 and 2017 samples (AU), the sensitivity-corrected dose response cures were generated by subtracting the background signal (last 0.25 s of stimulation) from the integrated signal measured over first 0.10 s – 0.18 s of laser stimulation (adjusted to avoid integrating the non-decaying part of the signal, and to obtain dose-response curve fits with the smallest possible curve-fitting errors). The dose response curves of the 2011 (GU) samples were determined from the initial 0.1 s of each OSL decay curve, using the final 0.2 s to estimate the background count rate.

OSL dose recovery tests were undertaken on representative samples from various locations (SWC17-53/SW9, SWC17-B/SW3, SWC17-G/SWJ) to assess the suitability of the SAR preheating conditions used in this study. Multi-grain OSL dose recovery tests were first performed on sample SWC17-B to determine optimum preheating conditions (using blue LED OSL stimulation instead of green laser stimulation in steps 4 and 7 of the AU SAR sequence shown in Supplementary Table 8). A known laboratory dose of 100 Gy was applied to groups of aliquots after optically bleaching their natural OSL signals using two 1,000 s blue LED stimulations separated by a 10,000 s pause (to ensure complete decay of any phototransferred charge in the 110 °C TL trap). Regenerative dose preheat treatments (PH1) of either 220°C for 10 or 240°C for 10 s, and a test dose preheat treatment of 160°C for 10 s, were found to yield reliable weighted mean measured-to-given dose ratios and recycling ratios at 2σ (Supplementary Figure 18). The suitability of one of these preheat combinations (PH1 = 240°C for 10 s; PH2 = 160°C for 10 s) was examined in greater detail at the single-grain scale of analysis. Single-grain OSL dose recovery

tests were made on three samples following bleaching of their natural signals (using the same conditions as with the multi-grain dose recovery tests) and administering a known dose of 100 Gy. Weighted mean measured-to-given dose ratios for this suite of samples ranged from 0.95±0.05 to 0.96±0.02, and overdispersion values varied between 2-11% (Supplementary Figure 19, Supplementary Table 9). Notably, these dose recovery ratios are within 2σ of unity, and therefore support the general suitability of the single-grain D_e measurement conditions used in this study.

For the 2015 and 2017 AU samples, single-grain OSL D_e estimates were not considered suitable for final age calculation if they exhibited any of the following properties (Arnold, Duval [64,] Demuro, Arnold [65]): (i) their net T_n signals were <3σ above the late-light background; (ii) recycling ratios (sensitivity-corrected luminescence responses (L_x/T_x) for two identical regenerative doses) were not consistent with unity at 2σ. For the single-grain OSL measurements, the recycling ratio test was performed using both a low-dose and high-dose regenerative dose cycle; (iii) the OSL-IR depletion ratio [66] was less than unity at 2σ; (iv) the recuperation ratio, calculated as the ratio of the sensitivity-corrected 0 Gy dose point (L_0/T_x) to the sensitivity-corrected natural (L_n/T_n), was >5%; (v) the L_n/T_n value intercepted the saturated part of the dose-response curve (L_n/T_n values were equal to $2D_0$ saturation limit of the dose-response curve at 2σ); (vi) the dose-response curve displayed anomalous properties (i.e., zero or negative response with increasing dose) or very scattered L_x/T_x values that could not be successfully fitted with the Monte Carlo procedure; (vii) the sensitivity-corrected natural signal (L_n/T_n) did not intercept the sensitivity-corrected dose-response curve; (viii) the net D_e uncertainty is >50%. The single-grain OSL rejection statistics for the 2015 and 2017 AU samples are shown in Supplementary Table 10.

The single-grain quality assurance criteria used for the 2009 GU samples are based on those of Pietsch [67] and Pietsch, Nanson [68]. Single-grain OSL D_e estimates were not considered suitable for final age calculation if they: (i) failed to produce a measurable (i.e. >3 standard deviations above background) OSL signal in response to the natural test dose; (ii) had OSL decay curves that did not reach background after 1 s of laser stimulation; (iii) produced natural OSL signals that did not intercept the regenerated dose-response curves; (iv) had unacceptable sensitivity changes throughout the measurement cycle, i.e. they were rejected if either of the second or third test dose signals varied in sensitivity from the natural test dose by more than 20%; (v) had dose response curves with observable recuperation, as indicated by 0 Gy dose points with a sensitivity-corrected OSL response beyond zero at 1σ, and (vi) exhibited recycling ratios that were not consistent with unity at 1σ.

The accepted single-grain D_e distributions for the 2011, 2015 and 2017 OSL samples are presented as radial plots in Supplementary Figures 20-25. Supplementary Table 11 provides a summary of the D_e statistics and age model preferences for the various samples from SW9, SW3, SWJ and SWC-CC. The single-grain D_e distributions are generally characterised by moderate D_e scatter and overdispersion values ranging between 25-35%, though there are some noteworthy exceptions. Samples SWC17-53 and SW9-2 exhibit the lowest overdispersion values (22 ± 3% and 22 ± 4%, respectively) and the most homogenous D_e distribution of the thirty two AU and GU samples (Supplementary Figures 23 & 25). These D_e characteristics are consistent with those typically reported for ideal

(well-bleached and unmixed) single-grain D_e datasets at 2σ (e.g. the global average overdispersion of 20 ± 1%; Arnold and Roberts [69]). These two samples have therefore been used to derive a site-specific, baseline assessment of the overdispersion expected for well-bleached and unmixed sediments in the SWC basin.

Twenty eight of the remaining thirty OSL samples exhibit moderate D_e scatter and slightly higher overdispersion values of 26 ± 4% to 40 ± 6%, (Supplementary Table 11). However, the overdispersion values of these twenty eight samples are all consistent at 2σ with the overdispersion value of our site-specific 'best-case scenario' samples (SWC17-53 and SW9-2), suggesting limited influences of additional extrinsic D_e scatter. Most of these D_e datasets are characterised by largely unstructured and broadly symmetric D_e scatter, with a limited number of outlying D_e values found either side of the weighted mean shaded bands on the radial plots (e.g., SWC-E, SWC-B, SWC17-33). Some of these samples show more asymmetric D_e scatter, including possible leading-edges of low D_e values or elongated tails of higher D_e values (e.g., SWC17-55, SW9-9, SW9-3), which might be indicative of heterogeneously bleached single-grain populations (Arnold, Bailey [70,] Arnold, Roberts [71]). However, the limited number of accepted D_e values obtained for some of these samples (i.e., *n* <50) makes it difficult to fully resolve their underlying D_e characteristics.

The remaining two samples from the combined SWC dataset (SWC17-49 and SW9-11) exhibit complex D_e distributions characterised by very high overdispersion values (97–116%) and multiple discrete dose populations (see details of finite mixture model (FMM) fitting below) (Supplementary Figures 23 & 25). These multimodal D_e datasets are consistent with those commonly reported for samples that have been affected by locally intruded, young grain populations e.g., [69, 72] or certain sources of beta dose heterogeneity (Nathan, Thomas [73,] Mayya, Morthekai [74,] Arnold, Demuro [75]).

We have considered a range of statistical age models to characterise each single-grain D_e distribution and to derive representative burial dose estimates for the AU and GU OSL samples, namely: the central age model (CAM), which would be considered suitable for well-bleached and undisturbed sediments (e.g., Galbraith, Roberts [76]), the three-parameter minimum age model (MAM-3) and the four-parameter minimum age model (MAM-4), which are generally considered useful for isolating the most suitably bleached dose components in otherwise heterogeneously bleached samples (Arnold, Bailey [70,] Arnold, Roberts [71,] Bailey and Arnold [77]), and the FMM, which can be used to identify discrete dose components in samples affected by post-depositional mixing (Arnold and Roberts [69,] Arnold, Demuro [72,] Galbraith and Green [78]). The choice of age model for final burial dose estimation has been made on a sample-by-sample basis according to the maximum log likelihood (llik or L_{max}) criterion of Arnold, Roberts [79] (i.e., using the age model that provides the most statistically suitable fit for each empirical dataset after taking into consideration model parameterisation complexity), in conjunction with consideration of the depositional contexts of the SWC samples.

Full details of the llik criterion and age model fitting results are provided in Supplementary Table 12. According to the llik criterion, the CAM is deemed the most statistically suitable age model for burial dose estimation for twenty nine of the thirty two GU and AU OSL samples. The MAM-3 is marginally favoured over the CAM for one of

the three remaining samples (SWC H) according to the llik criterion (Supplementary Table 12). However, the llik scores for this sample seem to be unduly influenced by the presence of a single, precise high D_e value (Supplementary Table 13), as indicated by sensitivity tests performed on the same D_e dataset after elimination of this outlying grain (Supplementary Table 12). Given the limited number of individual D_e values obtained for sample SWC H (n = 39) and the disproportionate influence of this single outlying grain on the llik scores, we have opted to derive the final burial dose using the CAM to ensure consistency with the other OSL samples from site SWJ.

Samples SWC17-49 and SW9-11, which display complex D_e distributions and overdispersion values of 97-116% (Supplementary Figures 24 & 25), are not adequately represented by the CAM according to the llik criterion (Supplementary Table 12). Given the multimodal D_e scatter apparent for these two samples, we have applied the FMM to formally identify any discrete dose components that may be present. As detailed in Supplementary Tables 13-14, the optimum FMM fits identify three discrete dose components for each sample. Assuming that the multiple discrete dose components of samples SWC17-49 and SW9-11 can be explained by localised post-depositional mixing or beta dose rate heterogeneity (which, along with possible contamination during sampling, seem the most feasible explanations in this sedimentary context), it follows that the bulk (sample-average) dose rate of these two samples may not be entirely representative of that experienced by the dominant dose components during burial. Owing to the impracticalities of retrospectively deriving a component-specific dose rate for the identified FMM components, these two samples are not deemed suitable for dating and have not been considered further in out chronological evaluations of SW9.

Dose rate evaluation and age calculation

Environmental dose rates (Supplementary Table 11) have been calculated using a combination of *in situ* field gamma spectrometry (FGS) and low-level beta counting (2017 AU samples), and high resolution gamma spectrometry (HRGS) (2015 AU samples and 2011 GU samples) (Supplementary Table 11, 15 & 16). Gamma dose rates were determined from *in situ* gamma spectrometry measurements made with a 3 inch Canberra NaI:Tl detector, following the 'energy windows' method [80]. External beta dose rates for the 2017 AU samples were determined from measurements made using a Risø GM-25-5 beta counter [81] on dried and homogenised, bulk sediments collected directly from the OSL sampling positions. Background-subtracted count rates were measured for three aliquots of each sample and compared with net count rates obtained simultaneously for a loess sediment standard with known U, Th and K concentrations [82]. HRGS measurements were performed at the University of Adelaide and the Forensic and Scientific Services department of Queensland Health, and used to derive the external gamma and beta dose rates of the 2015 AU and 2011 GU samples, respectively. Additional HRGS measurements were also conducted on the 2017 AU samples to evaluate the state of secular equilibrium in their ^{238}U, ^{235}U and ^{232}Th decay series. To calculate representative dose rates from the HRGS radionuclide concentrations, we have considered the proportional beta and gamma dose rate contributions from different

parent or daughter isotopes measured in the ^{238}U and ^{232}Th decay series, and we have assumed that the present-day state of (dis)equilibrium observed in the ^{238}U and ^{232}Th decay chains has prevailed throughout the burial period. Cosmic-ray dose rates have been calculated using the approach described in Prescott and Hutton [83] after taking into consideration site altitude, geomagnetic latitude, density, thickness and geometry of sediment overburden. A small, assumed internal (alpha plus beta) dose rate of 0.03 ± 0.01 Gy / ka has been included in the final dose rate calculations, based on published ^{238}U and ^{232}Th measurements for etched quartz grains from a range of locations [84, 85, 86, 87] and an alpha efficiency factor (*a*-value) of 0.04 ± 0.01 [88, 89].

Radionuclide concentrations and specific activities have been converted to dose rates using the conversion factors given in Guérin, Mercier [90] and Stokes, Ingram [91], making adjustments for beta dose attenuation and long-term sediment moisture contents [92, 93, 94]. The present-day sediment water contents of the SWC samples are not considered to be entirely representative of those prevailing throughout the sample burial periods because (i) the excavation pits and sediment exposures had partially dried out prior to sampling, (ii) OSL samples were collected during dry-season months, and (iii) local hydrological conditions are known to have been significantly wetter at various times in the past (see Supplementary Note 4 for stratigraphic and sedimentological descriptions). To determine more suitable long-term sediment moisture contents, we examined the range of present-day 'proportional saturated water content' values (i.e., present-day water contents / saturated water contents x 100) obtained for the 2015 and 2017 OSL samples. The highest proportional saturated water content for the SWC samples (31% of saturated water content; obtained from a freshly dug sampling exposure at SW3) was used to establish a more reliable estimate of present-day water content (as a proportion of sediment saturation capacity) in the absence of any prior sediment exposure bias. Based on these results, and factoring in the potential for intermittently wetter climatic conditions and significant flooding of SWC basin in the past (based on the preserved sedimentology and faunal remains), we have adopted conservative long-term sediment moisture contents of 40% (instead of 31%) present-day saturated water contents for each OSL sample. The long-term sediment moisture contents for the 2015 and 2017 samples range between 10 – 38% of dry sediment weight (Supplementary Table 11, 15), and have been assigned a 1σ relative uncertainty of 20% (40% at 2σ) to accommodate any variations in hydrologic conditions during burial. Whilst 40% of the saturated water content is our best estimate of long-term water content, the effect of assuming a value closer to the measured present-day water content would be modest. For example, the incorporation of 30% saturated water content for SW9 samples would produce a weighted average burial age of 40.5 ± 1.6 ka (within error of the weighted mean age reported in Supplementary Table 17 which uses 40%). As saturated water content data was not originally recorded for the 2011 GU samples, we have used the average long-term water content calculated for the 2015 and 2017 AU samples (22 ± 4% of dry sediment weight) to derive surrogate long-term estimates for dose rate determination (Supplementary Table 11, 15).

The HRGS radionuclide activities obtained for each OSL sample are summarised in Supplementary Table 16. The daughter-parent isotopic ratios for ^{238}U, ^{226}Ra, ^{210}Pb, ^{228}Ra and ^{228}Th are consistent with unity at either 1σ or 2σ for all samples, confirming that the ^{238}U and ^{232}Th chains are in present-day secular equilibrium. There is evidence of

minor disequilibrium in the ^{238}U series for five samples (albeit not statistically significant at 2σ), with samples SW9-2 and SW9-11 exhibiting ^{226}Ra:^{238}U activity ratios of 0.6, and samples SW9-5, SW9-6 and SW9-7 exhibiting ^{226}Ra:^{238}U activity ratios of 0.7 (Supplementary Table 16). These results imply the uptake of unsupported ^{238}U following burial or the loss of radon (^{222}Rn) gas (since we have used the post-radon daughter emissions of ^{214}Pb and ^{214}Bi to derive ^{226}Ra activities). The minor secular disequilibrium observed for these samples is not sufficient to change our calculated ages beyond those that would result from assuming equilibrium conditions have persisted throughout the burial period. Dosimetry modelling studies undertaken elsewhere have demonstrated that isotopic disequilibria of similar magnitudes are only likely to give rise to minor deviations (<5%) in long-term dose rate estimates[95, 96, 97]. Such systematic biases would be significantly less than the existing uncertainty ranges on our final dose rate estimates.

Supplementary Table 11 summarises the final ages for the 2011, 2015 and 2017 OSL samples from sites SW9, SWC-CC, SW3 and SWJ. Tables 17-18 summarise the weighted mean OSL ages calculated for the main fossil unit of each site. To derive these weighted mean estimates, individual OSL ages have been combined from the fossiliferous and non-fossiliferous sub-layers of the main fossil unit(s) at each site (e.g. Unit C1-C3 at SW9, Units B and D at SWC-CC), since these sub-units are not separated by any major unconformities and likely represent related phases of a broader depositional sequence. Samples from underlying non-fossiliferous unit that are separated from the main fossil unit(s) by an erosional unconformity (e.g. Unit B at SWJ) are not included in the weighted mean OSL age calculations shown in Table 17. The weighted mean GU and AU OSL ages obtained for the main fossil unit (Unit C) at SW9 are statistically indistinguishable from each other, confirming the validity of our inter-laboratory comparison results. The comparative OSL samples collected from the sand intrusion and clay layers at SW9 also yielded consistent ages (e.g., SWC17-52, SWC17-54, SWC17-56; Supplementary Table 11), suggesting that they represent broadly coeval deposits (relative to the size of our OSL dating uncertainties). The fossil deposits preserved at the SWC sites exhibit weighted mean OSL ages ranging from 41.3 ± 1.9 ka (SW9) to 58.2 ± 6.1 ka (SWJ) (Supplementary Table 17). All of the final OSL ages for SWJ have been derived from Unit B, which underlies the main fossil unit (Unit C). As such, the weighted mean OSL age for this site (65.6 ± 2.2 ka) represents a maximum age estimate for the formation of the overlying megafaunal deposit.

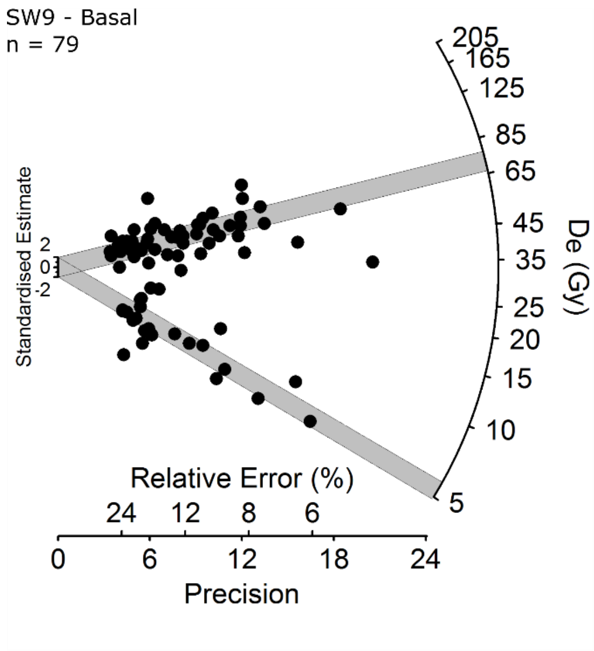

Supplementary Figure 16. Example of a contaminated single-grain D_e distribution from the preliminary group of OSL samples collected in 2009 (sample SW9-Basal). The grey shaded bands correspond to discrete dose component identified by the optimum finite mixture model (FMM) fit (Supplementary Tables 13-14 for FMM fitting details).

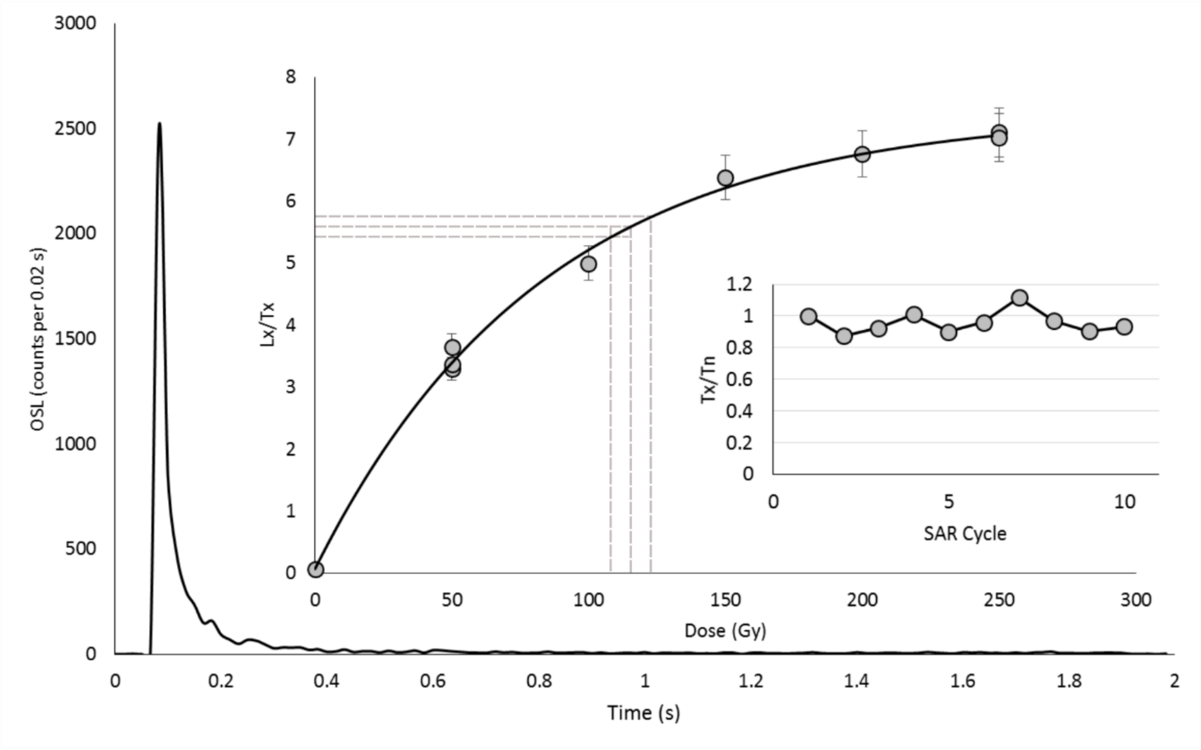

Supplementary Figure 17. Examples of a single-grain OSL decay and dose response. The main plot shows the natural OSL signal decay curve for a grain from sample SWC17-31. The secondary and tertiary inset graphs (left to right) show the dose-response curve for this grain and its normalised test dose sensitivity (Tx/Tn) response through the SAR measurement cycles, respectively.

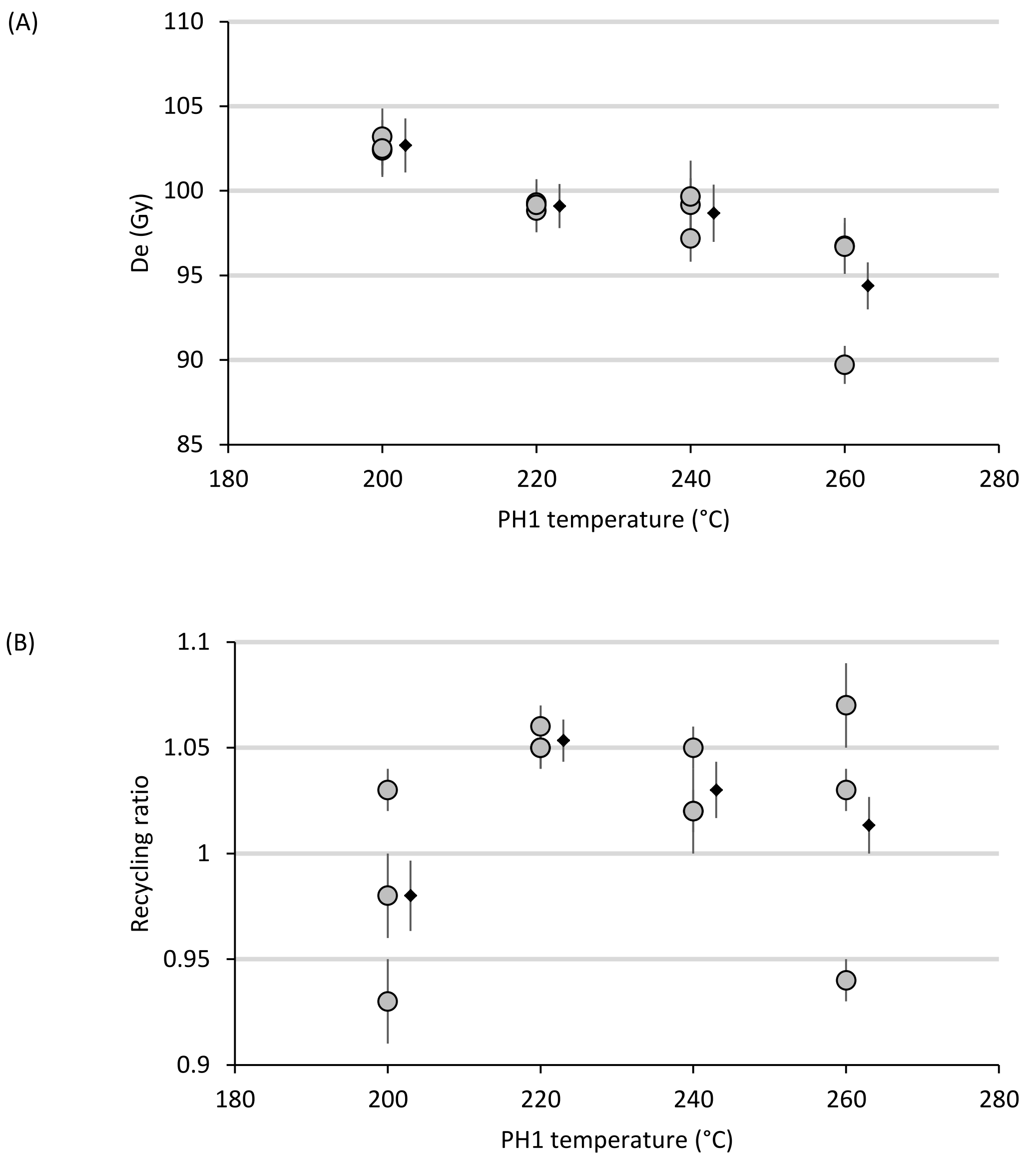

Supplementary Figure 18. Multi-grain dose recovery test results obtained for sample SWC-B after administering a dose of 100 Gy (uncertainties are shown at 1σ). Plots show (A) the recovered doses (grey circles = individual aliquot De values; black circles = weighted mean D_e values) and (B) the recycling ratios obtained for different preheat conditions. D_e measurements were made with a multi-grain version of the AU SAR protocol (replacing single-grain laser stimulations in steps 4 and 7 with blue LED OSL stimulations performed at 125 °C for 10 s) using various regenerative-dose preheat (PH1) and maintaining a test-dose preheat (PH2) of 160 °C for 10 s. D_e measurements were made on multi-grain aliquots containing ~600 quartz grains.

Dose Recovery Test

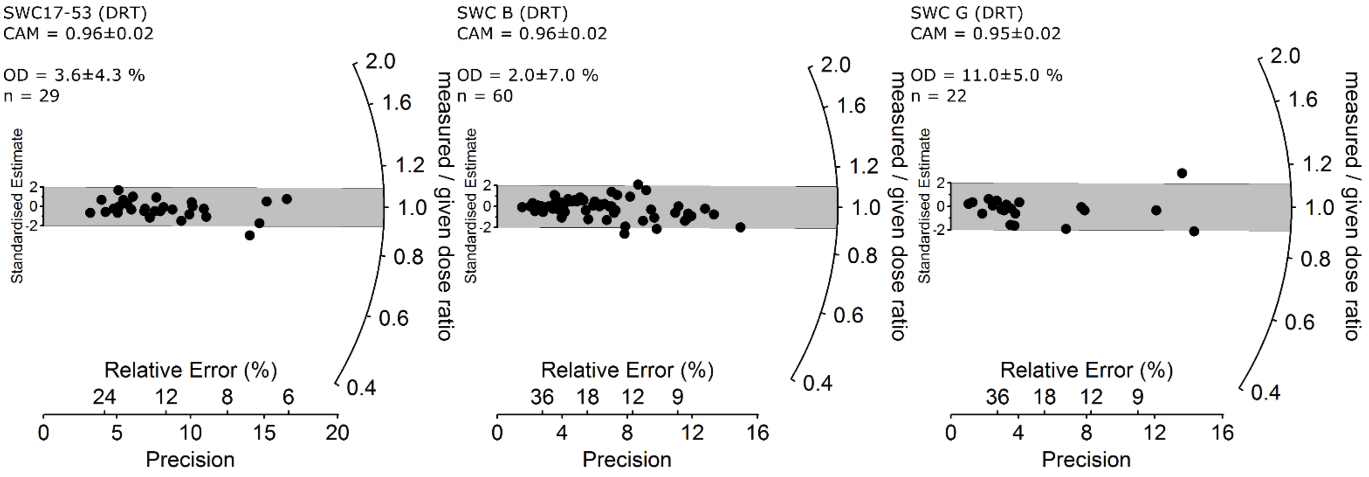

Supplementary Figure 19. Radial plots showing single-grain OSL dose recovery test results obtained for samples SWC17-53, SWC-B and SWC-G using the AU SAR protocol shown in Table S1 (D_e errors are shown at 1σ). Grains were bleached within the Risø reader chamber using blue LEDs prior to administering a dose of 100 Gy. The central age model (CAM) measured-to-given dose ratio and the overdispersion (OD) value is shown for each sample.

SWC-CC

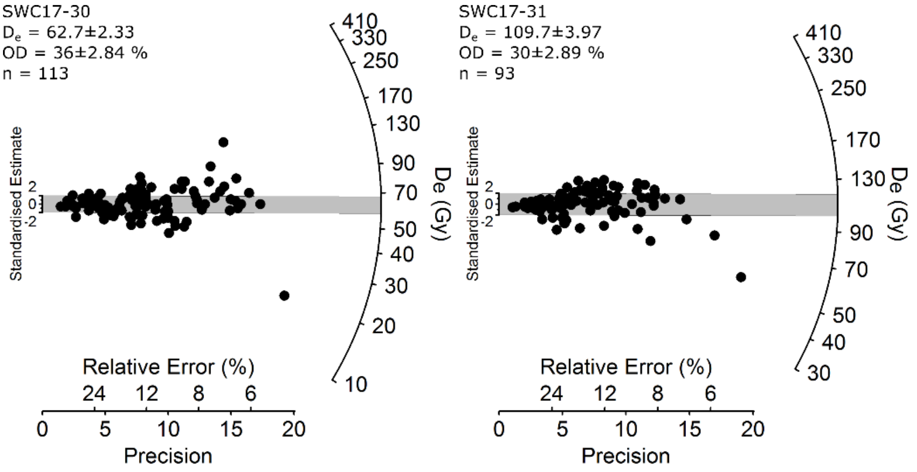

Supplementary Figure 20. Radial plots showing the single-grain OSL D_e distributions obtained for the South Walker Creek, SWC-CC samples (D_e errors are shown at 1σ). The dark grey bands are centred on the weighted mean D_e values, which have been calculated using the CAM.

SWJ

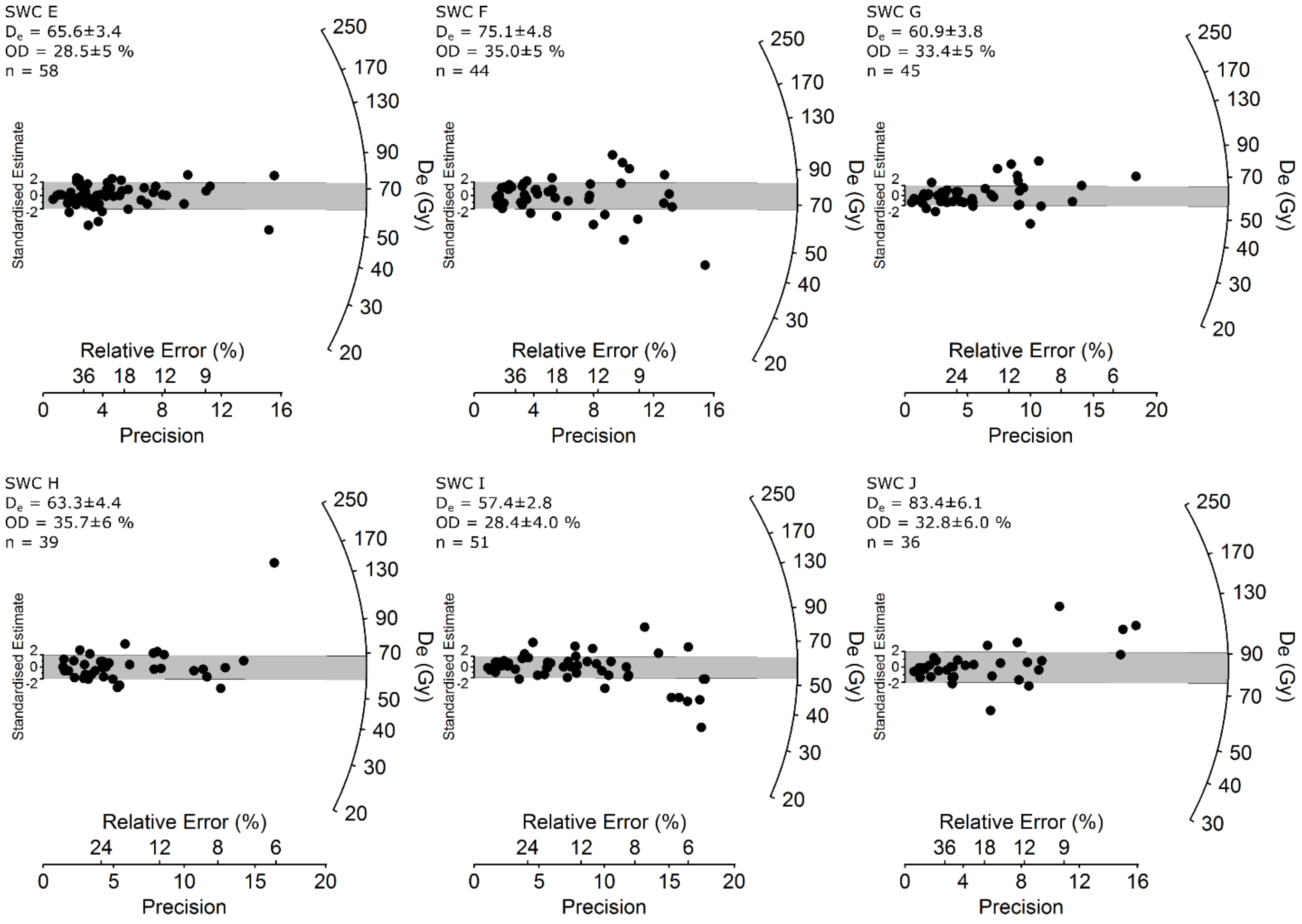

Supplementary Figure 21. Radial plots showing the single-grain OSL D_e distributions obtained for the South Walker Creek, SWJ samples (D_e errors are shown at 1σ). The dark grey bands are centred on the weighted mean D_e values, which have been calculated using the CAM.

SW3

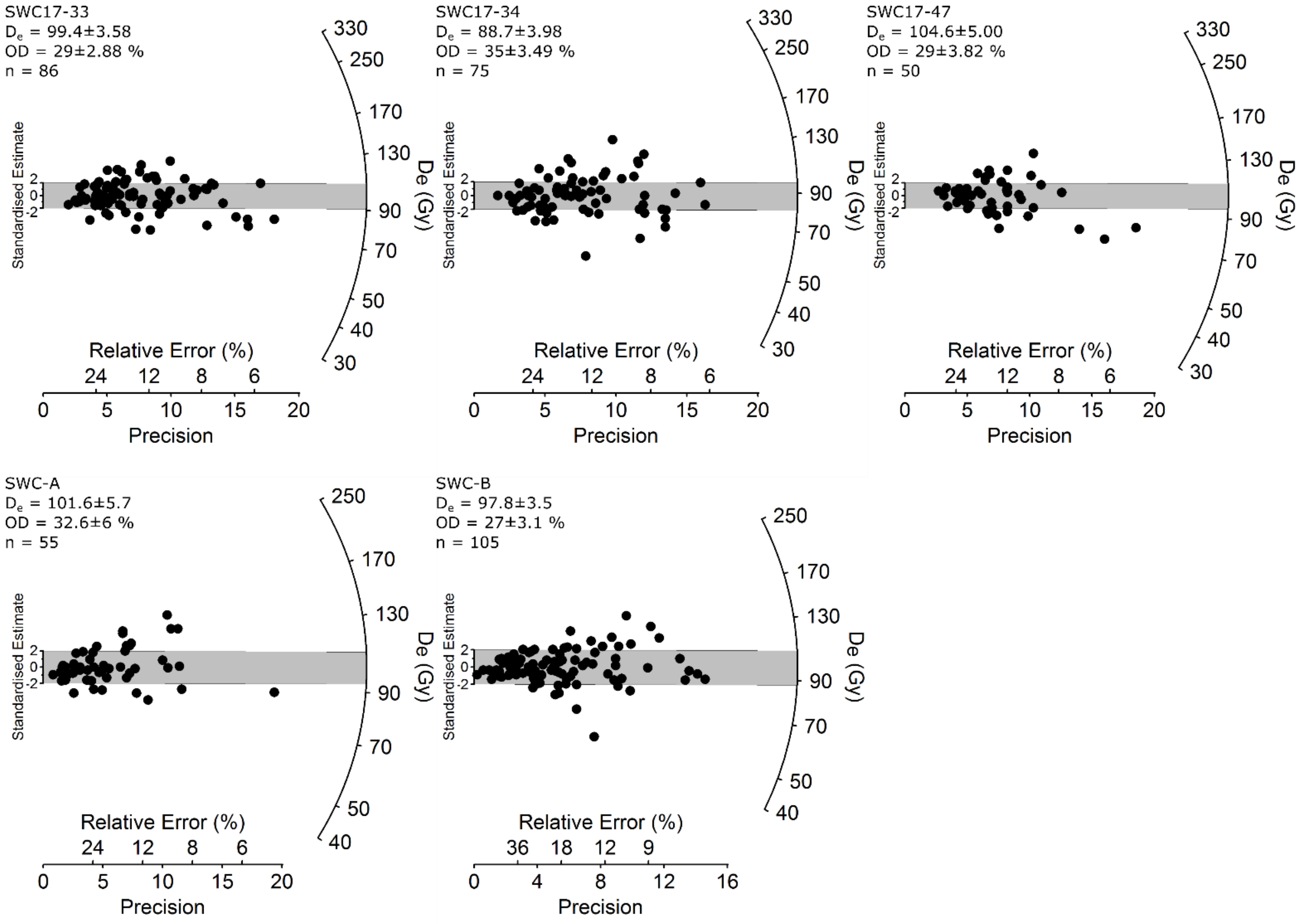

Supplementary Figure 22. Radial plots showing the single-grain OSL D_e distributions obtained for the South Walker Creek, SW3 samples (D_e errors are shown at 1σ). The dark grey bands are centred on the weighted mean D_e values, which have been calculated using the CAM.

SW9 – AU (Unit C – main fossil unit)

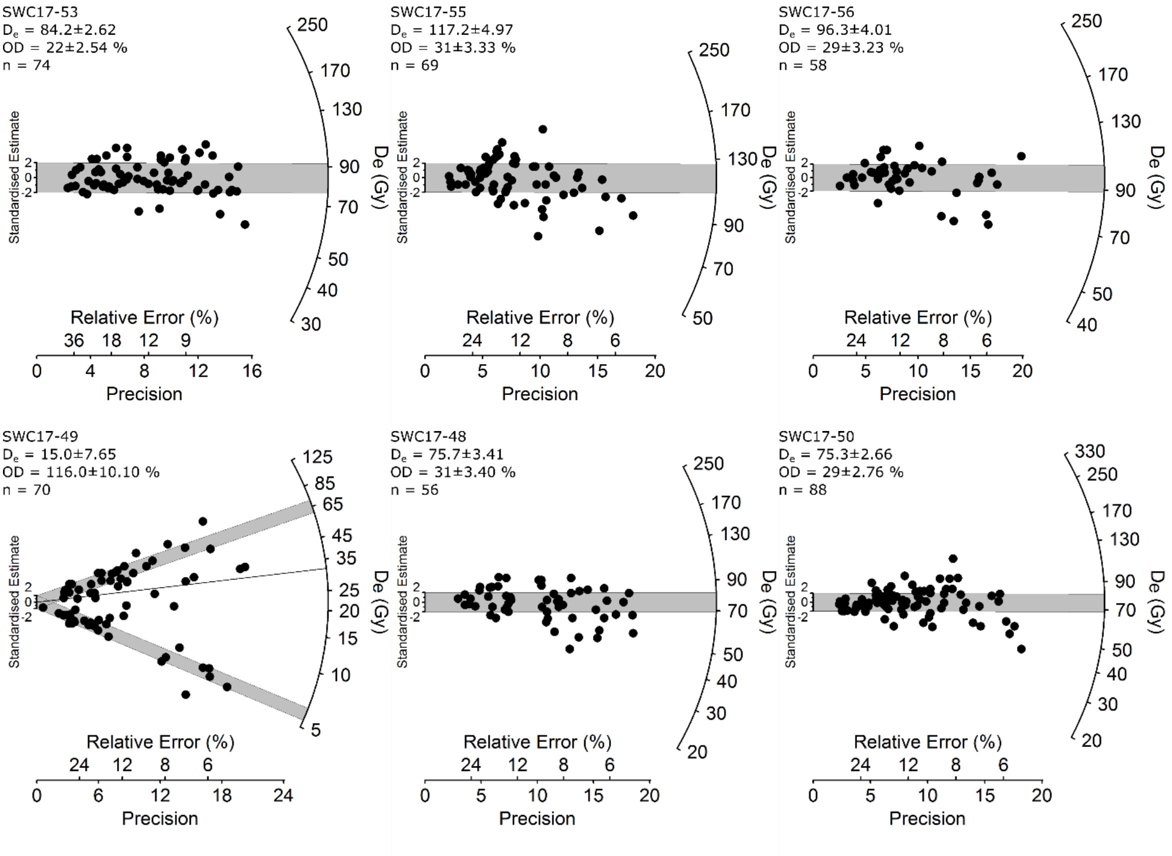

Supplementary Figure 23. Radial plots showing the single-grain OSL D_e distributions obtained for the South Walker Creek, SW9 samples (D_e errors are shown at 1σ) that were taken from the main fossil unit (Unit C; including both sediments in association with fossil remains and those surrounding fossil remains). The dark grey bands are centred on the weighted mean D_e values, calculated using the CAM, or the discrete dose populations identified by the FMM (SWC17-49)..

SW9 – AU (sand lens samples)

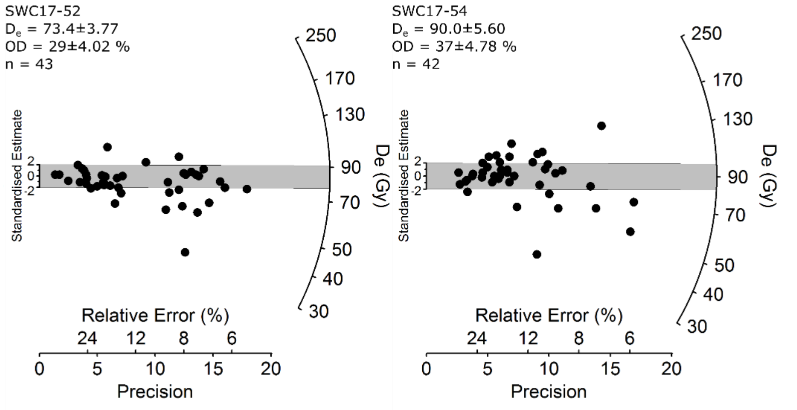

Supplementary Figure 24. Radial plots showing the single-grain OSL D_e distributions obtained for the South Walker Creek, SW9 samples (D_e errors are shown at 1σ) that were extracted from sand intrusions. The dark grey bands are centred on the weighted mean D_e values, which have been calculated using the CAM.

SW9 – GU (Unit C – main fossil unit)

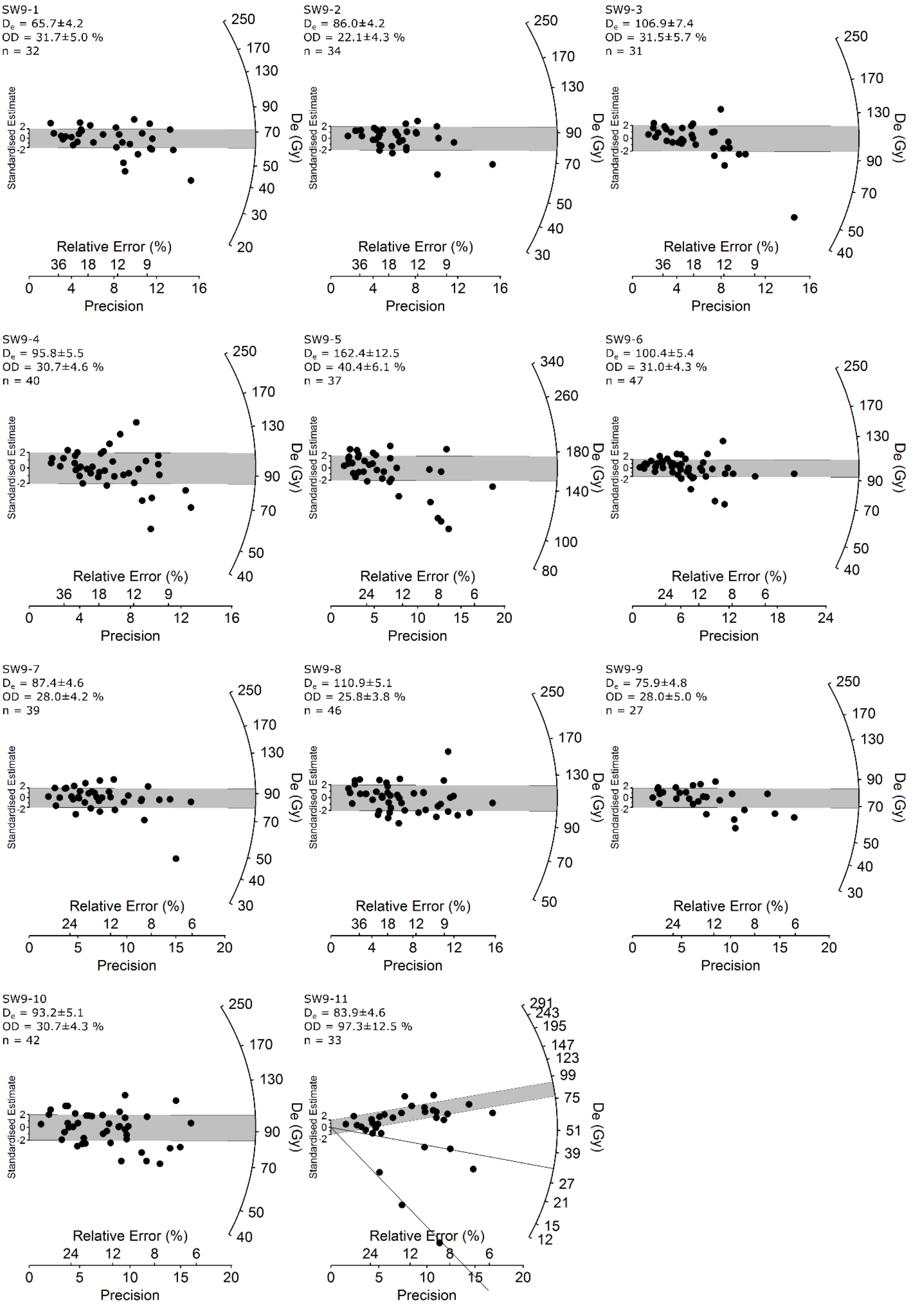

Supplementary Figure 24. Radial plots showing the single-grain OSL D_e distributions obtained for the South Walker Creek, SW9 (GU) samples (D_e errors are shown at 1σ) constraining the vertical and horizontal age distribution of the main fossil unit (Unit C). The dark grey bands are centred on the weighted mean D_e values, calculated using the CAM, or the discrete dose populations identified by the FMM (SW9-11).

Supplementary Table 8. SAR protocols used by the Adelaide University (AU) and Griffith University (GU) laboratories in this study to obtain single-grain OSL ages. Ln and Lx refer to the natural and regenerative-dose signal measurements, respectively. Tn and Tx refer to the test dose signals measured after the Ln and Lx signals, respectively**.**

Step	AU single-grain OSL SAR	Notation	Step	GU single-grain OSL SAR	Notation
1[a]	Give dose		1	Give dose	
2[b]	Stimulate with infrared diodes at 50°C for 40 s (90% power)		2[c]	Stimulate with infrared diodes at 125°C for 40 s (90% power)	
3	Preheat at 240°C for 10 s		3	Preheat at 240°C for 10 s	
4	Stimulate with green laser at 125°C for 2 s (90% power)	L_n or L_x	4	Stimulate with green laser at 125°C for 1 s (90% power)	L_n or L_x
5	Give test dose (5 Gy)		5	Give test dose (5 Gy)	
6	Preheat to 160°C for 10 s		6[c]	Stimulate with infrared diodes at 125°C for 40 s (90% power)	
7	Stimulate with green laser at 125ºC for 2 s (90% power)	T_n or T_x	7	Preheat to 160°C for 10 s	
8	Return to 1		8	Stimulate with green laser at 125°C for 1 s (90% power)	T_n or T_x
			9	Return to 1	

[a] Step omitted when measuring the natural signal (L_n).

[b] Step added only when measuring the OSL-IR depletion ratio described in Duller [66].

[c] Each single-grain disc was stimulated with infrared diodes prior to OSL signal measurement to bleach any IR-sensitive signal.

Supplementary Table 9. Single-grain dose recovery results obtained for 212-250 µm quartz grains of samples SWC17-53, SWC-B and SWC-G using the AU SAR protocol shown in Supplementary Table 8. n = number of accepted De values; OD = overdispersion value.

ID	n	measured-to-given ratio	OD (%)
SWC17-53 (DRT)	29	0.96±0.02	3.6±4.3
SWC B (DRT)	60	0.96±0.02	2.0±6.6
SWC G (DRT)	22	0.95±0.05	10.7±5.4

Supplementary Table 10. Single-grain OSL statistics showing proportion of rejected and accepted grains after applying the SAR rejection criteria to the 2015 and 2017 samples. Data are also shown for the single-grain OSL dose recovery test (DRT) measurements made on samples SWC17-53, SWC-B, SWC-G, (in the first three rows).

			Rejected grains (%)								
Sample	Total Grains (n)	Tn <3σ BG	Poor low dose recycling ratio	Poor high dose recycling ratio	IR depletion ratio	Recuper-ation >5%	Saturated	Anomalous dose response curve	Ln/Tn not intersect DRC	Relative error of D_e >50%	Accepted grains (%)
SWC17-53 SG (DRT)	500	62	14	6	4	2	0	6	0	0	6
SWC17-B SG (DRT)	1000	58	10	5	5	6	1	8	2	0	5
SWC17-G SG (DRT)	300	26	33	12	8	7	1	3	4	0	7
SWC17-30	1400	67	9	6	4	3	0	2	0	0	8
SWC17-31	2100	67	15	5	4	3	0	2	0	0	4
SWC17-47	600	64	16	3	4	2	0	1	1	0	8
SWC17-33	1800	62	17	5	5	3	0	3	1	0	5
SWC17-34	1800	59	20	5	5	3	0	3	0	0	4
SWC17-A	600	33	26	11	6	7	1	3	4	0	9
SWC17-B	1200	52	14	7	5	6	2	4	3	0	9
SWC17-53	1600	68	12	4	5	3	0	3	1	1	5
SWC17-55	800	65	6	5	5	5	0	4	2	1	9
SWC17-56	600	66	11	4	5	2	0	1	2	0	10
SWC17-52	1300	69	15	4	3	2	0	2	0	0	3
SWC17-54	700	64	16	4	5	2	0	1	0	0	6
SWC17-50	900	62	11	5	3	5	0	3	1	1	10
SWC17-48	800	68	8	4	4	6	0	2	1	1	7
SWC17-49	1400	67	14	5	4	4	0	2	0	0	5
SWC17-E	800	60	15	6	4	4	1	2	1	0	7
SWC17-F	500	42	24	10	5	5	0	3	2	0	9
SWC17-G	500	49	17	9	6	5	1	3	1	0	9
SWC17-H	500	52	19	9	5	4	0	2	1	0	8
SWC17-I	1300	68	15	3	5	3	0	2	0	0	4
SWC17-J	700	70	10	4	4	4	0	2	1	0	5

Supplementary Table 11. Dose rate data, single-grain equivalent doses (D_e) and quartz OSL ages for the 2009, 2015 and 2017 SWC samples.

			Environmental dose rate data		Equivalent dose (D_e) data				
LOC	ID	Grain Size (µm)	Water Content (%Dry)[a]	Total Dose Rate (Gy/ka)[b,c]	n[d]	OD (%)[e]	Age Model[f]	D_e (Gy)[c]	Final Age ± 1σ (ka)[g]
SWCC	SWC17-30	212-250	12±2.4	1.25±0.1	113	36±2.8	CAM	62.7±2.3	50.2±3.1
SWCC	SWC17-31	212-250	12±2.4	1.62±0.1	93	30±2.9	CAM	109.7±4.0	67.5±4.2
SW3	SWC17-47	212-250	23±4.7	2.18±0.1	50	29±3.8	CAM	104.6±5.0	48.0±3.8
SW3	SWC17-33	212-250	20±4.0	2.22±0.1	86	29±2.9	CAM	99.4±3.6	44.8±3.1
SW3	SWC17-34	212-250	20±4.0	2.04±0.1	75	35±3.5	CAM	88.7±4.0	43.5±3.2
SW3	SWC-A	212-250	16±3.3	2.03±0.1	55	33±4.6	CAM	101.6±5.7	50.1±3.9
SW3	SWC-B	212-250	31±6.3	1.59±0.1	105	27±3.1	CAM	97.8±3.5	61.5±5.0
SW9	SWC17-53	212-250	26±5.1	2.22±0.1	74	22±2.5	CAM	84.2±2.6	37.9±2.7
SW9	SWC17-55	212-250	24±4.7	2.54±0.1	69	31±3.3	CAM	117.2±5.0	46.1±3.4
SW9	SWC17-56	212-250	19±3.8	2.30±0.1	58	29±3.2	CAM	96.3±4.0	42.0±2.9
SW9	SWC17-52	212-250	19±3.8	1.80±0.1	43	29±4.0	CAM	73.4±3.8	40.7±3.1
SW9	SWC17-54	212-250	21±4.1	1.89±0.1	42	37±4.8	CAM	90.0±5.6	47.5±4.1
SW9	SWC17-50	212-250	10±2.0	1.76±0.1	88	29±2.8	CAM	75.3±2.7	42.8±2.6
SW9	SWC17-48	212-250	12±2.4	1.40±0.1	56	31±3.4	CAM	75.7±3.4	54.2±3.7
SW9	SWC17-49	212-250	20±4.0	1.57±0.1	70	116±10.1	FMM	65.1±5.98	41.6±5.1
SW9	SW9-1	180-212	22±4.0	1.65±0.1	32	32±5.0	CAM	65.7±4.2	39.7±4.3
SW9	SW9-2	180-212	22±4.0	2.02±0.2	34	22±4.3	CAM	86.0±4.2	42.6±4.3
SW9	SW9-3	180-212	22±4.0	2.43±0.2	31	31±5.7	CAM	106.9±7.4	43.9±4.7
SW9	SW9-4	180-212	22±4.0	2.52±0.2	40	31±4.6	CAM	95.8±5.5	38.0±4.2
SW9	SW9-5	180-212	22±4.0	2.37±0.2	37	40±6.1	CAM	162.4±12.5	68.6±8.4
SW9	SW9-6	180-212	22±4.0	2.15±0.2	47	31±4.3	CAM	100.4±5.4	46.6±5.0
SW9	SW9-7	180-212	22±4.0	2.66±0.2	39	28±4.2	CAM	87.4±4.6	32.9±3.4
SW9	SW9-8	180-212	22±4.0	2.62±0.2	46	26±3.8	CAM	110.9±5.1	42.3±4.1
SW9	SW9-9	180-212	22±4.0	2.56±0.2	27	28±5.0	CAM	75.9±4.8	29.7±3.1
SW9	SW9-10	180-212	22±4.0	2.47±0.2	42	31±4.3	CAM	93.2±5.1	37.7±3.6
SW9	SW9-11	180-212	22±4.0	1.85±0.2	33	97±12.5	FMM	83.9±4.6	45.3±5.5
SWJ	SWC-E	212-250	31±6.3	0.99±0.1	58	29±4.6	CAM	65.6±3.4	66.6±6.2
SWJ	SWC-F	212-250	28±5.6	1.24±0.1	44	35±5.2	CAM	75.1±4.8	60.5±5.8
SWJ	SWC-G	212-250	26±5.1	0.91±0.1	45	33±5.2	CAM	60.9±3.8	66.6±6.5
SWJ	SWC-H	212-250	30±6.0	0.97±0.1	39	36±5.6	CAM	63.3±4.4	65.6±6.7
SWJ	SWC-I	212-250	22±4.4	0.77±0.1	51	28±3.8	CAM	57.4±2.8	74.7±6.3
SWJ	SWC-J	212-250	38±7.6	1.39±0.1	36	33±6.1	CAM	83.4±6.1	60.1±6.5

[a] Long-term water content, expressed as % of dry mass of mineral fraction, with an assigned relative uncertainty of ±20%.
[b] Total dose rate components (i.e. gamma, beta, internal and cosmic contributions); the breakdown of individual dose rate components is outlined in Supplementary Table 15.
[c] Mean ± total uncertainty (68% confidence interval), calculated in quadrature as the sum of the random and systematic uncertainties. The FMM D_e values for SWC17-49 and SW9-11 have been calculated using the dominant dose components (those containing the largest proportion of accepted grains), as identified from the optimum FMM fits for these samples (the fit with the lowest BIC score; Arnold and Roberts, 2009), (see Supplementary Table 812 and Supplementary Tables 13 and 14).
[d] Number of D_e measurements that passed the SAR rejection criteria and were used for D_e determination.
[e] OD = overdispersion; the relative spread in the D_e dataset beyond that associated with the measurement uncertainties of individual D_e values, and calculated using the central age model (CAM) of [76].
[f] Age model used to calculate the sample-averaged D_e value for each sample. CAM = central age model [76]; FMM = finite mixture model [78]. The FMM D_e values and ages shown for samples SWC17-49 and SW9-11 are included for indicative purposes only and have not been included in our final age evaluations for SW9.
[g] Total uncertainty includes a systematic component of ±2% associated with laboratory beta-source calibration.

Supplementary Table 12. Log likelihood (llik) statistics[98, 99] associated with the of De populations of the South Walker Creek samples. The minimum age model was calculated using an assumed overdispersion of 20% based on sample SW9-2, considered 'ideal' for representing expected beta dose heterogeneity within local sediments[69] . The llik values shown in bold indicate the most statistically suitable age model fit for each De dataset**.**

LOC	ID	CAM llik [c]	MAM-3 llik [c,d]	MAM-4 [c,d]
SWCC	SWC17-30	**-55.4**	-59.2	-56.1
SWCC	SWC17-31	**-35.2**	-38.2	-31.5
SW3	SWC17-47	**-15.8**	-15.6	-15.0
SW3	SWC17-33	**-30.3**	-30.6	-30.3
SW3	SWC17-34	**-35.9**	-37.9	-35.2
SW3	SWC A	**-32.0**	-32.0	-30.8
SW3	SWC B	**-48.5**	-49.9	-46.4
SW9	SWC17-53	**-11.5**	-11.3	-11.2
SW9	SWC17-55	**-25.6**	-26.1	-25.4
SW9	SWC17-56	**-7.7**	-7.9	-7.3
SW9	SWC17-52	**-14.7**	-15.6	-13.5
SW9	SWC17-54	**-20.8**	-25.3	-18.8
SW9	SWC17-50	**-28.8**	-29.0	-28.7
SW9	SWC17-48	**-19.0**	-19.3	-18.9
SW9	SWC17-49[a]	-114.9	-107.4	-714.5
SW9	SW9-1	**-14.5**	-15.3	-14.1
SW9	SW9-2	**-6.3**	-6.4	-6.2
SW9	SW9-3	**-15.3**	-16.1	-14.8
SW9	SW9-4	**-16.1**	-16.2	-16.1
SW9	SW9-5	**-26.5**	-26.3	-25.7
SW9	SW9-6	**-20.9**	-21.9	-20.0
SW9	SW9-7	**-12.6**	-13.7	-10.8
SW9	SW9-8	**-17.1**	-16.6	-16.1
SW9	SW9-9	**-9.6**	-9.6	-9.2
SW9	SW9-10	**-20.1**	-19.8	-19.7
SW9	SW9-11[a]	-180.3	-62.0	-161.7
SWJ	SWC E	**-37.6**	-38.4	-35.7
SWJ	SWC F	**-26.3**	-27.4	-25.3
SWJ	SWC G	**-31.1**	-30.9	-30.8
SWJ	SWC H	-23.7	**-21.6**	-21.1
	SWC H [b]	**-18.3**	-18.2	-18.2
SWJ	SWC I	**-19.4**	-19.1	-19.1
SWJ	SWC J	**-22.9**	-23.8	-22.1

[a] Designates samples exhibiting complex D_e distributions with very high overdispersion values of ~100%, and which were subsequently fitted using the finite mixture model (FMM).

[b] Comparative age model llik scores calculated for sample SWC H after elimination of a single, precise, high outlier D_e value of 182 ± 11 Gy (Supplementary Table 11).

[c] Llik represents the maximum log likelihood score of the CAM, MAM-3 or MAM-4 fit. For a given sample, the llik score of the MAM-3 is expected to be substantially higher (i.e. at least 1.92 greater) than that of the CAM when the addition of the extra model parameter improves the fit to the data. Likewise, the llik score of the MAM-4 is expected to be significantly greater than that of the MAM-3 (by at least 1.92 when compared with the 95% C.I. of a X^2 distribution) when the addition of the extra model parameter improves the fit to the data. If the extra parameter of the MAM-3 (or MAM-4) is not supported by the data, then its llik score will be similar to (i.e. within 1.92 of) the CAM (or MAM-3) llik score, indicating that the simpler age model explains the data equally well [79].

[d] MAM-3 and MAM-4 D_e estimates were calculated after adding, in quadrature, a relative error of 20% to each individual D_e measurement error to approximate the underlying dose overdispersion observed in an 'ideal' (well-bleached and unmixed) sedimentary sample from SWC (SWC17-53 and SW9-2), which is consistent with global overdispersion datasets [69]).

Supplementary Table 13. Finite mixture model fitting results for sample SW9-11, which exhibits a complex De distribution with multiple dose components. The FMM was fitted by varying the common overdispersion (OD) parameter between 10 and 30% and incrementally increasing the specified number of dose components until the FMM fits would no longer converge. The Bayes Information Criteria (BIC) and log-likelihood (llik) scores are shown for each of the different FMM parameterisation scenarios tested with sample SW9-11. The BIC score has been used to assess the suitability of the FMM fits, with the lowest BIC score taken to represent the optimum parameterisation of the FMM (shown in bold). The grey text identifies FMM parameterisation scenarios that are deemed empirically unsupported, since the specified common overdispersion value is smaller than that observed for 'ideal' (well-bleached and unmixed) sedimentary sample from SWC (SWC17-53 and SW9-2).

**SW9-11**									
**OD**	**# comps**	**llik**	**BIC**	**comp 1 (Gy)**	**proportion of grains**	**comp 2 (Gy)**	**proportion of grains**	**comp 3 (Gy)**	**proportion of grains**
10	1	-570.22	1143.93	50.78					
10	2	-109.87	230.24	3.23±0.31	0.09±0.05	67.59±2.05	0.91±0.05		
10	3	-31.21	79.91	3.23±0.31	0.09±0.05	30.53±2.14	0.18±0.07	83.68±2.94	0.73±0.08
15	1	-367.80	739.10	50.9					
15	2	-69.17	148.84	3.28±0.38	0.09±0.05	68.47±2.61	0.91±0.05		
15	3	-26.99	71.47	3.28±0.38	0.09±0.05	31.44±2.83	0.18±0.07	84.09±3.76	0.72±0.08
20	1	-250.36	504.21	51.15					
20	2	-49.17	108.82	3.31±0.46	0.09±0.05	68.89±3.19	0.91±0.05		
**20**	**3**	**-26.04**	**69.57**	**3.31±0.46**	**0.09±0.05**	**32.08±3.62**	**0.18±0.08**	**83.91±4.62**	**0.73±0.08**
25	1	-180.32	364.14	51.35					
25	2	-39.11	88.72	3.34±0.55	0.09±0.05	69.12±0.05	0.91±0.05		
25	3	-26.61	70.69	3.34±0.55	0.09±0.05	32.58±6.63	0.17±0.08	83.26±5.54	0.74±0.09
30	1	-136.70	276.90	51.56					
30	2	-34.08	78.65	3.36±0.64	0.09±0.05	69.28±4.36	0.91±0.05		
30	3	-27.75	72.98	3.36±0.64	0.09±0.05	33.16±6.08	0.16±0.08	82.10±6.52	0.75±0.09

Supplementary Table 14. Finite mixture model fitting results for sample SWC17-49, which exhibits a complex De distribution with multiple dose components. The FMM was fitted by varying the common overdispersion (OD) parameter between 10 and 30% and incrementally increasing the specified number of dose components until the FMM fits would no longer converge. The Bayes Information Criteria (BIC) and log-likelihood (llik) scores are shown for each of the different FMM parameterisation scenarios tested with sample SWC17-49. The BIC score has been used to assess the suitability of the FMM fits, with the lowest BIC score taken to represent the optimum parameterisation of the FMM (shown in bold). The grey text identifies FMM parameterisation scenarios that are deemed empirically unsupported, since the specified common overdispersion value is smaller than that observed for 'ideal' (well-bleached and unmixed) sedimentary sample from SWC (SWC17-53 and SW9-2).

**SWC17-49**									
					**proportion**		**proportion**		**proportion**
**OD**	**# comps**	**llik**	**BIC**	**comp 1 (Gy)**	**of grains**	**comp 2 (Gy)**	**of grains**	**comp 3 (Gy)**	**of grains**
10	1	-1519.07	3042.40	22.57					
10	2	-212.50	437.75	6.07±0.19	0.44±0.06	51.20±0.56	0.60±0.06		
10	3	-118.45	258.14	6.08±0.20	0.440.06	32.88±1.36	0.22±0.05	72.00±2.58	0.34±0.06
15	1	-961.65	1927.54						
15	2	-145.60	303.94	6.10±0.25	0.44±0.06	51.87±1.67	0.56±0.06		
15	3	-96.71	214.65	6.09±0.25	0.44±0.06	32.76±1.95	0.21±0.05	71.35±3.40	0.35±0.06
20	1	-662.74	1329.73	22.17					
20	2	-113.44	239.62	6.09±0.30	0.44±0.06	52.35±2.08	0.56±0.06		
20	3	-87.91	197.06	6.057±0.30	0.44±0.06	32.66±2.73	0.21±0.06	70.41±0.06	4.32±0.06
25	1	-486.53	977.32	21.87					
25	2	-97.75	208.25	6.08±0.36	0.44±0.06	52.77±2.52	0.56±0.06		
25	3	-84.96	191.16	6.02±0.35	0.44±0.06	32.51±3.88	0.19±0.07	68.62±5.52	0.37±0.07
30	1	-375.07	754.38	21.63					
30	2	-90.36	193.46	6.08±0.42	0.44±0.06	53.16±2.98	0.56±0.06		
**30**	**3**	**-84.56**	**190.36**	**5.98±0.40**	**0.44±0.06**	**31.69±6.30**	**0.16±0.09**	**65.16±7.12**	**0.40±0.10**

Supplementary Table 15. Environmental dose rate values calculated for the quartz fractions of the 2011, 2015 and 2017 OSL samples.

				Environmental dose rate (Gy/ka)				
LOC	ID[d]	Depth (cm)	W_c (%Dry)[a]	Beta [b, c]	Gamma [c, d]	Internal [c, e]	Cosmic [c, f]	Total [c]
SWCC	SWC17-30	92	12±2.4	0.54±0.03[β]	0.50±0.02 [FGS]	0.03±0.01	0.17±0.02	1.25±0.06
SWCC	SWC17-31	78	12±2.4	0.73±0.04[β]	0.69±0.02 [FGS]	0.03±0.01	0.17±0.02	1.62±0.07
SW3	SWC17-47	194	23±4.7	1.21±0.06[β]	0.80±0.03 [FGS]	0.03±0.01	0.13±0.01	2.18±0.13
SW3	SWC17-33	194	20±4.0	1.220±0.06[β]	0.83±0.03 [FGS]	0.03±0.01	0.14±0.01	2.22±0.12
SW3	SWC17-34	263	20±4.0	1.154±0.06[β]	0.72±0.02 [FGS]	0.03±0.01	0.13±0.01	2.04±0.11
SW3	SWC A	95	16±3.3	1.02±0.04 [HRGS]	0.81±0.03[HRGS]	0.03±0.01	0.16±0.02	2.03±0.10
SW3	SWC B	155	31±6.3	0.80±0.04 [HRGS]	0.62±0.03 [HRGS]	0.03±0.01	0.13±0.01	1.59±0.13
SW9	SWC17-53	252	26±5.1	1.13±0.06[β]	0.93±0.02 [FGS]	0.03±0.01	0.12±0.01	2.22±0.13
SW9	SWC17-55	260	24±4.7	1.30±0.07[β]	1.08±0.03 [FGS]	0.03±0.01	0.12±0.01	2.54±0.14
SW9	SWC17-56	245	19±3.8	1.18±0.06[β]	0.95±0.02 [FGS]	0.03±0.01	0.13±0.01	2.30±0.12
SW9	SWC17-52	230	19±3.8	0.86±0.04[β]	0.77±0.02 [FGS]	0.03±0.01	0.13±0.01	1.80±0.10
SW9	SWC17-54	230	21±4.1	1.00±0.05[β]	0.73±0.02 [FGS]	0.03±0.01	0.13±0.01	1.89±0.11
SW9	SWC17-50	30	10±2.0	0.85±0.04[β]	0.68±0.02 [FGS]	0.03±0.01	0.19±0.02	1.76±0.08
SW9	SWC17-48	100	12±2.4	0.67±0.03[β]	0.52±0.02 [FGS]	0.03±0.01	0.17±0.02	1.40±0.07
SW9	SWC17-49	64	20±4.0	0.72±0.04[β]	0.65±0.02[FGS]	0.03±0.01	0.16±0.02	1.57±0.09
SW9	SW9-1	120	22±4.0	0.87±0.08 [HRGS]	0.62±0.05 [HRGS]	0.03±0.01	0.13±0.01	1.65±0.14
SW9	SW9-2	140	22±4.0	1.12±0.01 [HRGS]	0.74±0.06 [HRGS]	0.03±0.01	0.13±0.01	2.02±0.17
SW9	SW9-3	160	22±4.0	1.36±0.11 [HRGS]	0.91±0.06 [HRGS]	0.03±0.01	0.13±0.01	2.43±0.19
SW9	SW9-4	180	22±4.0	1.40±0.13 [HRGS]	0.96±0.08 [HRGS]	0.03±0.01	0.13±0.01	2.52±0.24
SW9	SW9-5	200	22±4.0	1.34±0.13 [HRGS]	0.87±0.07 [HRGS]	0.03±0.01	0.13±0.01	2.37±0.22
SW9	SW9-6	200	22±4.0	1.20±0.11 [HRGS]	0.79±0.07 [HRGS]	0.03±0.01	0.13±0.01	2.15±0.20
SW9	SW9-7	200	22±4.0	1.50±0.13 [HRGS]	1.00±0.08 [HRGS]	0.03±0.01	0.13±0.01	2.66±0.23
SW9	SW9-8	200	22±4.0	1.49±0.12 [HRGS]	0.97±0.07 [HRGS]	0.03±0.01	0.13±0.01	2.62±0.22
SW9	SW9-9	200	22±4.0	1.46±0.12 [HRGS]	0.94±0.06 [HRGS]	0.03±0.01	0.13±0.01	2.56±0.20
SW9	SW9-10	200	22±4.0	1.38±0.10 [HRGS]	0.93±0.06 [HRGS]	0.03±0.01	0.13±0.01	2.47±0.19
SW9	SW9-11	150	22±4.0	1.03±0.11 [HRGS]	0.66±0.07 [HRGS]	0.03±0.01	0.13±0.01	1.85±0.20
SWJ	SWC E	20	31±6.3	0.44±0.02 [HRGS]	0.36±0.02 [HRGS]	0.03±0.01	0.15±0.02	0.99±0.08
SWJ	SWC F	50	28±5.6	0.60±0.03 [HRGS]	0.46±0.03 [HRGS]	0.03±0.01	0.15±0.02	1.24±0.10
SWJ	SWC G	80	26±5.1	0.40±0.02 [HRGS]	0.33±0.02 [HRGS]	0.03±0.01	0.15±0.02	0.91±0.07

SWJ	SWC H	120	30±6.0	0.45±0.02 HRGS	0.35±0.02 HRGS	0.03±0.01	0.14±0.01	0.97±0.08
SWJ	SWC I	150	22±4.4	0.32±0.02 HRGS	0.27±0.02 HRGS	0.03±0.01	0.14±0.01	0.77±0.06
SWJ	SWC J	180	38±7.6	0.77±0.04 HRGS	0.46±0.02 HRGS	0.03±0.01	0.12±0.01	1.39±0.11

[a] Long-term water content, expressed as % of dry mass of mineral fraction, with an assigned relative uncertainty of ±20%.
[b] Beta dose rates were calculated on dried, powdered sediment samples using a Risø GM-25-5 low level beta counting ($^{\beta}$) or, high resolution gamma spectroscopy (^{HRGS}) after making allowance for beta dose attenuation due to grain-size effects and HF etching[92, 94] .
[c] Mean ± total uncertainty (68% confidence interval), calculated as the quadratic sum of the random and systematic uncertainties.
[d] Gamma dose rates were calculated from *in situ* gamma-ray spectrometry measurements made at each sample position with a NaI:Tl detector, using the 'energy windows' approach (e.g., Arnold, Duval [80]) $^{(FGS)}$ where possible and; high resolution gamma spectroscopy on dried powdered sediment $^{(HRGS)}$ using the conversion factors given in Stokes et al.[100] and Guerin et al.[90] .
[e] an assumed internal dose rate of 0.03 Gy / ka, with an assigned relative uncertainty of ±30% (±0.01 Gy/ka) is included for each sample, based on published intrinsic ^{238}U and ^{232}Th contents[84, 87, 101, 102] and an alpha efficiency factor (*a*-value) of 0.04 ± 0.01[88, 89].
[f] Cosmic-ray dose rates were calculated using the approach of Prescott and Hutton [83] and assigned a relative uncertainty of ±10%.

Supplementary Table 16. High-resolution gamma spectrometry (HRGS) results for the OSL samples from South Walker Creek. The specific activities of 238U, 226Ra, 210Pb, 228Ra, 228Th and 40K were measured for each sediment sample, and used to calculate daughter-to-parent isotope ratios for 226Ra:238U, 210Pb:226Ra and 228Th:228Ra. Radionuclide specific activities and daughter-to-parent isotopic ratios are shown with their associated 1σ uncertainty ranges.

Sample	Radionuclide specific activities (Bq / kg)						Daughter-to-parent isotopic ratios		
	^{238}U	^{226}Ra	^{210}Pb	^{228}Ra	^{228}Th	^{40}K	^{226}Ra:^{238}U	^{210}Pb:^{226}Ra	^{228}Th:^{228}Ra
SWC17-30	13±1.3	13±0.3	13±1.4	19±0.7	19±0.5	146±5	1.0±0.1	0.9±0.1	1.0±0.05
SWC17-31	18±2.2	21±0.5	19±2.7	25±1.0	25±0.7	211±7	1.1±0.1	0.9±0.1	1.0±0.05
SWC17-47	30±1.6	31±0.5	29±2.0	40±0.9	40±0.8	412±10	1.0±0.1	0.9±0.1	1.0±0.03
SWC17-33	23±1.7	23±0.4	22±2.0	41±1.2	40±0.9	405±11	1.0±0.1	1.0±0.1	1.0±0.04
SWC17-34	25±2.7	24±0.5	24±3.3	38±1.3	40±1.0	385±11	1.0±0.1	1.0±0.1	1.0±0.04
SWC-A	24±1.5	22±0.5	24±2.0	35±1.0	36±0.8	352±10	0.9±0.1	1.1±0.1	1.0±0.04
SWC-B	25±1.9	27±0.5	26±2.3	34±0.9	34±0.8	298±8	1.1±0.1	1.0±0.1	1.0±0.03
SWC17-53	25±2.7	27±0.6	25±3.8	38±1.3	39±1.0	382±11	1.1±0.1	0.9±0.1	1.0±0.04
SWC17-55	20±2.8	22±0.5	21±2.7	43±1.5	43±1.1	481±14	1.1±0.2	1.0±0.1	1.0±0.04
SWC17-56	20±1.5	19±0.4	20±2.2	44±1.1	44±1.0	418±10	1.0±0.1	1.1±0.1	1.0±0.03
SWC17-52	20±1.4	21±0.4	21±2.4	31±0.9	30±0.7	248±7	1.1±0.1	1.0±0.1	1.0±0.04
SWC17-54	24±1.6	24±0.5	24±2.6	32±0.9	31±0.8	313±9	1.0±0.1	1.0±0.1	1.0±0.04
SWC17-50	25±1.9	24±0.5	24±2.8	31±1.0	32±0.8	225±7	1.0±0.1	1.0±0.1	1.0±0.04
SWC17-48	13±1.5	15±0.3	13±1.4	21±0.7	20±0.5	197±6	1.1±0.1	0.9±0.1	1.0±0.04
SWC17-49	20±1.6	19±0.4	19±1.9	31±1.0	31±0.8	190±6	0.9±0.1	1.0±0.1	1.0±0.04
SW9-1	19±6.0	25±2.0	26±6.0	26±2.0	-	320±30	1.3±0.4	1.0±0.2	-
SW9-2	27±7.0	17±1.0	15±6.0	38±3.0	-	440±40	0.6±0.2	1.1±0.5	-

SW9-3	27±1.0	23±2.0	23±1.0	44±5.0	-	540±50	0.9±0.1	1.0±0.1	-
SW9-4	30±10.0	23±2.0	30±10.0	44±4.0	-	540±50	0.8±0.3	0.8±0.3	-
SW9-5	30±10.0	22±2.0	22±8.0	41±4.0	-	530±50	0.7±0.3	1.0±0.4	-
SW9-6	32±9.0	22±2.0	18±8.0	39±4.0	-	470±40	0.7±0.2	1.2±0.6	-
SW9-7	40±10.0	26±2.0	29±9.0	47±4.0	-	570±50	0.7±0.2	0.9±0.3	-
SW9-8	33±8.0	28±2.0	21±7.0	47±4.0	-	600±50	0.8±0.2	1.3±0.5	-
SW9-9	31±7.0	24±2.0	22±6.0	44±3.0	-	590±50	0.8±0.2	1.1±0.3	-
SW9-10	37±7.0	41±2.0	29±6.0	43±3.0	-	520±40	1.1±0.2	1.4±0.3	-
SW9-11	30±10.0	18±2.0	17±9.0	31±3.0	-	400±40	0.6±0.2	1.1±0.6	-
SWC-E	15±1.5	13±0.4	13±1.9	21±0.9	20±0.6	163±6	0.8±0.1	1.±0.2	1.1±0.05
SWC-F	19±1.8	19±0.4	17±2.3	27±0.9	28±0.7	214±7	1.0±0.1	0.9±0.1	1.0±0.04
SWC-G	11±1.5	11±0.3	11±1.9	16±0.8	18±0.6	143±5	1.0±0.1	1.0±0.2	0.9±0.05
SWC-H	12±1.3	13±0.3	15±1.7	18±0.7	20±0.5	162±5	1.1±0.1	1.2±0.1	0.9±0.05
SWC-I	11±1.5	10±0.3	10±1.3	11±0.5	12±0.4	109±4	0.9±0.1	1.1±0.1	1.0±0.06
SWC-J	22±1.4	22±0.4	22±2.0	30±0.9	29±0.7	335±9	1.0±0.1	1.0±0.1	1.0±0.01

Supplementary Table 17. Weighted mean ages for OSL samples collected from the fossil-bearing and associated non-fossiliferous sediment of the each site.

LOC	Samples (n=)	Age ± 1σ (ka)
SWC-CC	2	58.2±6.1
SW3	5	47.7±3.2
SWJ[a]	-	-
SW9[b]	19	41.3±1.9
SW9 (AU)	8	43.3±1.8
SW9 (GU)	11	38.9±3.0

[a] The 6 OSL ages for SWJ have all been derived from Unit B, which underlies the main fossil unit. It is therefore not possible to calculate a weighted mean OSL age for the main fossil unit at SWJ, though the underlying unit yields a weighted mean OSL age of 65.6 ± 2.2 ka (n=6).
[b] Combined results from SW9 (AU) and SW9 (GU) samples.

Supplementary Table 18. Details of the OSL samples used to calculate the weighted mean ages for the fossil units at each site, as shown in Supplementary Table 17.

LOC	Sample(s)
SWC-CC	SWC17-30, SWC17-31
SW3	SWC17-47, SWC17-33, SWC17-34, SWC-A, SWC-B
SWJ	Main fossil unit = none
	Underlying unit = SWC-E, SWC-F, SWC-G, SWC-H, SWC-I, SWC-J
SW9 (AU)	SWC17-53, SWC17-55, SWC17-56, SWC17-52, SWC17-54, SWC17-50, SWC17-48, SWC17-49.
SW9 (GU)	SW9-1, SW9-2, SW9-3, SW9-4, SW9-5, SW9-6, SW9-7, SW9-8, SW9-9, SW9-10, SW9-11

Supplementary Note 6: Radiocarbon Dating

Rachel Wood & Scott A. Hocknull

Bones and teeth from the megafauna were submitted to the Australian National University (ANU) and Beta-analytic radiocarbon facilities. At the ANU, the %N was measured in two bones from SW9 using EA-IRMS (following Brock et al.[103]). The majority of nitrogen within a bone is contained in the protein extracted for dating called collagen. Theoretically, 0.2 %N is required to recover the 1% collagen required for radiocarbon dating, although in practice 0.5 – 0.7 %N is often required[103]. Therefore, values of 0.1 %N in SW9-W5 (*Diprotodon optatum* femur) and 0.0 %N in SW9-82 (*Macropus* sp. (giant) tibia) suggest that insufficient collagen is preserved for radiocarbon dating. This result was supported by unsuccessful attempts to recover collagen at the Beta-analytic radiocarbon facility.

Bone, teeth and charcoal were submitted to Beta Analytic for radiocarbon dating and pretreated using their standard physical and chemical methods for retrieving collagen from bones and teeth and carbon from charcoal or organic sediments (https://www.radiocarbon.com/pretreatment-carbon-dating.htm). No collagen or dateable charcoal was recovered from any sample similar to the ANU result. Organic sediment was then attempted. The bulk organic fraction was collected by sieving to <180 microns to remove roots and macrofossils, and acid washing to remove carbonates. This fraction of sediment contains carbon from a large variety of sources. Each may have a different ^{14}C age. For example, microcharcoal or coal/lignin from sediment eroded into the river may be older than deposition of the sediment, whilst alkali soluble and mobile humic acids derived from degraded plant material may be younger[104, 105, 106]. As a result, age estimates on the bulk organic (Supplementary Table 19) fraction rarely date the deposition of the sediment so we have excluded these results from subsequent age interpretation.

Given the exceptionally young age of the bulk organic fraction of the sediment and doubts regarding its reliability for age control, S.H. searched for macro-charcoal from within and below the fossil bone bed and any macro-charcoal associated with bone remains. All samples were collected using methods to limit contamination. When sampled in the field, samples were placed into clean foil with surrounding sediment. In the laboratory, spatulas or tweezers were used to remove pieces and place directly into a plastic bag. Although many samples were recovered over the course of excavations, few proved suitable for dating, or survived pre-treatments. All macro-charcoal remains were tiny in size, being similar in size to the clasts surrounding them. The largest pieces were recovered from clay-rich pieces of matrix that likely derived as rip up clasts from older sediment. Unfortunately clear

sedimentary structures are not easily defined in the matrix of the SW9 bone-bed; therefore, we cannot be conclusive about the primary nature of the macro-charcoal. It is suspected that these macro-charcoal remains are reworked from older fluvial sediments.

Supplementary Figure 15 indicates the location of samples that returned a date. SW9-13-42 was recovered from directly within the bone-bed close to the main concentration of fossils, whilst SW9-16-C02 and SW9-16-C04 were recovered from within the base of the bone bed below any vertebrate remains, just above an unconformity.

Six samples of charcoal were submitted for dating at the Australian National University. One sample contained enough material to attempt ABOx-SC[107, 108], but the sample completely dissolved during pretreatment. After removing visible contaminants (only sediment) with a scalpel where possible, all remaining samples were gently crushed and pretreated with an ABA protocol involving 1 M HCl (30 min, 70°C), 1 M NaOH (1 h, 70°C, replaced until colourless) and 1 M HCl (30 min, 70°C). After each treatment the sample was rinsed in ultrapure water, and after NaOH treatment was rinsed in ultrapure water until the solution remained colourless. Cleaned material was combusted in an evacuated sealed quartz tube with CuO wire and Ag foil. The CO_2 generated was cryogenically collected and purified prior to graphitisation over an Fe catalyst with H_2. %C was measured volumetrically during CO_2 collection. Samples were dated on a NEC single stage AMS [109], and dates calculated using a $\delta^{13}C$ measured by AMS and according to Stuiver and Polach[110]. Dates were calibrated against SHCal13[111] in OxCal[112].

Three of five charcoal samples survived the ABA pretreatment. Only one sample is a single entity. The other two consist of multiple small pieces of charcoal. Sample SW9-13-42a (S-ANU40224) had a low % yield, suggesting poor preservation and/ or low firing temperature. It was also <50% C after pretreatment, suggesting that sediment grains were present in the dated sample. All samples, and particularly SW9-13-42a (S-ANU40224), must be considered a minimum age as the ABA pretreatment does not always remove sufficient contaminants to produce an accurate age estimate. Given the extreme sensitivity of samples beyond 40 ka to young contamination, if inaccurate, they are most likely erroneously young. Radiocarbon dates on charcoal from fluvial sediments must be older than the deposition of the sediment, and this effect may be up to several thousand years[113, 114, 115].

Taken together, the macro-charcoal at SW9 is older than 40 cal kBP. However, it is most likely that it dates to beyond the limit of radiocarbon dating. This is rather uninformative, implying that the

sediment was deposited after 40 cal kBP, and quite possibly after > 50 cal kBP. Reworking of the macro-charcoal within rip up clasts could explain this greater age in comparison with other methods.

Supplementary Table 19. Radiocarbon Dates. Calibration is against SHCal13 (Hogg et al. 2013) in OxCal (Ramsey 2009). * Date may extend beyond calibration curve.

**Laboratory Code**	**Sample Name**	**Number of charcoal pieces**	**$F^{14}C$**	**%C**	**% yield**	**IRMS $\delta^{13}C$**	**^{14}C age (BP)**	**Calibrated age (95% probability)**
Sediment								
Beta-359939	CC15	1 (not recovered)	0.1420 ± 0.0012			-20.8	15680 ± 70	19060 - 18720
Beta-359938	CC12	1 (not recovered)	0.1095 ± 0.0010			-19.1	17770 ± 70	21760 - 21170
Beta-359937	CC09	1 (not recovered)	0.0953 ± 0.0008			-24.8	18880 ± 70	22930 - 22470
Beta-359936	CC06	1 (not recovered)	0.1072 ± 0.0009			-22.1	17940 ± 70	21910 - 21440
Beta-359935	CC01	1 (not recovered)	0.0691 ± 0.0009			-23.1	21460 ± 100	25950 - 25520
Charcoal								
S-ANU 52436	SW9-16-CO2	4	0.0041 ± 0.0006	60	23.2		44115 ± 1224	49880 – 45590*
S-ANU 52437	SW9-16-04	Multiple	0.0075 ± 0.0006	59	12.6		39266 ± 674	44340 - 42170
S-ANU 40224	SW9-13-42a	1	0.51 ± 0.05	33	1.68		42350 ± 900	47840 - 44140

Supplementary Note 7: U-series direct dating of South Walker Creek megafauna fossils.

Gilbert J. Price

Fossil teeth are open systems for U. Living tissues contain little to no U, thus for such fossil materials to be dateable with U-series methods, U must be taken up from the burial environment post-deposition. U subsequently decays via alpha and beta emissions via a series of intermediate, short-lived isotopes, principally ^{234}U, ^{234}Th, ^{230}Th, and ^{231}Pa. The U-Th age is then calculated by determining the amount of ^{230}Th in relation to the original ^{238}U (via ^{234}U). This U-Th age will typically be a minimum, or 'fossilisation', age in the most ideal circumstances. The degree to which a given specimen will approximate a 'true' age can, in part, be determined by using other independent geochronological methods. In some situations, U, which is highly soluble in water, may be leached from a tooth at various times during or after radioactive decay, thus resulting in age overestimation (in contrast, Th is typically insoluble in water). Such U leaching, and thus the reliability of a given fossil tooth to produce meaningful geochronological information, can be determined by constructing ^{230}Th age and U profiles through a given tooth.

In this study, we dated several fossil teeth with U-Th methods using two sampling approaches: micro-profiling (described in this section) and laser ablation (see below). For micro-profiling, we followed the approach described in Price[116] whereby samples of dentine were collected from transects through a given tooth using 1 mm diameter stainless steel drill bits (Supplementary Figures 27-30). In most cases, the dentine was already exposed as a result of mastication-related tooth wear or post-mortem breakages of the respective teeth. One specimen, QMF54689 (molar from a *Diprotodon optatum* lower jaw), was sampled by drilling a single hole into the occlusal surface of the dentine, with powders collected at approximate intervals of 1 mm for the entire depth of the hole (ca. 10 mm). Although laser ablation profiling requires substantially less material for dating (see below), this approach is less destructive in that the fossil did not need to be cut and slabbed, thus remains largely intact, barring the small drill hole (Supplementary Figure 25).

24 independent ages were generated from transects through four teeth (Supplementary Figures 27-30; Supplementary Table 20). In each tooth, U concentration varied somewhat, but we believe that this is more a consequence of challenges in accurately weighing very small masses (i.e., individual dentine samples weighed, on average, 2 mg.). In contrast, the ^{230}Th age profiles through the respective teeth are remarkably consistent resulting in 'plateau' age profiles in each dated specimen. Thus, we find no evidence of post-burial / post-fossilisation loss of U from the teeth, strongly

indicating that the teeth have provided reliable minimum ^{230}Th ages (Supplementary Table 20). In fact, it appears that the teeth, although open systems for U, may have acted as closed-systems following recrystallisation of the phosphates.

The minimum ^{230}Th ages of the four micro-profiled teeth of *Pallimnarchus* sp. (QMF59869), *D. optatum* (QMF54689), *Sedophascolomys* sp. cf. *S. medius* (QMF57069), and *Phascolonus gigas* (QMF57065) are ca. 20 ka, 15 ka, 23 ka, and 23 ka, respectively (Supplementary Table 20).

Supplementary Table 20. U-series results for micro-profiled teeth and measured in the Radiogenic Isotope Facility at The University of Queensland**.**

**Taxon QMF**	**Sample Name**	**U (ppm)**	**^{232}Th (ppb)**	**(^{230}Th/ ^{232}Th)**	**(^{230}Th/238U)**	**(^{234}U/ ^{238}U)**	**Uncorr. Age (ka)**	**corr. Age (ka)**	**corr. Initial (^{234}U/ ^{238}U)**
Pallimnarchus sp. QMF59869	SW-1A	166.12 ± 0.11	20.1 ± 0.2	5958	0.238 ± 0.001	1.425 ± 0.001	19.8 ± 0.1	19.8 ± 0.1	1.449 ± 0.001
	SW-1B	70.46± 0.03	2.9 ± 0.1	15347	0.210 ± 0.002	1.389 ± 0.002	17.8 ± 0.1	17.8 ± 0.1	1.409 ± 0.002
	SW-1C	147.54 ± 0.09	19.5 ± 0.2	5740	0.250 ± 0.001	1.420 ± 0.001	21.0 ± 0.1	21.0 ± 0.1	1.446 ± 0.001
	SW-1D	131.81 ± 0.08	11.8 ± 0.1	7949	0.234 ± 0.001	1.419 ± 0.001	19.5 ± 0.1	19.5 ± 0.1	1.443 ± 0.001
	SW-1E	79.12 ± 0.04	17.7 ± 0.1	3382	0.249 ± 0.001	1.418 ± 0.001	20.9 ± 0.1	20.9 ± 0.1	1.443 ± 0.002
Diprotodon optatum QMF54689	SW9-31-A	131.56 ± 0.07	25.9 ± 0.3	3284	0.213 ± 0.001	1.462 ± 0.001	17.1 ± 0.1	17.1 ± 0.1	1.485 ± 0.001
	SW9-31-B	104.55 ± 0.04	69.3 ± 0.2	1030	0.225 ± 0.001	1.467 ± 0.001	18.0 ± 0.1	18.0 ± 0.1	1.491 ± 0.001
	SW9-31-C	112.57 ± 0.07	22.5 ± 0.2	3070	0.202 ± 0.001	1.464 ± 0.001	16.1 ± 0.1	16.1 ± 0.1	1.486 ± 0.001
	SW9-31-D	100.68 ± 0.05	23.2 ± 0.2	2586	0.196 ± 0.001	1.463 ± 0.001	15.6 ± 0.1	15.6 ± 0.1	1.484 ± 0.001
	SW9-31-E	121.38 ± 0.06	17.5 ± 0.2	4016	0.190 ± 0.001	1.460 ± 0.001	15.1 ± 0.1	15.1 ± 0.1	1.480 ± 0.001
	SW9-31-F	117.47 ± 0.07	8.7 ± 0.2	7931	0.194 ± 0.001	1.459 ± 0.002	15.5 ± 0.1	15.5 ± 0.1	1.480 ± 0.002
	SW9-31-G	118.24 ± 0.06	19.7 ± 0.3	3612	0.199 ± 0.001	1.459 ± 0.001	15.9 ± 0.1	15.9 ± 0.1	1.480 ± 0.001
	SW9-31-H	105.92 ± 0.07	41.4 ± 0.3	1562	0.201 ± 0.001	1.460 ± 0.001	16.1 ± 0.1	16.1 ± 0.1	1.481 ± 0.001
	SW9-31-I	96.44 ± 0.04	15.8 ± 0.2	3724	0.202 ± 0.002	1.460 ± 0.001	16.1 ± 0.1	16.1 ± 0.1	1.481 ± 0.001
	SW9-31-J	107.46 ± 0.07	4.4 ± 0.1	14853	0.201 ± 0.001	1.461 ± 0.001	16.1 ± 0.1	16.1 ± 0.1	1.483 ± 0.001
Sedophascolomys sp. cf. *S. medius* QMF57069	SW9-66-A	149.80 ± 0.08	35.4 ± 0.2	3598	0.281 ± 0.001	1.457 ± 0.001	23.1 ± 0.1	23.1 ± 0.1	1.488 ± 0.002
	SW9-66-B	185.13 ± 0.10	9.8 ± 0.4	15816	0.276 ± 0.001	1.453 ± 0.002	22.7 ± 0.1	22.7 ± 0.1	1.484 ± 0.002
	SW9-66-C	195.43 ± 0.10	10.8 ± 0.3	15384	0.280 ± 0.001	1.455 ± 0.002	23.1 ± 0.1	23.1 ± 0.1	1.486 ± 0.002
	SW9-66-D	182.95 ± 0.07	81.2 ± 0.8	1886	0.276 ± 0.002	1.464 ± 0.002	22.6 ± 0.1	22.6 ± 0.1	1.494 ± 0.002
	SW9-66-E	182.05 ± 0.09	457.8 ± 1.8	331	0.274 ± 0.001	1.468 ± 0.001	22.3 ± 0.1	22.3 ± 0.1	1.498 ± 0.001
Phascolonus gigas QMF57065	SW9-13-A	197.25 ± 0.08	929.3 ± 232.0	183	0.284 ± 0.001	1.449 ± 0.001	23.6 ± 0.1	23.5 ± 0.1	1.481 ± 0.002
	SW9-13-B	174.23 ± 0.08	241.8 ± 8.6	612	0.280 ± 0.001	1.452 ± 0.002	23.1 ± 0.1	23.1 ± 0.1	1.482 ± 0.002
	SW9-13-C	210.60 ± 0.12	269.6 ± 94.6	700	0.296 ± 0.001	1.453 ± 0.001	24.5 ± 0.1	24.5 ± 0.1	1.486 ± 0.001
	SW9-13-D	158.86 ± 0.09	109.6 ± 44.0	1233	0.280 ± 0.001	1.454 ± 0.001	23.1 ± 0.1	23.1 ± 0.1	1.485 ± 0.001

Note: Ratios in parentheses are activity ratios calculated from the atomic ratios, but normalised to measured values of secular-equilibrium HU-1 standard following the method of Ludwig[117]. Errors are at 2σ level. ^{230}Th ages are calculated using Isoplot EX 3.0 (Ludwig[118] with decay constants $\lambda_{238} = 1.551 \times 10^{-10}$ yr^{-1} (for ^{238}U), $\lambda_{234} = 2.826 \times 10^{-6}$ yr^{-1}(for ^{234}U) and $\lambda_{230} = 9.158 \times 10^{-6}$ yr^{-1} (for ^{230}Th), respectively (after Cheng[119]). 2σ errors in the uncorrected (uncorr.) ages were propagated directly from the uncertainties in the (^{230}Th/^{238}U) and (^{234}U/^{238}U). The corrected (corr.) ^{230}Th age was calculated using the assumed bulk earth or upper crust value equivalent to the detrital ^{230}Th/^{232}Th activity ratio of 0.83.

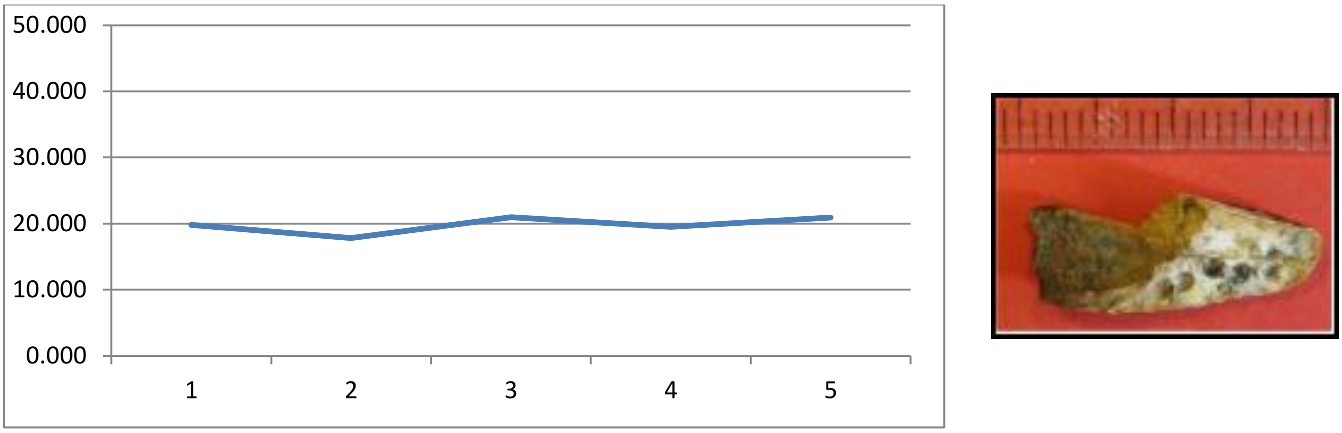

Supplementary Figure 27. SW-1A-E *Pallimnarchus* sp. tooth QMF59869, QML1470 (SW9)

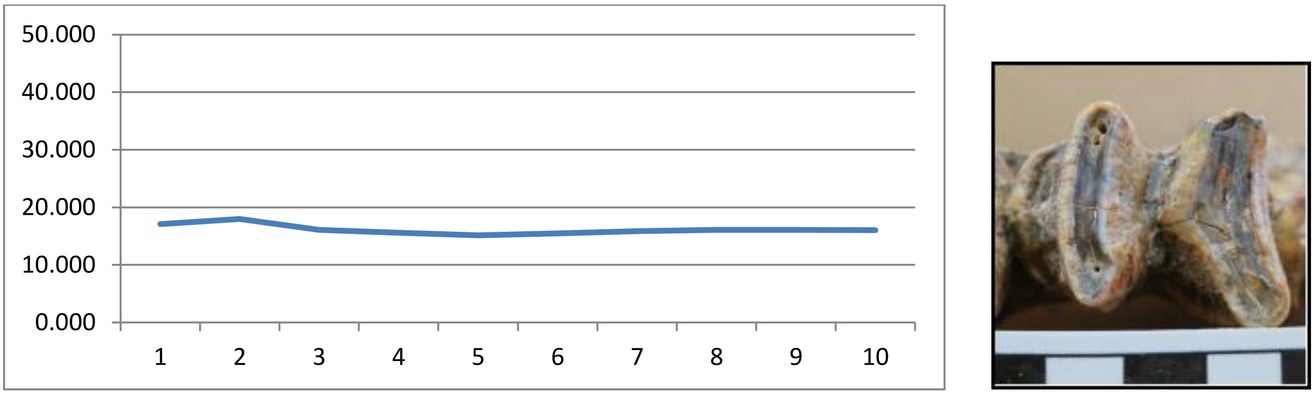

Supplementary Figure 28. SW9-31- *Diprotodon optatum* QMF54689 M_4 (associated mandible), QML1470 (SW9)

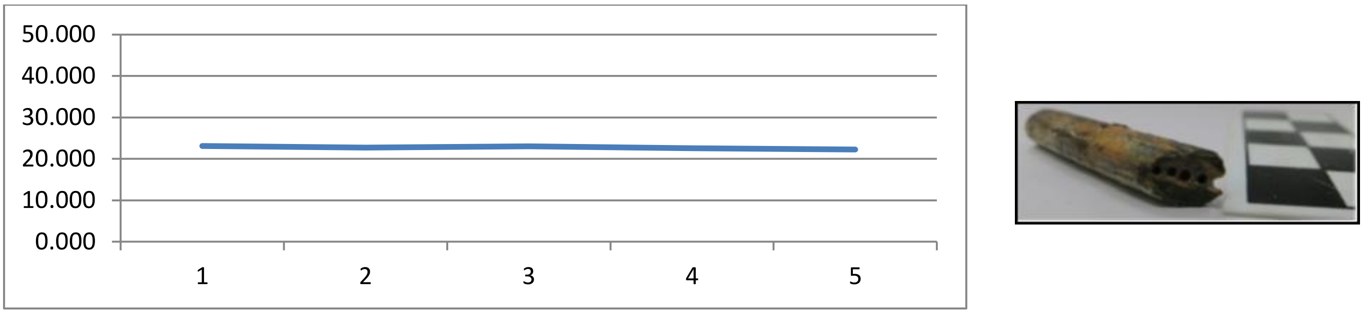

Supplementary Figure 29. SW9-66 *Sedophascolomys* sp. cf. *S. medius* (associated incisors) QMF57069, QML1470 (SW9)

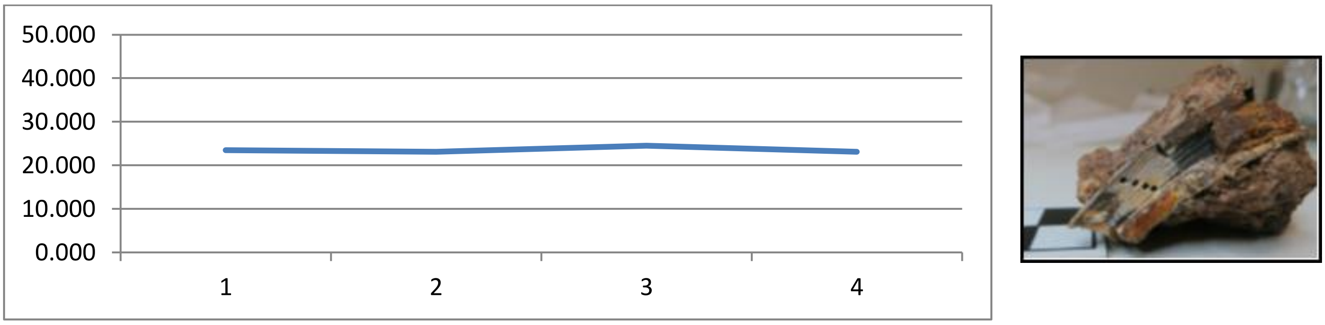

Supplementary Figure 30. SW13 – *Phascolonus gigas* molar (associated remains) QMF57065, QML1470 (SW9)

Supplementary Note 8: U-series laser ablation and ESR direct dating of South Walker Creek megafauna fossils.

Renaud Joannes-Boyau & Anthony Dosseto

Sample description and methods

Samples used for the dating showed signs of some level of diagenetic patterns, with superficial discoloration of the enamel and traces of sediment introduction in the dentine. The sample set consisted of (i) (SW9-MSCUA and SW9-MSCUB) a *Phascolonus gigas* molar tooth fragment removed from a complete molar of associated skeletal remains of individual QMF57065. US-ESR and U-Th dating (UQ) was applied to this specimen. The molar was still embedded in matrix; (ii) (SW9-PSCU-01) a complete *Protemnodon* sp. nov? (QMF57035) right P^3 from an associated adult individual with shinny enamel and white dentine; (iii) (SW3-SCU-01) a *Diprotodon optatum* molar tooth fragment from skull (QMF57172) with several discoloration and sediment introductions; and (iv) (SW9-ISCU-02) a near complete *Macropus* sp. (large) incisor, slight discoloration of the enamel and sediment introduction into the dentine (QMF57039). (i) The SW9-MSCUA and SW9-MSCUB are two fragments from the same *Phascolonus gigas* molar. The tooth was sectioned in half exposing dentine and parts of enamel. Directly next to the Uranium micro-drilling, a fragment of 8mm length was cut from the tooth containing both dentine and enamel. Two enamel fragments A and B were extracted from the fossil.

SW9-PSCU-01 was sectioned at one of the extremity of the tooth, and a small piece of enamel including dentine was removed. The fragment was profiled by LA-ICPMS for DAD U-series modelling and a wide fragment directly in contact was removed, divided in two (A&B), and cleaned for ESR experiments. (see detailed protocol below). SW3-SCU-01 was sectioned and preliminary measurements showed that the tooth was not suitable for US-ESR dating. SW9-ISCU-02 was sectioned at the base along the growth axis. A small fragment was removed and analysed DAD U-series modelling, an enamel fragment directly attached was removed and cleaned for ESR experiments (see detailed protocol below). Two enamel fragments (A&B) were extracted for US-ESR dating. Unfortunately, one fragment (A) broke during experiments and could not give an ESR age.

In-situ U-series analysis

Open-system uranium-series series dating of the teeth was undertaken by laser ablation multi-collector ICP-MS at the Wollongong Isotope Geochronology Laboratory, University of Wollongong.

Laser ablation was performed with a New Wave Research 193 nm ArF excimer laser, equipped with a TV2 cell. Thorium (^{230}Th, ^{232}Th) and uranium (^{234}U, ^{235}U, ^{238}U) isotopes were measured on a Thermo Neptune Plus multi-collector ICP-MS mounted with jet sample and x-skimmer cones. All five isotopes were collected in static mode, with ^{230}Th and ^{234}U collected in ion counters. Helium flow rate and ICP-MS parameters were tuned with NIST612 element standard to derive a ^{232}Th/^{238}U ratio for this standard greater than 0.8 and thus minimise differences in fractionation between Th and U[120]. For tuning, a fluence of 2.3 J/cm2, pulse rate of 20 Hz, spot size of 100 µm and scan speed of 5 µm/s was used. This yielded 0.35V of ^{238}U and 0.32V of ^{232}Th. Uranium-238 tail on ^{234}U, and ^{232}Th tail on ^{230}Th (although negligible for phosphates and carbonates) were measured using a coral and glass standard NIST612, respectively. A fluence of 0.9 J/cm2, pulse rate of 10 Hz, spot size of 150 µm and scan speed of 5 µm/s was used. The cut teeth were ablated using rasters instead of spots. Trials performed on a MIS5 coral (unpublished. data) show that while calculated ages were within error of each other between raster and spot analyses, counting statistics was better using raster analyses (because ablation duration was ~120 sec, versus 60 sec for spot analysis). Each raster was ~620 µm long. A fluence of 0.9 J/cm2, pulse rate of 10 Hz, spot size of 150 µm and scan speed of 5 µm/s was used. A trial was done using spot analyses instead (same pulse rate, fluence and spot size; ablation duration = 60 sec). A longer ablation duration for spot analysis is not recommended since in this case downhole fractionation becomes significant. Helium was used as a carrier gas at a flow rate of 0.9 L/min. Before and after each sample, three rasters were done on NIST612, MK10 (a MIS7 coral used as primary standard[121]; and MK16 (a MIS5 coral used as secondary standard[121];. Measured ^{234}U/^{238}U and ^{230}Th/^{238}U isotopic ratios were corrected for elemental fractionation and Faraday cup/SEM yield by comparison with MK10 coral (see above) for which ratios were previously characterised internally by solution analysis. Concentrations of U and Th were determined using NIST612 glass as calibration standard. Background subtraction, concentration quantification and ratio corrections were performed using Iolite™ software. The corrected (^{234}U/^{238}U) and (^{230}Th/^{238}U) isotope ratios for the secondary standard (MK16 coral; 1.106 ± 0.003 and 0.759 ± 0.06, respectively; 2σ, n=15) were within error of the values determined by solution analysis (1.110 ± 0.002 and 0.764± 0.007). The calculated closed-system ^{230}Th-U age for MK16 was 123 ± 2 ka (2σ, n=15), within error of the value determined by solution analysis (124 ± 2 ka).

Modelling of open-system U-series age

In-situ U-series analyses were used to calculate an open-system age for each tooth. On QML1470 incisor I-SCU-02, 10 rasters were produced, parallel to the surface (Supplementary Figure 30). On QML1470 SW9-2015 P-SCU01, 20 rasters were produced, parallel to the surface (Supplementary

Figure 31). The Diffusion-Adsorption-Decay model of Sambridge[122] was used to derive an open-system U-series age from each transect of analyses. This was undertaken using *UThwigl* R package[123] which is available upon request from A.D. The uranium diffusion coefficient was allowed to take values between 10^{-11} and 10^{-14} cm^2/s.

U-series for ESR model

For the dentine directly in contact with the enamel fragments used for ESR dating, several rasters were measured and averaged to obtain the U-series values. For the ESR fragments SW9-ISCU-02B, SW9-PSCU-01A and SW9-PSCU-01B the concentration was obtained using LA-ICPMS quadrupole Agilent 7700 (large cell 20Hz, ablation raster of 100µm spot size, 1min ablation length, 5µm ablation depth, translation speed 20µm.s-1, NIST 612 correction for drift and tooth standard with known concentration for matrix effect), while the remaining enamel attached to the dentine was used to obtained the U-series values. All age calculations were carried out with the USESR program[124], which utilizes the dose rate conversion factors[90].

ESR protocol

ESR dating was performed on a Freiberg MS5000 X-band at 1G modulation amplitude, 2mW power, 100G sweep, 100KHz modulation frequency. X-ray irradiation was performed at SCU on a Freiberg X-ray irradiation chamber which contains a Varian VF50, with irradiation parameters: 40kV voltage and 0.5mA current with dose rate calibrating depending on the output value of the X-ray gun. Each ESR fragment was irradiated with an incremental dose step at 90s, 380s, 900s, 1800s, 3600, 7200, 14400s, with an average dose rate of 0.25Gy.s-1. For each irradiation step, the energy output of the X-ray gun is recorded at the beginning and end and averaged, which allows us to correct for the dose rate received by the sample. For each irradiation step the fragment was measured over 180o in x, y and z-configurations with a 20° step[125, 126] . ESR intensities were extracted from T1-B2 peak-to-peak amplitudes on the merged ESR signal. Isotropic and baseline corrections were applied uniformly across the measured spectra[127]. The amount of NOCORs was estimated using the protocol described by Joannes-Boyau[126]. The sample was mounted onto a Teflon sample holder, which expose directly the fragment to the x-ray source with no shielding (apart from a 200-micron aluminium foil). Dose response curve (DRC) and equivalent dose (De) were calculated using the MCDoseE 2.0 program[128]. The program uses a Bayesian framework, where the solution is a full probability distribution on the dose equivalent (for more information see[128, 129]). DRC was estimated using SSE the maximum irradiation dose (Dmax) adjusted to meet the criteria defined[130] (Supplementary Figure 33). The external dose was calculated using the values obtained by the gamma spectrometer measurement in

the field. The U, Th and K concentration in the sediment surrounding the tooth was obtain from in-situ measurements. Cosmic dose rate was estimated from Prescott [131].

RESULTS

Open-system U-series ages

For QML1470 incisor I-SCU-02 (Supplementary Figure 30), the calculated age is 25.5 ± 0.1 ka. The calculated initial ($^{234}U/^{238}U$) ratio at x = -1 is 1.46 and the diffusion coefficient is 9.09x10^{-12} cm^2/s. For QML1470 SW9-2015 P-SCU01 (Supplementary Figure 31), only the 11 rasters in the dentine were used, considering the very low U concentrations in the enamel. The calculated age is 22.6 +0.6/-0.5 ka. The calculated initial ($^{234}U/^{238}U$) ratio at x = -1 is 1.40 and the diffusion coefficient is 5.98x10^{-12} cm^2/s.

Supplementary Table 21. Uranium-series isotope data for the teeth and the coral standard (MK16) produced at University of Wollongong

Raster ID	Position relative to the center	Th (ppm)	2σ	U (ppm)	2σ	($^{234}U/^{238}U$)	2σ	($^{230}Th/^{238}U$)	2σ	($^{232}Th/^{238}U$)	2σ
I_SLU_02_0	-0.49	0.0172	0.0048	351	5.1	1.4641	0.0015	0.2928	0.0018	1.79E-05	5.2E-06
I_SLU_02_1	-0.34	0.011	0.0047	245.9	6.4	1.4675	0.0021	0.294	0.0023	1.42E-05	0.000006
I_SLU_02_2	-0.18	0.0092	0.0039	239	6.5	1.4656	0.0015	0.3004	0.0026	1.26E-05	5.5E-06
I_SLU_02_3	-0.03	0.0139	0.0099	240.3	3.7	1.4671	0.0019	0.3026	0.0022	0.000019	0.000014
I_SLU_02_4	0.12	0.0184	0.009	241.7	6.7	1.4633	0.0017	0.3053	0.0019	0.000027	0.000014
I_SLU_02_5	0.28	0.035	0.03	239.4	5.3	1.4623	0.0018	0.3053	0.0022	0.000052	0.000049
I_SLU_02_6	0.43	0.005	0.0017	250.4	6.5	1.4597	0.0024	0.3094	0.0024	6.5E-06	2.2E-06
I_SLU_02_7	0.58	0.00095	0.00027	264.2	3.2	1.4577	0.0025	0.3151	0.0022	1.18E-06	3.3E-07
I_SLU_02_8	0.74	0.00084	0.00021	262.9	2.7	1.4571	0.0023	0.3154	0.002	1.04E-06	2.5E-07
I_SLU_02_9	0.89	0.00113	0.00054	231	5.2	1.4578	0.0023	0.3169	0.0024	1.65E-06	8.4E-07
P_SLU_01_0		*0.0009*	*0.0013*	*0.33*	*0.25*	*1.343*	*0.064*	*0.49*	*0.17*	*0.00025*	*0.00094*
P_SLU_01_1		*0.0002*	*0.00027*	*0.25*	*0.14*	*1.45*	*0.1*	*0.5*	*0.24*	*0.00015*	*0.00077*
P_SLU_01_2		*0.002*	*0.0034*	*0.199*	*0.046*	*2.5*	*1.1*	*3.7*	*3.2*	*0.0007*	*0.0011*
P_SLU_01_3		*Below LOD*	*Below LOD*	*0.313*	*0.044*	*1.424*	*0.062*	*0.256*	*0.052*	*-0.00003*	*0.00019*
P_SLU_01_4		*Below LOD*	*Below LOD*	*3.19*	*0.62*	*1.389*	*0.02*	*0.21*	*0.014*	*-1.5E-05*	*0.00002*
P_SLU_01_5		*0.00037*	*0.00077*	*4.63*	*0.56*	*1.392*	*0.01*	*0.2255*	*0.0074*	*0.000049*	*0.000097*
P_SLU_01_6		*0.00006*	*0.00013*	*3.54*	*0.27*	*1.411*	*0.019*	*0.262*	*0.015*	*0.000004*	*0.000013*
P_SLU_01_7		*0.00033*	*0.0005*	*4.14*	*0.12*	*1.41*	*0.014*	*0.252*	*0.01*	*0.000025*	*0.00004*
P_SLU_01_8		*0.00009*	*0.00014*	*189.6*	*8.3*	*1.3995*	*0.0022*	*0.2365*	*0.002*	*1.7E-07*	*2.4E-07*
P_SLU_01_9	-0.91	0.00016	0.00015	258.3	9.3	1.4021	0.0019	0.2671	0.0023	1.8E-07	1.9E-07
P_SLU_01_10	-0.73	0.0002	0.00022	253.1	9.8	1.4006	0.0024	0.2605	0.002	2.5E-07	2.7E-07
P_SLU_01_11	-0.55	0.00027	0.00029	252	13	1.3992	0.0026	0.2522	0.0026	3.2E-07	4.1E-07
P_SLU_01_12	-0.37	0.00004	0.00022	180	34	1.4003	0.0044	0.255	0.003	-2E-07	5.7E-07
P_SLU_01_13	-0.19	Below LOD	Below LOD	109	35	1.4051	0.0054	0.274	0.0063	-2E-06	1.5E-06
P_SLU_01_14	-0.01	Below	Below	58	21	1.411	0.0079	0.283	0.02	-3.7E-06	2.5E-06

		LOD	LOD								
P_SLU_01_15	0.17	Below LOD	Below LOD	36.2	7.7	1.4054	0.0066	0.276	0.01	-2.9E-06	2.5E-06
P_SLU_01_16	0.35	0.00002	0.00037	39.8	3.5	1.4106	0.0068	0.2749	0.0064	-2E-06	0.000003
P_SLU_01_17	0.53	0.00027	0.00026	21.3	1.3	1.41	0.0083	0.2566	0.0055	3.7E-06	3.8E-06
P_SLU_01_18	0.71	0.00016	0.00019	13	1.2	1.409	0.011	0.2678	0.007	3.9E-06	5.2E-06
P_SLU_01_19	0.89	Below LOD	Below LOD	10.24	0.97	1.4112	0.0098	0.271	0.014	-3.1E-06	6.6E-06
MK16 (n=15)		0.00021	0.00006	3.3	0.1	1.106	0.003	0.759	0.006	2.3E-05	7E-06

Activity ratios are corrected values, using a MIS7 coral as standard (for which isotopic ratios were characterised by solution analysis). Parentheses denote activity ratios. Italicised rows are data not used for open-system model age calculation, because of large errors on either activity ratio. Position relative to the center = -1 or 1 for either surface of the sample, 0 for the center of the transect.

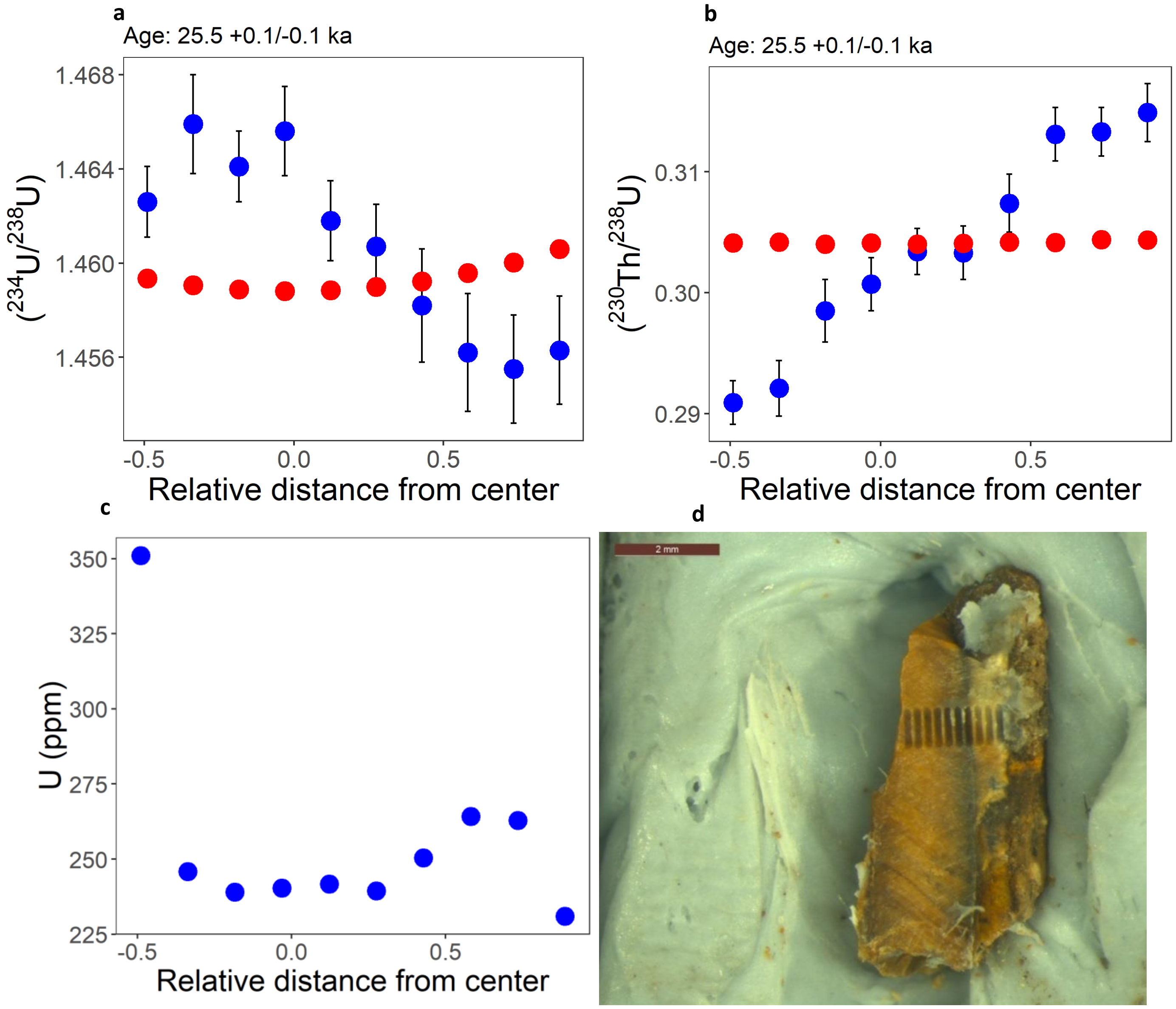

Supplementary Figure 31. Measured (a) (234U/238U) and (b) (230Th/238U) activity ratios (blue dots) and modelled ratios (red dots) for QML1470 incisor I-SCU-02. (c) Measured uranium concentrations. The x axis represents dimensionless positions, where x=-1 and 1 are surfaces on either side of the sample. Decreasing the diffusion coefficient would result in a higher curvature in the modelled compositions, however it does not significantly affect the calculated age. (d) Picture of the sample (QMF57039) after ablation**.**

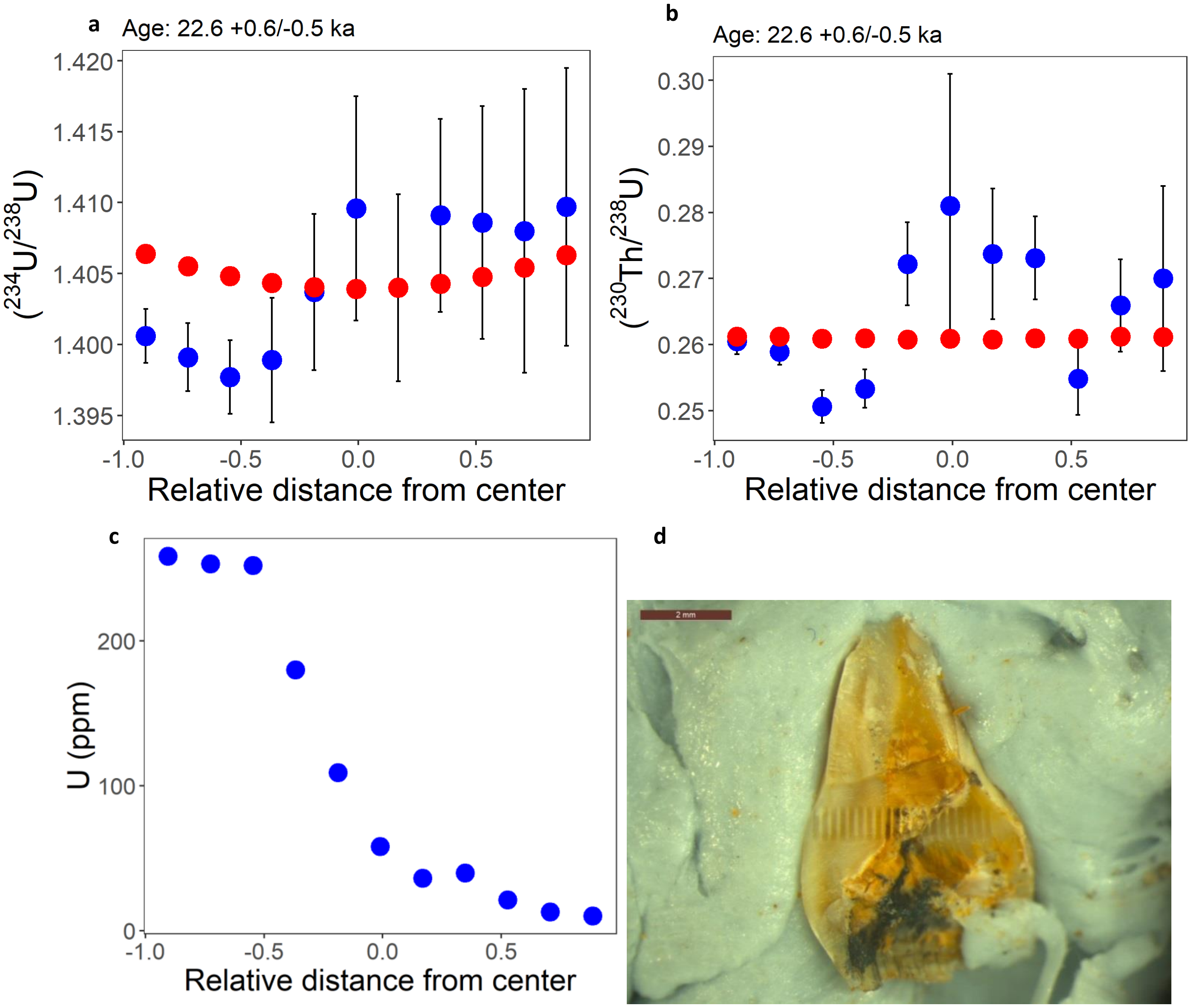

Supplementary Figure 32. Measured (a) (234U/238U) and (b) (230Th/238U) activity ratios (blue dots) and modelled ratios (red dots) for QML1470 SW9-2015 P-SCU01 dentine. (c) Measured uranium concentrations. The x axis represents dimensionless positions, where x=-1 is the boundary between the enamel and the dentine, and x=1 is the base of the dentine. (d) Picture of the sample (QMF57035) after ablation. Note the nine tracks on the left (in the enamel) were not used for open-system age modelling as the errors on activity ratios were too large.

US-ESR age modelling

The detailed calculation, values and aliquots ages of South Walker Creek fossil teeth for SW9-MSCU, SW9-PSCU-01 and SW9-ISCU-02 are summarized in Supplementary Table 22, with ages of 35±11ka, 41±11ka and 30±7ka respectively. Two teeth were dated using two aliquots. SW9-MSCU A and B and PSCU A and B yield ages consistent with each other and statistically indistinguishable from each other respectively. Unexpectedly, the large concentration of uranium within the enamel of MSCU A and B does not seem to be detrimental to the age calculation. Perhaps, the destruction of radical by alpha particles has not been sufficient to impact the DRC yet. It has to be noted that the DRC of SW9-PSCU-01B presented outsider points that influenced the accuracy of the fitting, hence the large error on the equivalent dose. The minor offset in age between the SW9-PSCU-01B and

SW9-PSCU-01A replicates could be attributed to the potential U-series differences within each enamel fragment, for which the values were not measured. However, the replicate results remain within error of each other, and are therefore both considered in the final age evaluation of SW9.

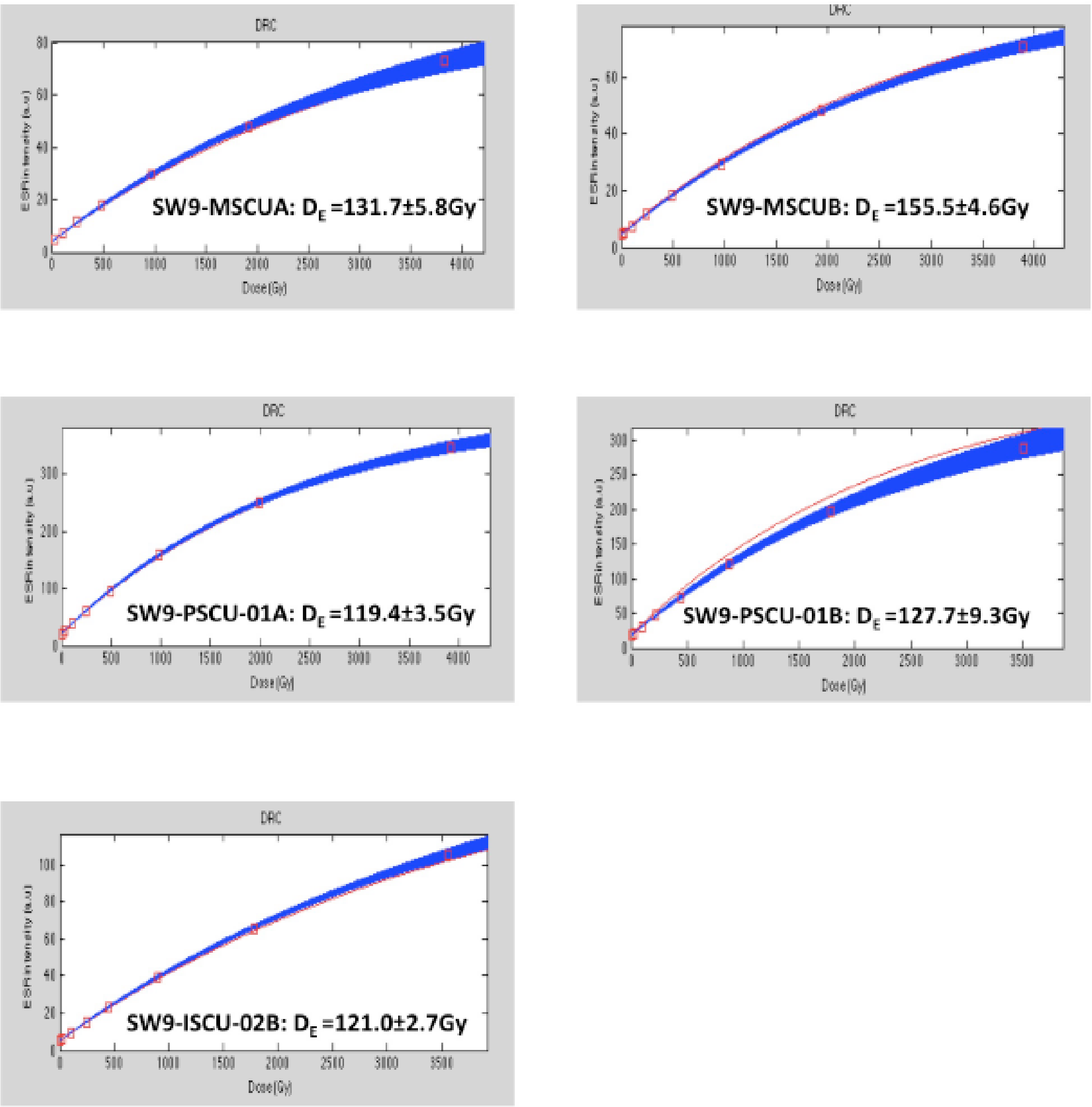

Supplementary Figure 33. Dose Response curve (DRC) were calculated using MCDoseE 2.0, with 250k iterations and Burning of 125k and a Double Saturated Exponential (DSE) function (Joannes-Boyau et al., 2018). D_E values are express at 2-sigma error. D_E were used to calculate the US-ESR age reported in Supplementary Table 22.

Supplementary Table 22. US-ESR dating data for SW9 teeth. a Dose equivalent De obtained using McDoseE 2.0, with DSE [128].

**SAMPLE SW9**	**MSCU-1(A)**	**MSCU-1(B)**	**PSCU-01A**	**PSCU-01B**	**ISCU-02B**
	**ENAMEL**				
Dose (Gy)[a]	131.7±5.8	155.5±4.6	119.4±3.5	127.7±9.3	121.0±2.7
U (ppm)	10.65±0.94	17.2±1.3	6.78±0.38		7.01±0.8
234U/238U	1.4150±0.028	1.4035±0.074	1.3990±0.0160		1.4550±0.0223
230Th/234U	0.1910±0.031	0.1904±0.046	0.1680±0.0181		0.1981±0.077
Thickness (m)	862±172	805±161	743±149	758±152	881±106
Water (%)	3±1				
	**DENTINE**				
U (ppm) [b]	187.8±5.1	207.5±5.4	220.8±14.5		250.4±5
234U/238U	1.4831±0.060		1.3988±0.0027		1.4607±0.0021
230Th/234U	0.1890±0.0149		0.1806±0.0096		0.2080±0.0136
Water (%)	5±3				
	**SEDIMENT**				
U (ppm)	2.01±0.12				
Th (ppm)	10.03±0.48				
K (%)	1.30±0.06				
Water (%)	22±4				
	**EXTERNAL DOSE RATE SEDIMENT**				
Beta Dose (μGy a^{-1})	162±26	173±27	186±28	182±27	158±17
G&C Dose (μGy a^{-1})	983±79				
	**COMBINE US-ESR AGE**				
Internal dose rate (μGy a^{-1})	1220±548	2225±891	637±285	568±301	1007±444
Beta Dose rate dentine (μGy a^{-1})	1104±498	1478±592	1336±598	1169±619	1885±830
P enamel	-0.39±0.32	-0.62±0.28	-0.15±0.37	0.13±0.46	-0.76±0.27
P dentine	-0.37±0.32	-0.61±0.28	-0.29±0.34	-0.03±0.43	-0.82±0.25
Total Dose rate (μGy a^{-1})	3466±745	4859±1073	3142±668	2902±693	4033±945
**AGE (ka)**	**38±8**	**32±7**	**38±8**	**44±10**	**30±7**

Supplementary Note 9: Palynological Assessment of QML1470 (SW9)

Patrick Moss

Relatively low pollen yields (96 grains and spores) were recovered from the QML1470 (SW9) sub-samples, which have been combined into a single pollen and charcoal record (Supplementary Figure 33 and Supplementary Table 23). This may reflect an environment that underwent a high level of oxidation, as pollen preservation requires an anaerobic environment and may reflect climate variability, i.e. increased aridity at the time of deposition[132]. However, enough pollen is preserved to suggest that the regional environment was mainly a sclerophyll forest dominated by eucalypts and she oak (Casuarinaceae) canopy with a heath understorey. The streams would have been fringed by paperbark (*Melaleuca*) trees/shrubs and this landscape is typical of the region during Marine Isotope Stage 3 based on existing records from South East Queensland[133] and North East Queensland[134]. Fire was also an environmental component of the site based on the presence of charcoal particles in the sediment and *Sporormiella* was also found suggesting the presence of herbivourous grazers[135].

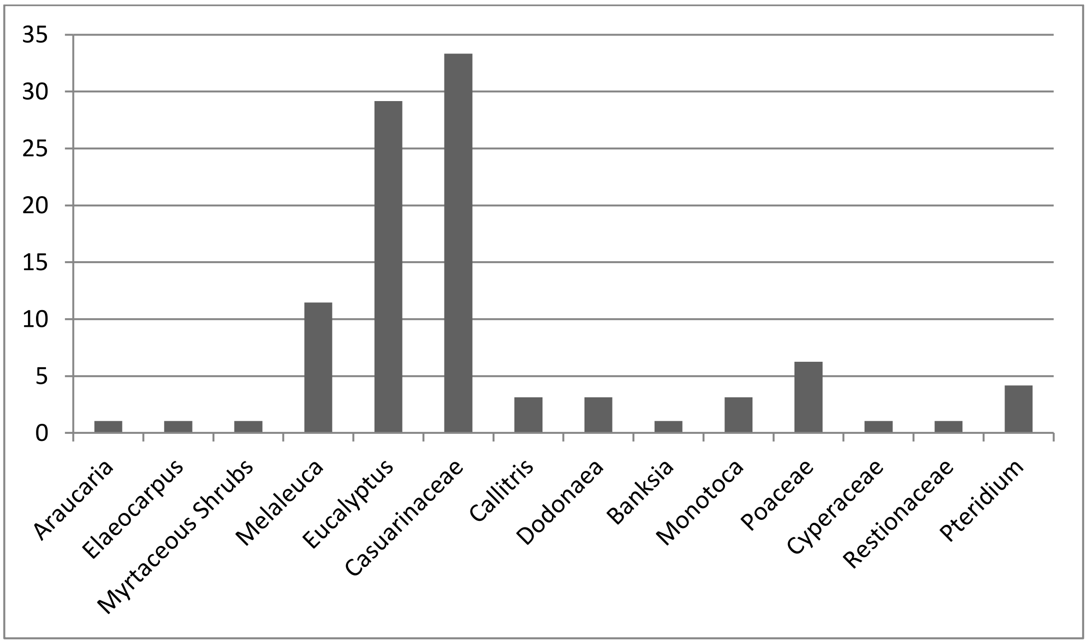

Supplementary Figure 34. Identified pollen counts from QML1470 (SW9).

Supplementary Table 23. Pollen, habitat interpretation, *Sporomiella* (dung fungus) and Charcoal counts for ten subsamples (SW1-SW10) from QML1470 (SW9).

	**HABITAT**	**SW 1**	**SW 2**	**SW 3**	**SW 4**	**SW 5**	**SW 6**	**SW 7**	**SW 8**	**SW 9**	**SW 10**	**Total**
Araucaria	Emergent Rainforest	0	0	1	0	0	0	0	0	0	0	1
Elaeocarpus	Canopy Rainforest	1	0	0	0	0	0	0	0	0	0	1
Myrtaceous Shrubs	Sclerophyll and Heath	0	0	1	0	0	0	0	0	0	0	1
Melaleuca	Wetland Canopy	0	0	1	0	4	4	0	1	0	1	11
Eucalyptus	Sclerophyll canopy	7	0	6	3	1	9	0	1	0	1	28
Casuarinaceae	Sclerophyll canopy	7	0	9	2	4	7	3	0	0	0	32
Callitris	Sclerophyll canopy	1	0	0	0	1	0	1	0	0	0	3
Dodonaea	Sclerophyll shrub (hopbush)	0	0	1	0	1	1	0	0	0	0	3
Banksia	Heath and Sclerophyll shrubs	0	0	0	0	0	0	1	0	0	0	1
Monotoca	Heath Shrub	1	0	2	0	0	0	0	0	0	0	3
Poaceae	Dryland and Aquatic Herb (grass)	3	0	2	0	0	0	0	0	0	1	6
Cyperaceae	Aquatic Herb (sedge)	0	0	0	0	0	1	0	0	0	0	1
Restionaceae	Aquatic Herb (rush)	0	0	0	0	0	1	0	0	0	0	1
Pteridium	Bracken	2	1	0	1	0	0	0	0	0	0	4
Total		22	1	23	6	11	23	5	2	0	3	96
Sporormiella concentration (grains/cm^3)		28.8	0	3.6	38.4	12.2	40.3	79.6	46.1	51.2	59.8	
Charcoal concentration (particles per cm^3)		4971	0	355	3585	5136	1428	7526	6514	695	515	

Supplementary References

1. Molnar R. *Pallimnarchus* and other Cenozoic crocodiles of Queensland. *Memoirs of the Queensland Museum* **20**, 657-673 (1982).

2. Molnar R. *Crocodylus porosus* from the Pliocene Allingham formation of North Queensland. Results of the Ray E. Lemley expeditions, part 5. *Memoirs of the Queensland Museum* **19**, 357-365 (1979).

3. Molnar R. Pleistocene ziphodont crocodilians of Queensland. *Records of the Australian Museum* **33**, 803-834 (1981).

4. Molnar RE. Pleistocene ziphodont crocodilians of Queensland. *Records of the Australian Museum* **33**, 803-834 (1981).

5. Willis P, Molnar R. A new middle Tertiary crocodile from Lake Palankarinna, South Australia. *Records of the South Australian Museum* **25**, 39-55 (1991).

6. Willis P, Molnar R. A review of the Plio-Pleistocene crocodilian genus *Pallimnarchus*. *Proceedings of the Linnean Society of New South Wales* **117**, 223-242 (1997).

7. Willis P, Molnar RE. Identification of large reptilian teeth from Plio-Pleistocene deposits of Australia. *Journal and Proceedings of the Royal Society of New South Wales* **130**, 79-92 (1997).

8. Chiotakis C. Pliocene crocodilians of Chinchilla: Identification using dental morphometrics. (ed^(eds). Queensland University of Technology (2018).

9. Sobbe IH, Price, G.J. & Knezour, R.A. . A ziphodont crocodile from the late Pleistocene King Creek catchment, Darling Downs, Queensland. *Queensland Memoirs of the Queensland Museum – Nature* **56**, 601–606 (2013).

10. Molnar R. Cenozoic fossil reptiles in Australia. *The Fossil Vertebrate Record of Australia'(Eds PV Rich and EM Thompson) pp*, 228-233 (1982).

11. Hocknull SA, Zhao J-X, Feng Y-X, Webb GE. Responses of Quaternary rainforest vertebrates to climate change in Australia. *Earth and Planetary Science Letters* **264**, 317-331 (2007).

12. Hocknull SA. Late Cainozoic rainforest vertebrates from Australopapua: evolution, biogeography and extinction. In: *Biological, Earth and Environmental Sciences, Faculty of Science* (ed^(eds). University of New South Wales (2009).

13. Hocknull SA. Ecological succession during the late Cainozoic of central eastern Queensland: extinction of a diverse rainforest community. *Memoirs of the Queensland Museum* **51**, 39-122 (2005).

14. Hocknull SA, Piper PJ, van den Bergh GD, Due RA, Morwood MJ, Kurniawan I. Dragon's Paradise Lost: Palaeobiogeography, Evolution and Extinction of the Largest-Ever Terrestrial Lizards (Varanidae). *PloS one* **4**, e7241 (2009).

15. Dawson L, Flannery T. Taxonomic and phylogenetic status of living and fossil kangaroos and wallabies of the genus *Macropus* Shaw (Macropodidae: Marsupialia), with a new subgeneric name for the larger wallabies. *Australian Journal of Zoology* **33**, 473-498 (1985).

16. Celik M*, et al.* A molecular and morphometric assessment of the systematics of the *Macropus* complex clarifies the tempo and mode of kangaroo evolution. *Zoological Journal of the Linnean Society*, (2019).

17. Bartholomai A. The genus *Macropus* Shaw (Marsupialia; Macropodidae) in the upper Cainozoic deposits of Queensland. *Memoirs of the Queensland Museum* **17**, 195-235 (1975).

18. De Vis CW. A review of the fossil jaws of the Macropodidae in the Queensland Museum. *Proceedings of the Linnean Society of New South Wales* **10**, 75-133 (1895).

19. Molnar R, Kurz C. The distribution of Pleistocene vertebrates on the eastern Darling Downs, based on the Queensland Museum collections. *Proceeds of the Linnean Society of New South Wales* **117**, 107-134 (1997).

20. Flannery TF, and Archer, M. . The taxonomy and distribution of *Macropus (Fissuridon) pearsoni* (Marsupialia: Macropodidae). *Australian Mammalogy* **5** 261-265 (1982).

21. Murray PF. The postcranial skeleton of the Miocene kangaroo, *Hadronomas puckridgi* Woodburne (Marsupialia, Macropodidae). *Alcheringa: An Australasian Journal of Palaeontology* **19**, 119-170 (2008).

22. Wells RT, Tedford RH. *Sthenurus* (Macropodidae, Marsupialia) from the Pleistocene of Lake Callabonna, South Australia. *Bullentin of the American Museum of Natural History* **225**, 1-112 (1995).

23. Flannery TF. Re-examination of the Quanbun Local Fauna, a late Cenozoic vertebrate fauna from Western Australia. *Records of the Western Australian Museum* **11**, 119-128 (1984).

24. Flannery TF. *Macropus mundjabus*, a new kangaroo (Marsupialia: Macropodidae) of uncertain age from Victoria, Australia. *Australian Mammalogy* **3**, 35-51 (1980).

25. Bartholomai A. *Fissuridon pearsoni*, a new fossil macropodid (Marsupialia) from Queensland. *Memoirs of the Queensland Museum* **16**, 365-368 (1973).

26. Bartholomai A. The genus *Protemnodon* Owen (Marsupialia, Macropodidae) in the upper Cainozoic deposits of Queensland. *Memoirs of the Queensland Museum* **16**, 309-363 (1973).

27. Bartholomai A. The type specimens of some of de Vis' species of fossil Macropodidae. *Memoirs of the Queensland Museum* **14**, 115-126 (1966).

28. Bartholomai A. The Macropodidae (Marsupialia) from the Allingham Formation, northern Queensland. Results of the Ray E. Lemley expeditions, Pt. 2. *Memoirs of the Queensland Museum* **18**, 127-143 (1978).

29. Flannery TF, Archer M. Revision of the genus *Troposodon* Bartholomai (Macropodidae: Marsupialia). *Alcheringa: An Australasian Journal of Palaeontology* **7**, 263-279 (2008).

30. Flannery TF. New Pleistocene marsupials (Macropodidae, Diprotodontidae) from subalpine habitats in Irian Jaya, Indonesia. *Alcheringa: An Australasian Journal of Palaeontology* **16**, 321-331 (1992).

31. Plane MD. *The stratigraphy and vertebrate fauna of the Otibanda Formation, New Guinea*. University of California (1965).

32. Piper KJ. The Macropodidae (Marsupialia) of the early Pleistocene Nelson Bay Local Fauna, Victoria, Australia. *Memoirs of Museum Victoria* **74**, (2016).

33. Dawson L. A new fossil genus of forest wallaby (Marsupialia, Macropodinae) and a review of *Protemnodon* from eastern Australia and New Guinea. *Alcheringa: An Australasian Journal of Palaeontology* **28**, 275-290 (2004).

34. Prideaux G. *Systematics and evolution of the sthenurine kangaroos*. University of California Press (2004).

35. Janis CM, Buttrill K, Figueirido B. Locomotion in extinct giant kangaroos: were sthenurines hop-less monsters? *PloS one* **9**, e109888 (2014).

36. Tedford RH. The fossil Macropodidae from Lake Menindee, New South Wales. . *University of California Publications in Geological Sciences* **64**, (1967).

37. Bartholomai A. The rostrum in *Palorchestes* Owen (Marsupialia: Diprotodontidae) Results of the Ray E. Lemley Expeditions, Part 3. *Memoirs of the Queensland Museum* **18**, 145-149 (1978).

38. Banks MR, Colhoun EA, Van De Geer G. Late Quaternary *Palorchestes azael* (Mammalia, Diprotodontidae) from northwestern Tasmania. *Alcheringa* **1**, 159-166 (1976).

39. Davis A, Archer M. *Palorchestes azael* (Mammalia, Palorchestidae) from the late Pleistocene Terrace Site Local Fauna, Riversleigh, northwestern Queensland. *Memoirs of the Queensland Museum* **41**, 315-320 (1997).

40. Mackness B. *Palorchestes selestiae*, a new species of palorchestid marsupial from the early Pliocene Bluff Downs Local Fauna, northeastern Queensland. *Memoirs of the Queensland Museum* **38**, 603-610 (1995).

41. Pledge NS. Occurrences of *Palorchestes* species (Marsupialia: Palorchestidae) in South Australia. *Records of the South Australian Museum* **25**, 161-174 (1991).

42. Price GJ, Hocknull S. A small adult *Palorchestes* (Marsupialia, Palorchestidae) from the Pleistocene of the Darling Downs, southeast Queensland. *Memoirs of the Queensland Museum* **51**, 202 (2005).

43. Trusler PW, Sharp AC. Description of new cranial material of *Propalorchestes* (Marsupialia: Palorchestidae) from the Middle Miocene Camfield Beds, Northern Territory, Australia. *Memoirs of Museum Victoria* **74**, 291-324 (2016).

44. Piper KJ. A new species of Palorchestidae (Marsupialia) from the Pliocene and early Pleistocene of Victoria. *Alcheringa: An Australasian Journal of Palaeontology* **30**, 281-294 (2006).

45. Black K. A new species of Palorchestidae (Marsupialia) from the late middle to early late Miocene Encore Local Fauna, Riversleigh, northwestern Queensland. *Memoirs of the Queensland Museum* **41**, 181-186 (1997).

46. Wells RT, Murray PF, Bourne SJ. Pedal morphology of the marsupial lion *Thylacoleo carnifex* (Diprotodontia: Thylacoleonidae) from the Pleistocene of Australia. *Journal of Vertebrate Paleontology* **29**, 1335-1340 (2009).

47. Wells R, Nichol B. On the manus and pes of *Thylacoleo carnifex* Owen (Marsupialia). *Transactions of the Royal Society of South Australia* **101**, 139-146 (1977).

48. Finch M, Freedman L. Functional morphology of the limbs of *Thylacoleo carnifex* Owen (Thylacoleonidae, Marsupialia). *Australian Journal of Zoology* **36**, 251-272 (1988).

49. Wells RT, Camens AB. New skeletal material sheds light on the palaeobiology of the Pleistocene marsupial carnivore, *Thylacoleo carnifex*. *PloS one* **13**, e0208020 (2018).

50. Dawson L. The status of the taxa of extinct giant wombats (Vombatidae: Marsupialia), and a consideration of vombatid phylogeny. *Australian Mammalogy* **4**, 65-79 (1981).

51. Louys J. Wombats (Vombatidae: Marsupialia) from the Pliocene Chinchilla Sand, southeast Queensland, Australia. *Alcheringa: An Australasian Journal of Palaeontology* **39**, 394-406 (2015).

52. Stirling E. On the identity of *Phascolomys* (*Phascolonus*) *gigas*, Owen, and *Sceparnodon ramsayi*, Owen, with a description with some parts of its skeleton. *Memoirs of the Royal Society of South Australia* **1**, 127-178 (1913).

53. Huxley TH. On the premolar teeth of *Diprotodon*, and on a new species of that genus. *Quarterly Journal of the Geological Society* **18**, 422-427 (1862).

54. Price GJ, Sobbe IH. Morphological variation within an individual Pleistocene *Diprotodon optatum* Owen, 1838 (Diprotodontinae; Marsupialia): implications for taxonomy within diprotodontoids. *Alcheringa* **35**, 21-29 (2011).

55. Price GJ. Taxonomy and palaeobiology of the largest-ever marsupial, *Diprotodon* (Diprotodontidae, Marsupialia). *Zoological Journal of the Linnean Society* **153**, 369-397 (2008).

56. Price GJ, Piper KJ. Gigantism of the Australian *Diprotodon* Owen 1838 (Marsupialia, Diprotodontoidea) through the Pleistocene. *Journal of Quaternary Science: Published for the Quaternary Research Association* **24**, 1029-1038 (2009).

57. Pledge N, Prescott J, Hutton J. A late Pleistocene occurrence of *Diprotodon* at Hallett Cove, South Australia. *Transactions of the Royal Society of South Australia* **126**, 39-44 (2002).

58. Helgen KM, Wells RT, Kear BP, Gerdtz WR, Flannery TF. Ecological and evolutionary significance of sizes of giant extinct kangaroos. *Australian Journal of Zoology* **54**, (2006).

59. Croke J, Jansen JD, Amos K, Pietsch TJ. A 100 ka record of fluvial activity in the Fitzroy River Basin, tropical northeastern Australia. *Quaternary Science Reviews* **30**, 1681-1695 (2011).

60. Folk RL. The distinction between grain size and mineral composition in sedimentary-rock nomenclature. *The Journal of Geology* **62**, 344-359 (1954).

61. Aitken MJ. *An introduction to optical dating: the dating of Quaternary sediments by the use of photon-stimulated luminescence*. Oxford University Press (1998).

62. Murray AS, Wintle AG. Luminescence dating of quartz using an improved single-aliquot regenerative-dose protocol. *Radiation measurements* **32**, 57-73 (2000).

63. Demuro M, Arnold LJ, Aranburu A, Gomez-Olivencia A, Arsuaga J-L. Single-grain OSL dating of the Middle Palaeolithic site of Galería de las Estatuas, Atapuerca (Burgos, Spain). *Quaternary Geochronology* **49**, 254-261 (2019).

64. Arnold LJ, Duval M, Demuro M, Spooner NA, Santonja M, Pérez-González A. OSL dating of individual quartz 'supergrains' from the Ancient Middle Palaeolithic site of Cuesta de la Bajada, Spain. *Quaternary Geochronology* **36**, 78-101 (2016).

65. Demuro M, Arnold LJ, Aranburu A, Sala N, Arsuaga J-L. New bracketing luminescence ages constrain the Sima de los Huesos hominin fossils (Atapuerca, Spain) to MIS 12. *Journal of Human Evolution* **131**, 76-95 (2019).

66. Duller GAT. Distinguishing quartz and feldspar in single grain luminescence measurements. *Radiation measurements* **37**, 161-165 (2003).

67. Pietsch TJ. Optically stimulated luminescence dating of young (< 500 years old) sediments: Testing estimates of burial dose. *Quaternary Geochronology* **4**, 406-422 (2009).

68. Pietsch TJ, Nanson GC, Olley JM. Late Quaternary changes in flow-regime on the Gwydir distributive fluvial system, southeastern Australia. *Quaternary Science Reviews* **69**, 168-180 (2013).

69. Arnold L, Roberts R. Stochastic modelling of multi-grain equivalent dose (De) distributions: Implications for OSL dating of sediment mixtures. *Quaternary Geochronology* **4**, 204-230 (2009).

70. Arnold LJ, Bailey RM, Tucker GE. Statistical treatment of fluvial dose distributions from southern Colorado arroyo deposits. *Quaternary Geochronology* **2**, 162-167 (2007).

71. Arnold LJ, Roberts RG, MacPhee RDE, Willerslev E, Tikhonov AN, Brock F. Optical dating of perennially frozen deposits associated with preserved ancient plant and animal DNA in north-central Siberia. *Quaternary Geochronology* **3**, 114-136 (2008).

72. Arnold LJ, Demuro M, Navazo M, Benito-Calvo A, Pérez-González A. OSL dating of the Middle Palaeolithic Hotel California site, Sierra de Atapuerca, north-central Spain. *Boreas* **42**, 285-305 (2013).

73. Nathan RP, Thomas PJ, Jain M, Murray AS, Rhodes EJ. Environmental dose rate heterogeneity of beta radiation and its implications for luminescence dating: Monte Carlo modelling and experimental validation. *Radiation Measurements* **37**, 305-313 (2003).

74. Mayya YS, Morthekai P, Murari MK, Singhvi AK. Towards quantifying beta microdosimetric effects in single-grain quartz dose distribution. *Radiation Measurements* **41**, 1032-1039 (2006).

75. Arnold LJ*, et al.* Luminescence dating and palaeomagnetic age constraint on hominins from Sima de los Huesos, Atapuerca, Spain. *Journal of Human Evolution* **67**, 85-107 (2014).

76. Galbraith RF, Roberts RG, Laslett GM, Yoshida H, Olley JM. Optical dating of single and multiple grains of quartz from Jinmium Rock Shelter, Northern Australia: part I, experimental design and statistical models. *Archaeometry* **41**, 339-364 (1999).

77. Bailey RM, Arnold LJ. Statistical modelling of single grain quartz De distributions and an assessment of procedures for estimating burial dose. *Quaternary Science Reviews* **25**, 2475-2502 (2006).

78. Galbraith R, Green P. Estimating the component ages in a finite mixture. *International Journal of Radiation Applications and Instrumentation Part D Nuclear Tracks and Radiation Measurements* **17**, 197-206 (1990).

79. Arnold LJ, Roberts RG, Galbraith RF, DeLong SB. A revised burial dose estimation procedure for optical dating of young and modern-age sediments. *Quaternary Geochronology* **4**, 306-325 (2009).

80. Arnold LJ, Duval M, Falguères C, Bahain JJ, Demuro M. Portable gamma spectrometry with cerium-doped lanthanum bromide scintillators: Suitability assessments for luminescence and electron spin resonance dating applications. *Radiation Measurements* **47**, 6-18 (2012).

81. Bøtter-Jensen L, Bulur E, Duller GAT, Murray AS. Advances in luminescence instrument systems. *Radiation Measurements* **32**, 523-528 (2000).

82. Potts PJ, Thompson M, Chenery SR, Webb PC, Kasper HU. GeoPT13-An international proficiency test for analytical geochemistry laboratories-report on round 13/July 2003 (Köln Loess). *International Association of Geoanalysts*, (2003).

83. Prescott JR, Hutton JT. Cosmic ray contributions to dose rates for luminescence and ESR dating: large depths and long-term time variations. *Radiation measurements* **23**, 497-500 (1994).

84. Mejdahl V. Internal radioactivity in quartz and feldspar grains. *Ancient TL* **5**, 10-17 (1987).

85. Bowler J*, et al.* New ages for human occupation and climatic change at Lake Mungo, Australia. *Nature* **421**, 837 (2003).

86. Jacobs Z, Duller GA, Wintle AG, Henshilwood CS. Extending the chronology of deposits at Blombos Cave, South Africa, back to 140 ka using optical dating of single and multiple grains of quartz. *Journal of Human Evolution* **51**, 255-273 (2006).

87. Pawley SM*, et al.* Age limits on Middle Pleistocene glacial sediments from OSL dating, north Norfolk, UK. *Quaternary Science Reviews* **27**, 1363-1377 (2008).

88. Rees-Jones J. Optical dating of young sediments using fine-grain quartz. *Ancient TL* **13**, 9-14 (1995).

89. Rees-Jones J, Tite MS. Optical dating results for British archaeological sediments. *Archaeometry* **39**, 177-187 (1997).

90. Guérin G, Mercier N, Adamiec G. Dose-rate conversion factors: update. *Ancient TL* **29**, 5-8 (2011).

91. Stokes S, Ingram S, Aitken MJ, Sirocko F, Anderson R, Leuschner D. Alternative chronologies for Late Quaternary (Last Interglacial–Holocene) deep sea sediments via optical dating of silt-sized quartz. *Quaternary Science Reviews* **22**, 925-941 (2003).

92. Mejdahl V. Thermoluminescence dating: beta-dose attenuation in quartz grains. *Archaeometry* **21**, 61-72 (1979).

93. Aitken MJ. *Thermoluminescence dating*. Academic Press (1985).

94. Brennan BJ. Beta doses to spherical grains. *Radiation Measurements* **37**, 299-303 (2003).

95. Olley JM, Murray A, Roberts RG. The effects of disequilibria in the uranium and thorium decay chains on burial dose rates in fluvial sediments. *Quaternary Science Reviews* **15**, 751-760 (1996).

96. Olley JM, Roberts RG, Murray AS. Disequilibria in the uranium decay series in sedimentary deposits at Allen's Cave, Nullarbor Plain, Australia: implications for dose rate determinations. *Radiation Measurements* **27**, 433-443 (1997).

97. Preusser F, Degering D. Luminescence dating of the Niederweningen mammoth site, Switzerland. *Quaternary International* **164-165**, 106-112 (2007).

98. Galbraith RF, Roberts RG. Statistical aspects of equivalent dose and error calculation and display in OSL dating: an overview and some recommendations. *Quaternary Geochronology* **11**, 1-27 (2012).

99. Roberts RG, Galbraith R, Yoshida H, Laslett G, Olley JM. Distinguishing dose populations in sediment mixtures: a test of single-grain optical dating procedures using mixtures of laboratory-dosed quartz. *Radiation Measurements* **32**, 459-465 (2000).

100. Stokes S, Ingram S, Aitken M, Sirocko F, Anderson R, Leuschner D. Alternative chronologies for Late Quaternary (Last Interglacial–Holocene) deep sea sediments via optical dating of silt-sized quartz. *Quaternary Science Reviews* **22**, 925-941 (2003).

101. Bowler JM*, et al.* New ages for human occupation and climatic change at Lake Mungo, Australia. *Nature* **421**, 837-840 (2003).

102. Jacobs Z, Duller GA, Wintle AG. Interpretation of single grain De distributions and calculation of De. *Radiation Measurements* **41**, 264-277 (2006).

103. Brock F, Wood R, Higham TF, Ditchfield P, Bayliss A, Ramsey CB. Reliability of nitrogen content (% N) and carbon: nitrogen atomic ratios (C: N) as indicators of collagen preservation suitable for radiocarbon dating. *Radiocarbon* **54**, 879-886 (2012).

104. Fowler AJ, Gillespie R, Hedges RE. Radiocarbon dating of sediments. *Radiocarbon* **28**, 441-450 (1986).

105. McGeehin J*, et al.* Stepped-combustion 14 C dating of sediment: a comparison with established techniques. *Radiocarbon* **43**, 255-261 (2001).

106. Wakeham S, Canuel E. The nature of organic carbon in density-fractionated sediments in the Sacramento-San Joaquin River Delta (California). *Biogeosciences* **13**, 567 (2016).

107. Bird MI*, et al.* Radiocarbon dating of "old" charcoal using a wet oxidation, stepped-combustion procedure. *Radiocarbon* **41**, 127-140 (1999).

108. Wood R, Jacobs Z, Vannieuwenhuyse D, Balme J, O'Connor S, Whitau R. Towards an accurate and precise chronology for the colonization of Australia: The example of Riwi, Kimberley, Western Australia. *PloS one* **11**, e0160123 (2016).

109. Fallon S, Fifield LK, Chappell J. The next chapter in radiocarbon dating at the Australian National University: status report on the single stage AMS. *Nuclear Instruments and Methods in Physics Research Section B: Beam Interactions with Materials and Atoms* **268**, 898-901 (2010).

110. Stuiver M, Polach HA. Discussion reporting of 14 C data. *Radiocarbon* **19**, 355-363 (1977).

111. Hogg AG*, et al.* SHCal13 Southern Hemisphere calibration, 0–50,000 years cal BP. *Radiocarbon* **55**, 1889-1903 (2013).

112. Bronk Ramsey C. Bayesian analysis of radiocarbon dates. Radiocarbon51 (1): 337–360. (ed^(eds) (2009).

113. Blong RJ, Gillespie R. Fluvially transported charcoal gives erroneous 14C ages for recent deposits. *Nature* **271**, 739 (1978).

114. Frueh WT, Lancaster ST. Correction of deposit ages for inherited ages of charcoal: implications for sediment dynamics inferred from random sampling of deposits on headwater valley floors. *Quaternary Science Reviews* **88**, 110-124 (2014).

115. Martin L, Goff J, Jacobsen G, Mooney S. The radiocarbon ages of different organic components in the Mires of Eastern Australia. *Radiocarbon* **61**, 173-184 (2019).

116. Price GJ, Feng Y-x, Zhao J-x, Webb GE. Direct U–Th dating of vertebrate fossils with minimum sampling destruction and application to museum specimens. *Quaternary Geochronology* **18**, 1-8 (2013).

117. Ludwig KR*, et al.* Mass-spectrometric 230Th-234U-238U dating of the Devils Hole calcite vein. *Science* **258**, 284-287 (1992).

118. Ludwig KR. User's manual for isoplot 3.00, a geochronlogical toolkit for microsoft excel. *Berkeley Geochronl Cent Spec Publ* **4**, 25-32 (2003).

119. Cheng H, Edwards RL, Hoff J, Gallup CD, Richards D, Asmerom Y. The half-lives of uranium-234 and thorium-230. *Chemical Geology* **169**, 17-33 (2000).

120. Bernal J-P, Eggins SM, McCulloch MT. Accurate in situ 238 U–234 U–232 Th–230 Th analysis of silicate glasses and iron oxides by laser-ablation MC-ICP-MS. *Journal of Analytical Atomic Spectrometry* **20**, 1240-1249 (2005).

121. Woodroffe CD, Short SA, Stoddart DR, Spencer T, Harmon RS. Stratigraphy and chronology of late Pleistocene reefs in the southern Cook Islands, south Pacific. *Quaternary Research* **35**, 246-263 (1991).

122. Sambridge M, Grün R, Eggins S. U-series dating of bone in an open system: the diffusion-adsorption-decay model. *Quaternary Geochronology* **9**, 42-53 (2012).

123. Dosseto A, Marwick B. UThwigl - an R package for closed- and open-system uranium-thorium dating. (ed^(eds) (2019).

124. Shao Q, Bahain J-J, Dolo J-M, Falguères C. Monte Carlo approach to calculate US-ESR age and age uncertainty for tooth enamel. *Quaternary Geochronology* **22**, 99-106 (2014).

125. Joannes-Boyau R, Grün R. A comprehensive model for CO2− radicals in fossil tooth enamel: implications for ESR dating. *Quaternary Geochronology* **6**, 82-97 (2011).

126. Joannes-Boyau R. Detailed protocol for an accurate non-destructive direct dating of tooth enamel fragment using Electron Spin Resonance. *Geochronometria* **40**, 322-333 (2013).

127. Joannes-Boyau R, Grün R. Thermal behavior of orientated and non-orientated CO2− radicals in tooth enamel. *Radiation Measurements* **44**, 505-511 (2009).

128. Joannes-Boyau R, Duval M, Bodin T. MCDoseE 2.0 A new Markov Chain Monte Carlo program for ESR dose response curve fitting and dose evaluation. *Quaternary Geochronology* **44**, 13-22 (2018).

129. Metropolis N, Rosenbluth AW, Rosenbluth MN, Teller AH, Teller E. Equation of state calculations by fast computing machines. *The journal of chemical physics* **21**, 1087-1092 (1953).

130. Duval M, Grün R. Are published ESR dose assessments on fossil tooth enamel reliable? *Quaternary Geochronology* **31**, 19-27 (2016).

131. Prescott JR, Hutton JT. Cosmic ray contributions to dose rates for luminescence and ESR dating: large depths and long-term time variations. *Radiation measurements* **23**, 497-500 (1994).

132. Moss PT. Palynology and its application to geomorphology. In: *Treatise on Geomorphology* (ed^(eds Shroder JF, Switzer, A.D., Kennedy, D.M.). Academic Press (2013).

133. Moss PT, Tibby J, Petherick L, McGowan H, Barr C. Late Quaternary vegetation history of North Stradbroke Island, Queensland, eastern Australia. *Quaternary Science Reviews* **74**, 257-272 (2013).

134. Moss PT, Dunbar GB, Thomas Z, Turney C, Kershaw AP, Jacobsen GE. A 60 000-year record of environmental change for the Wet Tropics of north-eastern Australia based on the ODP 820 marine core. *Journal of Quaternary Science* **32**, 704-716 (2017).

135. Rule S, Brook BW, Haberle SG, Turney CS, Kershaw AP, Johnson CN. The aftermath of megafaunal extinction: ecosystem transformation in Pleistocene Australia. *Science* **335**, 1483-1486 (2012).
