# Supplementary material for: Extinction of eastern Sahul megafauna coincides with sustained environmental deterioration
Source: Nat Commun. 2020 May 18;11:2250. doi: 10.1038/s41467-020-15785-w (PMC7231803; doi:10.1038/s41467-020-15785-w)
Supplement: Supplementary file 3 — Reporting Summary [file 41467_2020_15785_MOESM3_ESM.pdf]

nature research

Corresponding author(s): Scott Alexander Hocknull

Last updated by author(s): Feb 25, 2020

Reporting Summary

Nature Research wishes to improve the reproducibility of the work that we publish. This form provides structure for consistency and transparency in reporting. For further information on Nature Research policies, see Authors & Referees and the Editorial Policy Checklist .

Statistics

For all statistical analyses, confirm that the following items are present in the figure legend, table legend, main text, or Methods section.

n/a	Confirmed	
x	☐	The exact sample size (n) for each experimental group/condition, given as a discrete number and unit of measurement
x	☐	A statement on whether measurements were taken from distinct samples or whether the same sample was measured repeatedly
x	☐	The statistical test(s) used AND whether they are one- or two-sided<br>*Only common tests should be described solely by name; describe more complex techniques in the Methods section.*
x	☐	A description of all covariates tested
x	☐	A description of any assumptions or corrections, such as tests of normality and adjustment for multiple comparisons
☐	x	A full description of the statistical parameters including central tendency (e.g. means) or other basic estimates (e.g. regression coefficient) AND variation (e.g. standard deviation) or associated estimates of uncertainty (e.g. confidence intervals)
x	☐	For null hypothesis testing, the test statistic (e.g. F, t, r) with confidence intervals, effect sizes, degrees of freedom and P value noted<br>*Give P values as exact values whenever suitable.*
☐	x	For Bayesian analysis, information on the choice of priors and Markov chain Monte Carlo settings
x	☐	For hierarchical and complex designs, identification of the appropriate level for tests and full reporting of outcomes
x	☐	Estimates of effect sizes (e.g. Cohen's d, Pearson's r), indicating how they were calculated

Our web collection on statistics for biologists contains articles on many of the points above.

Software and code

Policy information about availability of computer code

Data collection	Microsoft Excel, 2010; PAST (Palaeontological Statistical Software) 3.14; Corel Suite 16; Lightroom 4.4; Freiberg ESR software; TL/OSL for Windows 4.47; QGIS
Data analysis	Computed Tomography data (InVesalius 3.1); R; ESR dating (McDoseE 2.0); U-Series (Iolite v2); OxCal 4.3; Luminescence Analyst 4.31.9; RStudio 3.3.3; Agisoft Metashape.

For manuscripts utilizing custom algorithms or software that are central to the research but not yet described in published literature, software must be made available to editors/reviewers. We strongly encourage code deposition in a community repository (e.g. GitHub). See the Nature Research guidelines for submitting code & software for further information.

Data

Policy information about availability of data

All manuscripts must include a data availability statement. This statement should provide the following information, where applicable:

- Accession codes, unique identifiers, or web links for publicly available datasets
- A list of figures that have associated raw data
- A description of any restrictions on data availability

The authors declare that all data supporting the findings of this study are available within the paper and its supplementary information files.

Field-specific reporting

Please select the one below that is the best fit for your research. If you are not sure, read the appropriate sections before making your selection.

☐ Life sciences ☐ Behavioural & social sciences x Ecological, evolutionary & environmental sciences

Ecological, evolutionary & environmental sciences study design

All studies must disclose on these points even when the disclosure is negative.

Study description	This study presents new reliably-dated fossil records of unique and diverse megafauna from eastern Sahul. These new records are used to test and resolve currently polarised interpretations of continental-wide extinction of megafauna in Sahul. This study uses multiple techniques to date the fossils and a detailed assessment of new faunal remains.
Research sample	Fossil samples of fauna and flora were collected from fossiliferous sedimentary units within the South Walker Creek system. Sediment cores, charcoal, bone and tooth samples were extracted from these same deposits so that a multidisciplinary assessment could be undertaken to verify the primary context and age of the remains within the deposits.
Sampling strategy	Fossil remains were recovered from sites and fossiliferous units through direct surface retrieval and excavation. Samples recovered for dating, in particular OSL were recovered using standard OSL techniques as outlined in the Supplementary Information.
Data collection	Fossils, sediment and stratigraphic information was collected using standard palaeontological excavation techniques by the field teams undertaking the work. This included Queensland Museum Geosciences staff (Scott Hocknull & Rochelle Lawrence) as field leaders. OSL samples were collected by Queensland Museum staff (Scott Hocknull & Rochelle Lawrence) and Richard Lewis from the University of Adelaide. ESR dating was performed at Southern Cross University Renaud Joannes-Boyau following the protocol outlined in the supplementary information and in previous published work. U-series dating samples were collected and performed by University of Queensland (Gilbert Price) and University of Wollongong (Anthony Dosseto) following protocols outlined in the supplementary information. OSL samples and dating were collected and performed by Griffith University (Tim Piestch) and University of Adelaide (Richard Lewis & Lee Arnold). Radiocarbon samples were collected by Queensland Museum Scott Hocknull and Rochelle Lawrence and performed by Beta Analytic and Australian National University (Rachel Wood). Palynological samples were collected by Scott Hocknull and Rochelle Lawrence and assessed at University of Queensland (Patrick Moss).
Timing and spatial scale	Sampling occurred from 2008 to 2017 with annual fieldwork, with on-foot surveys determining the distribution of available sites to focus sampling on within the South Walker Creek area.
Data exclusions	For open-system U-series age modelling, data from the enamel of sample P-SCU01 were excluded because of the low uranium contents. Radiocarbon dates on sediment, outlined in the supplementary information, were not included in the analysis and are excluded from Table 1 & 2 in the main text but provided in the Supplementary Information. This sample type is inappropriate for radiocarbon as multiple sources of carbon of varying age are present, making age estimates impossible to interpret. References and details are given in the text
Reproducibility	Reproducibility of in-situ U-series measurements at UOW was assessed by repeat analysis of our in-house coral standard. 4 dating methods (OSL, U-series, ESR, c14) were used to assess whether findings were consistent.<br><br>Radiocarbon dating (c14) of charcoal: The chemical treatment given to the charcoal does not always completely remove contamination, causing ages estimates to underestimate the true age of the sample. This needed to be applied due to the small size and poor preservation of the charcoal. It means that the 3 age estimates obtained are not reproducible. However, we have interpreted them as minimum age estimates only.
Randomization	No randomizations were possible.
Blinding	No blinding tests were possible.

Did the study involve field work? ☒ Yes ☐ No

Field work, collection and transport

Field conditions	New fossils, sediment samples and dating samples were recovered in the field over the period spanning 2008-2017. All fieldwork was coordinated through Queensland Museum Geosciences staff. The environment is dry and tropical.
Location	All samples were collected along a reach of Walker Creek, within the South Walker Creek Mine, near the township of Nebo, west of Mackay, Queensland, Australia.
Access and import/export	All access was permitted through Queensland Museum field collection protocols as the State collection for fossils. Access to sites was granted by BHP (mine lease), local land holders and traditional owners (Barada Barna).
Disturbance	Excavations were undertaken from 2008 onwards to record and salvage sites close to and within the mining lease for South Walker Creek Mine, therefore, salvage of each site through excavation (surface disturbance) was necessary to recover the specimens and preserve them in perpetuity.

Reporting for specific materials, systems and methods

We require information from authors about some types of materials, experimental systems and methods used in many studies. Here, indicate whether each material, system or method listed is relevant to your study. If you are not sure if a list item applies to your research, read the appropriate section before selecting a response.

Materials & experimental systems

n/a	Involved in the study
☒	☐ Antibodies
☒	☐ Eukaryotic cell lines
☐	☒ Palaeontology
☒	☐ Animals and other organisms
☒	☐ Human research participants
☒	☐ Clinical data

Methods

n/a	Involved in the study
☒	☐ ChIP-seq
☒	☐ Flow cytometry
☒	☐ MRI-based neuroimaging

Palaeontology

Specimen provenance

The provenance of specimens used in this study are from Queensland, with the exception of two macropodid specimens from Victoria. All specimens collected in Queensland were permitted through Queensland Museum State Collection fieldwork procedures and did not occur on protected lands where scientific collecting permits are required. The two specimens from Victoria were accessed through the Museum Victoria State Collection.

Specimen deposition

All specimens collected in Queensland are deposited in the Queensland Museum, accessioned with a Queensland Museum Fossil (QMF) number as detailed in the Supplementary Information. Two specimens are deposited in Museum Victoria collections and accession numbers are provided for these within the Supplementary Information. All specimens are available for access through each State museum collection management process, with the collections based in Brisbane (Queensland Museum) and Melbourne (Museum Victoria).

Dating methods

New dates are provided within this work and utilise the following techniques: Optically Stimulated Luminescence (OSL); Electron Spin Resonance (ESR); radiometric Uranium-series dating (U-series) using micro-drilling and laser ablation; radiocarbon (c14) dating. Radiocarbon dates on charcoal were produced at the Australian National University Radiocarbon Facility (laboratory code S-ANU). An acid-base-acid pretreatment was applied as the samples were small and/or poorly preserved. Samples were converted to graphite and measured by AMS. Radiocarbon dates on sediment were analysed at Beta-Analytic. Pretreatment involved an acid wash and samples were measured by AMS. Calibration was undertaken in OxCal against SHCal13. All methodological details are provided in the main text Methods section and Supplementary Information.

☒ Tick this box to confirm that the raw and calibrated dates are available in the paper or in Supplementary Information.
